# A dataset of the flowering plants (Angiospermae) in urban green areas in five European cities

**DOI:** 10.1016/j.dib.2021.107243

**Published:** 2021-06-25

**Authors:** Joan Casanelles-Abella, David Frey, Stefanie Müller, Cristiana Aleixo, Marta Alós Ortí, Nicolas Deguines, Tiit Hallikma, Lauri Laanisto, Ülo Niinemets, Pedro Pinho, Roeland Samson, Lucía Villarroya-Villalba, Marco Moretti

**Affiliations:** a Biodiversity and Conservation Biology, Swiss Federal Research Institute WSL, Birmensdorf, Switzerland; b Landscape Ecology, Institute of Terrestrial Ecosystems, ETH Zürich, Zürich, Switzerland; c Department of Evolutionary Biology and Environmental Studies, University of Zürich, Zürich, Switzerland; d Centre for Ecology, Evolution and Environmental Changes (cE3c), Faculdade de Ciências, Universidade de Lisboa, Lisboa, Portugal; e Institute of Agricultural and Environmental Sciences, Estonian University of Life Sciences, Tartu, Estonia; f Université Paris-Saclay, CNRS, AgroParisTech, Ecologie Systématique Evolution, Orsay, France; g Laboratoire Ecologie et Biologie des Interactions, Equipe Ecologie Evolution Symbiose, Université de Poitiers, UMR CNRS 7267, France; h Lab of Environmental and Urban Ecology, Research Group Environmental Ecology & Microbiology (ENdEMIC), Dept. Bioscience Engineering, University of Antwerp, Antwerp, Belgium

**Keywords:** Urban biodiversity, Urban green spaces, Urban flora, Plants, Gardening, Urban green infrastructure, Plant traits, Floral traits, Fragmentation

## Abstract

This article summarizes the data of a survey of flowering plants in 80 sites in five European cities and urban agglomerations (Antwerp, Belgium; greater Paris, France; Poznan, Poland; Tartu, Estonia; and Zurich, Switzerland). Sampling sites were selected based on a double orthogonal gradient of size and connectivity and were urban green areas (e.g. parks, cemeteries). To characterize the flowering plants, two sampling methodologies were applied between April and July 2018. First, a floristic inventory of the occurrence of all flowering plants in the five cities. Second, flower counts in sampling plots of standardized size (1 m^2^) only in Zurich. We sampled 2146 plant species (contained in 824 genera and 137 families) and across the five cities. For each plant species, we provide its origin status (i.e. whether the plants are native from Europe or not) and 11 functional traits potentially important for plant-pollinator interactions. For each study site, we provide the number of species, genera, and families recorded, the Shannon diversity as well as the proportion of exotic species, herbs, shrubs and trees. In addition, we provide information on the patch size, connectivity, and urban intensity, using four remote sensing-based proxies measured at 100- and 800-m radii.

## Specifications Table

**Table:** 

Subject	Ecology, Nature, and Landscape Conservation.
Specific subject area	Urban ecology
Type of data	Table Fig.
How data were acquired	Floristic inventories and standardized floral counts. Satellite data.
Data format	Raw and aggregated
Parameters for data collection	Sites were selected from the European Urban Atlas, using the features mapped as green areas. Sites were chosen following an orthogonal gradient of patch size and connectivity inferred with the proximity index. We selected 32 sites in Zurich, Switzerland, and 12 sites in each of the remaining four cities (i.e. Antwerp, Paris, Poznan and Tartu).
Description of data collection	We applied two sampling methodologies inside of buffers of 100 m radius: 1) a floristic inventory of the occurrence of all flowering plants of potential interest for pollinators performed in the five cities, and 2) flower counts in sampling plots of standardized size (1 m^2^) done only in Zurich. Sites were visited on three occasions between April and July 2018. The duration of each visit was restricted to a maximum of 2.5 h.
Data source location	City of Antwerp, Belgium; *51°15′N, 4°24′E* Greater Paris, France; *48°51′N, 8°05′E* City of Poznan, Poland; *52°24′N, 16°55′E* City of Tartu, Estonia; *58°22′N, 26°43′E* City of Zurich, Switzerland; *47°22′N, 8°33′E*
Data accessibility	Repository name: Envidat Data identification number: doi:10.16904/envidat.210 Direct URL to data: https://www.envidat.ch/dataset/flowering-plants-angiospermae-in-urban-green-areas-in-five-european-cities File 1: Floral_1_occurrence.csv contains the list of plant species sampled in the five cities during the different sampling periods. File 2: Floral_2_counts.csv contains the floral units, mean number of flowers per floral units and the floral abundance of the different plants counted in quadrats in the study sites in Zurich during four sampling periods. File 3: Floral_traits.csv contains the trait values extracted from the literature for the sampled plants.

## Value of the Data

• The dataset describes the diversity, occurrence, and floral counts of a large number of flowering plant families sampled in a standardized way in different types of public and private green areas in European cities, and with a high taxonomic resolution.
• The data contribute characterizing European urban floras, derive taxonomic, phylogenetic and trait diversity patterns, and perform comparative studies among different cities, different types of urban green areas and in fragmentation studies.
• The data can be used to characterize the available food resources of other trophic levels, particularly pollinators, and species interactions.
• The data on floral counts can be combined with metrics on nectar and pollen content to obtain estimates of resources quality (e.g. as done in [1])
• The methodology for collecting the data can be applied in further studies aiming to characterize plant resources in one or more urban ecosystems in a standardized way.

## 1 Data Description

**Fig. 1:**
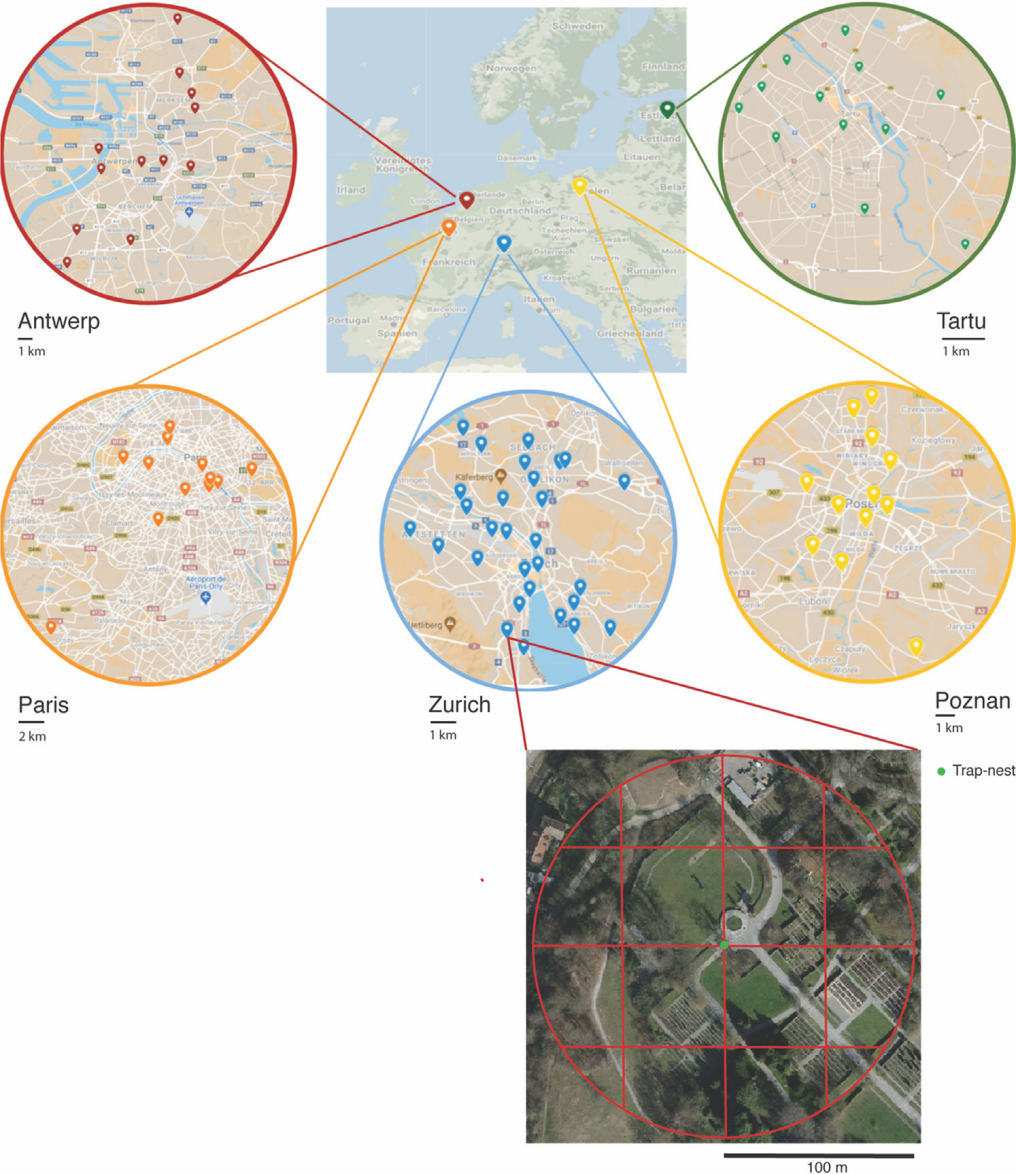
Maps of the study sites in each of the five cities (Antwerp, Greater Paris, Poznan, Tartu and Zurich) and an example of how the sampling was conducted. For the site Zu006 (located in Zurich), we show the trap-nest location (green dot), the 100 m radius buffer around it and the 16 cells dividing the buffer.

The paper presents the data of a plant survey in urban green areas from five European cities and urban agglomerations (Antwerp, Belgium; greater Paris, France; Poznan, Poland; and Zurich, Switzerland). 80 sites were selected (32 in Zurich and 12 in each of the remaining four cities, see Fig. 1) according to an orthogonal gradient of patch size and connectivity (see Section 2.2), representing common public urban green areas such as parks, cemeteries and gardens. To characterize the flowering plants, we sampled plants during four (for Zurich) and three (for Antwerp, Paris, Poznan and Tartu) sampling periods during the year 2018. The sampling was performed in (1) end of April (only for Zurich), (2) end of May, (2) end of June and (3) end of July. The sampling consisted in two methodologies. First, a floristic inventory of the occurrence of all flowering plants inside buffers of 100 m radius (see Fig. 1) in the study sites of the five cities. Second, flower counts of defined floral units (Table 1) in sampling plots of standardized size (1 m^2^) distributed inside buffers of 100 m radius (see Fig. 1) done only in Zurich. The 100 m radius buffer was defined from existing installed trap-nests place to sample cavity-nesting bees and wasps (Fig. 1).

**Table 1:** Definition of the flower units and calculation of the floral abundance. For each floral unit type, we show the plant taxa included and how the floral abundance was calculated. For specific floral unit types (i.e. capitula in Dipsacoidae, compound cymes, corymb, panicles, racemes and umbels) we estimated the number of flowers per floral unit by counting all the flowers in seven floral units and computing the mean. Ditto = the same again.

Floral unit definition	Plant taxa	Estimation number of flowers within a floral unit (Nf)	Floral abundance (Fa)
Single flowers	Acanthaceae, Alismataceae, Amaranthaceae, Anacardiaceae, Apocynaceae, Asparagaceae, Balsaminaceae, Begoniaceae, Boraginaceae, Brassicaceae, Campanulaceae (except *Phyteuma* spp.), Caprifoliaceae (except Dipsacoideae), Caryophyllaceae, Celastraceae, Cistaceae, Cleomaceae, Convolvulaceae, Crassulaceae, Cucurbitaceae, *Cytisus* spp., Geraniaceae, Hypericaceae, Iridiaceae, Lamiaceae, *Lathyrus* spp., Linaceae, Lythraceae, Magnoliaceae, Malvaceae, Onargaceae, Orchidaceae, Orobanchaceae, Oxalidaceae,Papaveraceae, Phrymaceae, Plantaginaceae (except *Plantago* spp.), Polemoniaceae, Polygonaceae, Portulacaceae, Primulaceae, Ranunculaceae, Resedaceae, *Rhododendron* spp., Rosaceae (except *Filipendula ulmaria, Sanguisorba* spp., *Spiraea* spp.), Rutaceae, Saxifragaceae, *Spartium* spp., Solanaceae, Scrophulariaceae (except *Buddleja davidii*), Tropeolaceae, Verbenaceae, Violaceae, Xanthorrhoeaceae	Not applicable	Fa=∑(floralunits)

Single capitulum (in Dipsacoideae)	Dipsacoideae	Estimation in seven different floral units Nf = mean of the seven counts	Fa=∑((floralunits)×Nf)

Single compound cyme	*Centranthus* spp.	Ditto	Ditto

Single corymb	Adoxaceae, Cornaceae	Ditto	Ditto

Single panicle	Sapindaceae*, *Buddleja davidii, Galium* spp.*, Filipendula ulmaria, Sherardia arvensis, Spiraea* spp, *Syringa vulgaris*	Ditto	Ditto
Single raceme	Fabaceae* (except *Cytisus* spp., *Lathyrus* spp., *Spartium* spp.), *Hedera helix, Ligustrum* spp., Vitaceae	Ditto	Ditto

Single secondary umbell	Apiaceae	Ditto	Ditto

Single umbell	*Allium* spp.	Ditto	Ditto

Single capitulum (in Asteraceae)	Asteraceae	Not estimated	Fa=∑(floralunits)

Single catkin	Betulaceae*, Fagaceae*, Salicaceae*	Not estimated	Ditto

Single corymb & single cyme in *Hydragea* spp.	*Hydrangea* spp.	Not estimated	Ditto

Single cyme with cyathia	*Euphorbia* spp.	Not estimated	Ditto

Single dense cluster	*Sanguisorba*	Not estimated	Ditto

Single spike (in *Plantago* spp. & *Tamarix* spp.)	*Plantago* spp.*, Tamarix* spp.	Not estimated	Ditto

⁎ Observation of the tree canopy and the floral counts of the woody species in these families were done from the ground and are a rough estimate.

**Fig. 2:**
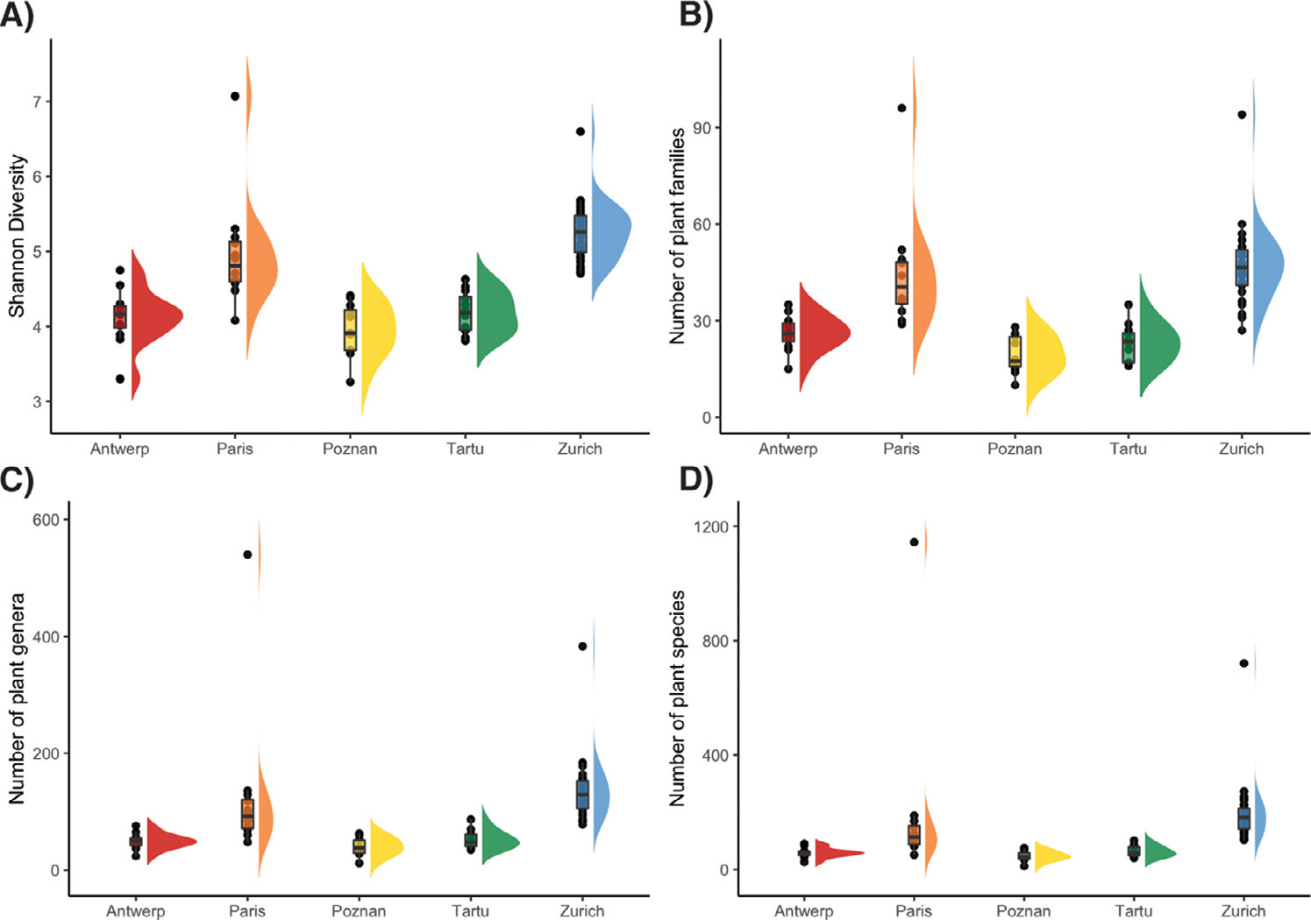
Flat violin [18] and boxplots representing the Shannon diversity (A) and the number of families (B), genera (C), and species (D) recorded in the study sites in Antwerp, Paris, Poznan, Tartu, and Zurich. Each point in a city represents a measurement in one of the sampling sites (12 in Antwerp, Paris, Poznan and Tartu and 32 in Zurich) and in one of the sampling periods (four periods for Zurich and three periods for the remaining four cities). Note that for Paris and Zurich there are two points with larger richness, which corresponds to the study sites in the botanical gardens of Paris (Jardin des Plantes, National Museum of Natural History) and Zurich (Zurich Botanical Garden). Note that the families Cyperaceae, Juncaceae and Poaceae were not included in the sampling.

**Fig. 3:**
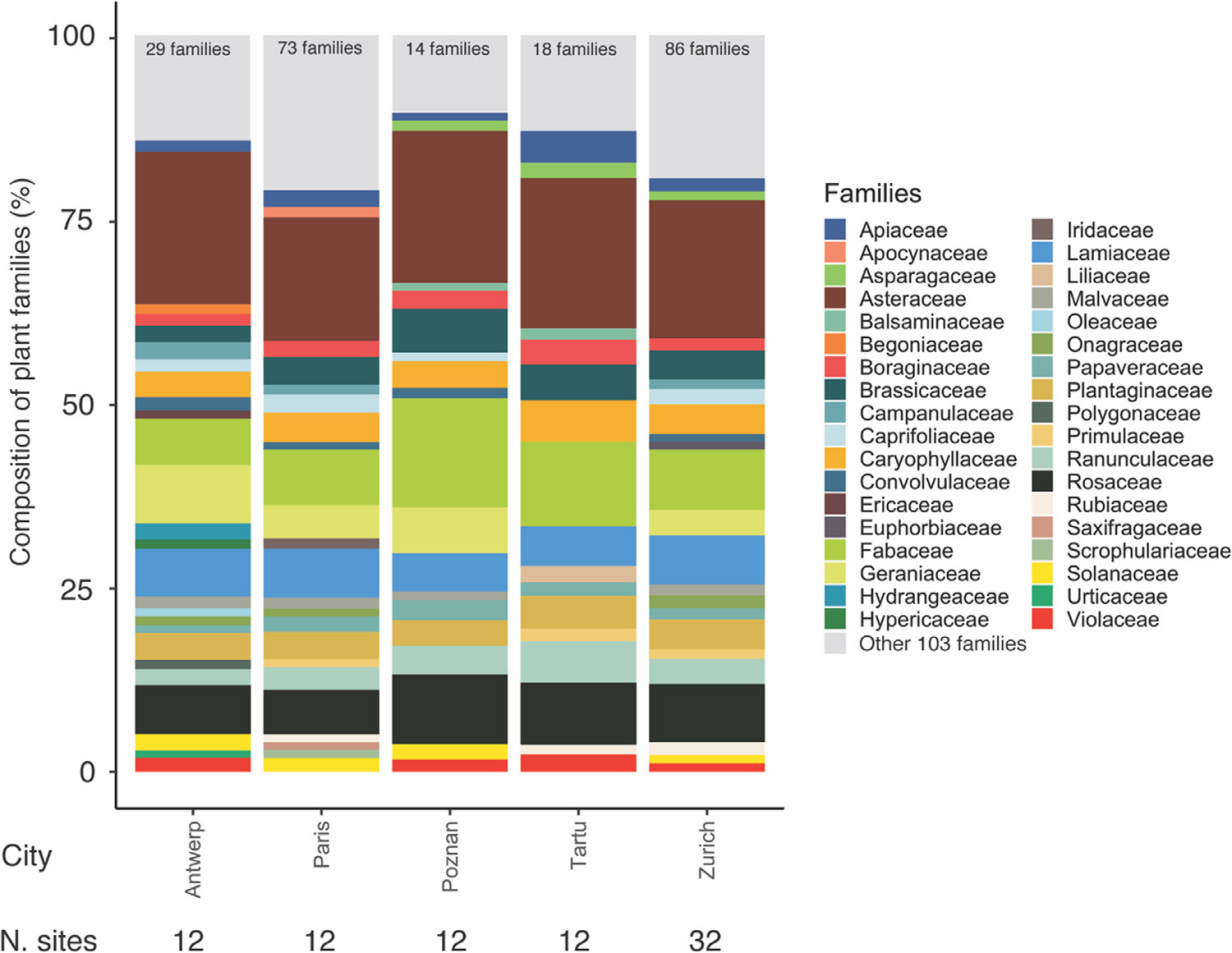
Barplot of the percentage of the different plant families sampled in all study sites (32 in Zurich and 12 in each of the remaining cities) in each city. Only families containing more than 1% of the species sampled in all the study sites and the sampling periods are shown separately. The remaining families are grouped into the category “Other 103 families” (light gray) and the exact number is provided for each city at the top of each bar (i.e. 29 families in Antwerp, 73 families in Paris, 14 families in Poznan, 18 families in Tartu and 86 families in Zurich). Note that the families Cyperaceae, Juncaceae and Poaceae were not included in the sampling.

**Fig. 4:**
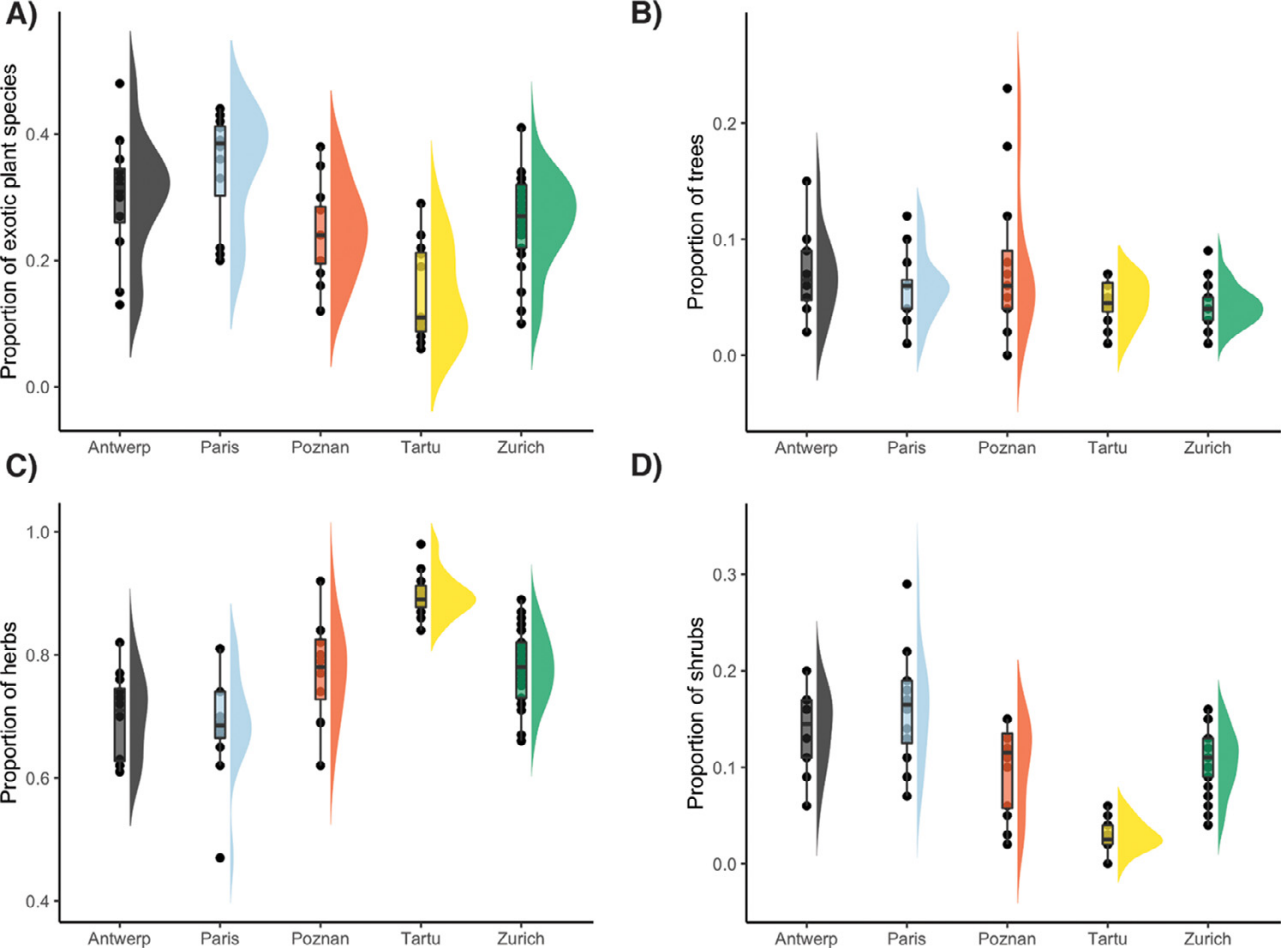
Flat violin [18] and boxplots representing the proportion of exotic plant species (A), trees (B), herbs (C), and shrubs (D) in the study sites in Antwerp, Paris, Poznan, Tartu, and Zurich respectively. Each point in a city represents a measurement in one of the sampling sites (12 in Antwerp, Paris, Poznan and Tartu and 32 in Zurich) and in one of the sampling periods (four periods for Zurich and three periods for the remaining four cities). Note that the families Cyperaceae, Juncaceae and Poaceae were not included in the sampling.

**Fig. 5:**
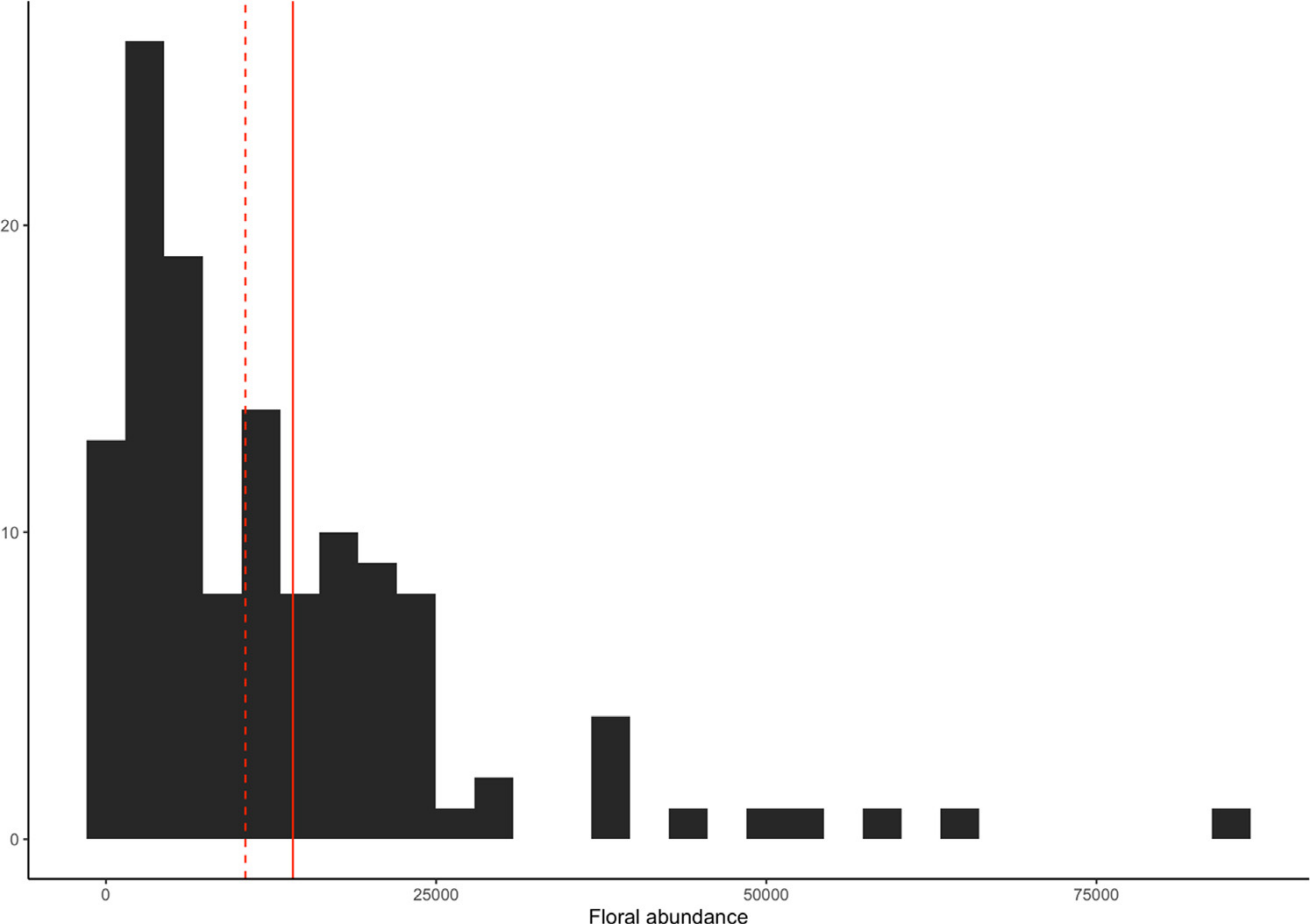
Histogram of the floral counts in the city of Zurich. Floral abundance, shown in the X axis, is calculated as the sum of all the floral units (see Table 1 for the definitions) in all the quadrats for a given site and sampling period, giving a total N of 128 (32 sites x 4 sampling periods). The dashed vertical line represents the median floral abundance and the straight vertical line the mean floral abundance recorded.

**Fig. 6:**
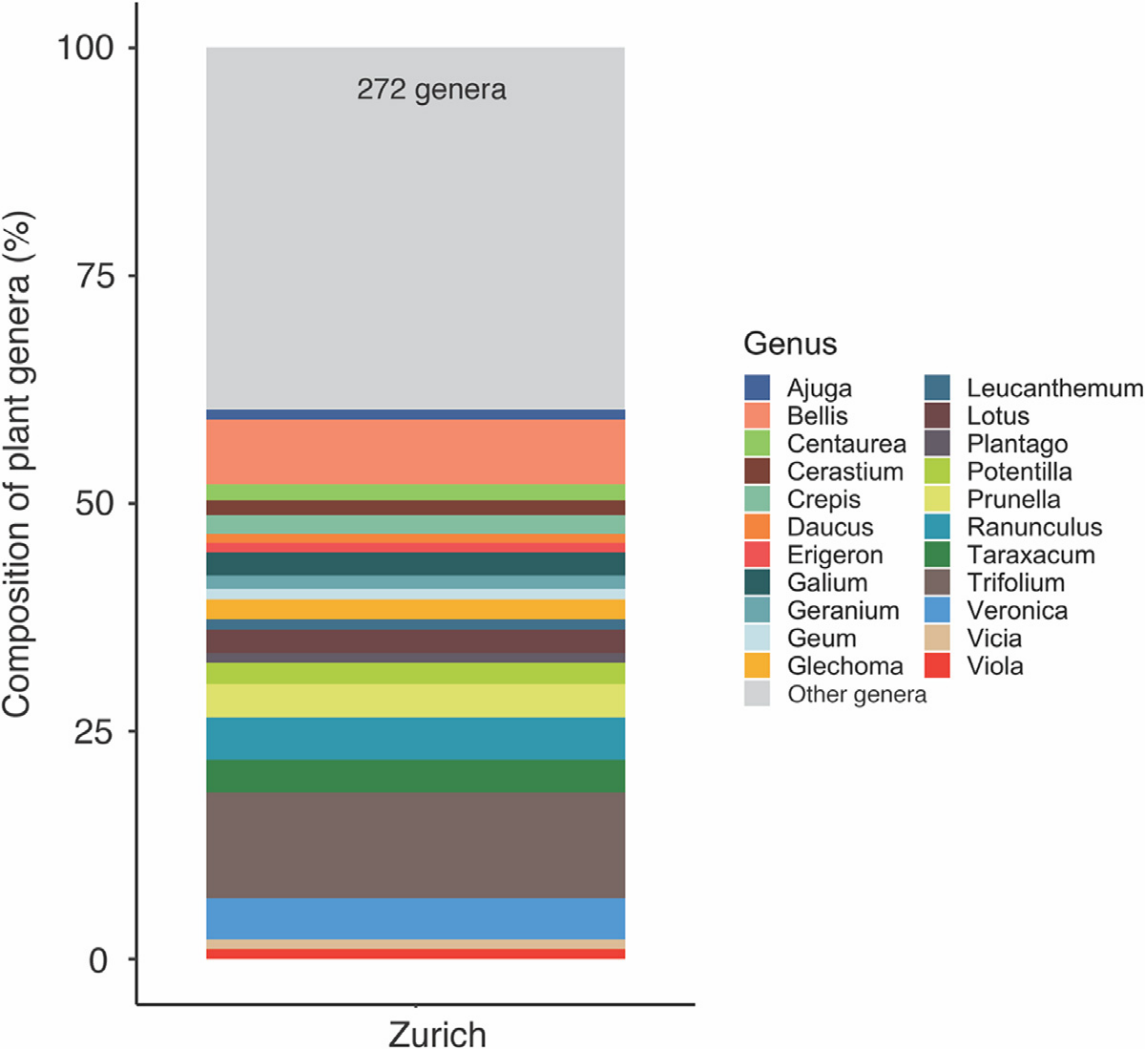
Barplot of the percentage of plant genera in the floral abundance counted in Zurich. Only genera containing more than 1% of the species sampled in all the study sites are shown separately. The remaining genera are grouped into the category “Other genera” (light gray) and the exact number (272) is provided at the top of the bar. Note that the families Cyperaceae, Juncaceae and Poaceae were not included in the sampling.

For each of the 2146 plant species recorded we show in what cities it was recorded (Supplementary material, Table A1). Furthermore, we provide information on 11 traits of potential interest to study plant-pollinator interactions (Table 2) that are the flowering duration, flowering start, growth form, inflorescence type, plant height, floral rewards in the form of nectar, oil and pollen, structural blossom class and floral symmetry based on bibliographic information. Additionally, we documented the origin status of all the sampled plant species, that is, whether or not they are native from Europe. We computed the species, genera, and family richness for each site (Table 3 and Fig. 2) and the composition of plant families of the species sampled in each city (Fig. 3). Moreover, we computed the proportion of exotic species, as well as the proportion of trees, shrubs, and herbs for each site and city (Table 3 and Fig. 4). In addition, we show the frequency distribution of floral counts (Fig. 5) and the composition of plant genera in the flower abundance (Fig. 6) in the city of Zurich.

**Table 2:** List of the 11 traits included. For each trait together, there is a description, the taken values, and references of the sources used to build the trait table. See also Section 2.7 Traits.

Trait	Description	Values	References
Flowering duration	Number of months a plant species flower.	1–12	[4], [5], [6], [7], [8]

Flowering start	The month the blossom of a plant species begins to flower.	1–12	[4], [5], [6], [7], [8]

Growth form	Classification of plant species in four broad growth form categories.	Herb Shrub Tree Climber	[6,[9], [10], [11]

Inflorescence type	Determines whether the blossom is a single flower or an inflorescence.	With inflorescence Without inflorescence	[4], [5], [6],12]

Plant height (m)	Measure of the height of a plant species in meters.		[4,^6^,^9^,10]

Pollination mode	Definition whether a plant species is biotically or abiotically pollinated.	Biotic Abiotic	[6,9]

Rewards: nectar	Describes whether the plant provides nectar resources.	Absent Present	[4,^5^,^10^,[13], [14], [15], [16]

Rewards: oils	Describes whether the plant provides oils.	Absent Present	[4,^5^,^10^,[13], [14], [15], [16]

Rewards: pollen	Describes whether the plant provides pollen resources.	Absent Present	[4,^5^,^10^,[13], [14], [15], [16]

Structural Blossom Class	Describing the shape of the blossom of the plant species.	Dish-bowl Stalk-disk Bell trumpet Brush Gullet Flag Tube	Adapted from [17]

Symmetry	Describes the number of axes of reflection of a flower of a plant species. The value was derived from the structural blossom class	No symmetry Zygomorph Actinomorph

**Table 3:** Summary statistics of the plants recorded. For each of the 80 study sites in the five cities, we provide the number of species (**N_species_**), genera (**N_genera_**), families (**N_families_**), the value of the Shannon diversity index (**H’**), and the proportion of herbs (**P_herbs_**), shrubs (**P_shrubs_**), trees (**P_trees_**), and exotic species (**P_exotic_**). H’ was calculated using the frequency of each plant species, obtained as the number of cells from the total 16 where the plant was found. The data are based on floristic inventories in the study sites. The data are plotted in Fig. 1-2. Site codes represent the study sites shown in Fig. 1. Note that the statistic does not include species in the families Cyperaceae, Juncaceae and Poaceae. The coordinates of the sites are provided in Table 4.

City	Site	N_species_	N_genera_	N_families_	H'	P_herbs_	P_shrubs_	P_trees_	P_exotic_
*Antwerp*	An011	91	87	41	4.75	0.72	0.13	0.13	0.34
	An016	44	39	22	3.83	0.74	0.17	0.06	0.13
	An020	61	57	33	4.3	0.7	0.16	0.12	0.27
	An056	52	44	26	4.01	0.86	0.09	0.04	0.15
	An057	27	24	15	3.3	0.7	0.11	0.15	0.3
	An062	65	53	25	4.16	0.79	0.06	0.09	0.36
	An068	60	60	36	4.2	0.61	0.16	0.16	0.48
	An073	64	52	29	4.26	0.66	0.17	0.14	0.31
	An082	61	56	29	4.16	0.64	0.17	0.14	0.39
	An088	47	39	24	3.89	0.65	0.2	0.08	0.33
	An092	53	45	24	4.03	0.77	0.11	0.09	0.23
	An102	85	73	36	4.55	0.77	0.13	0.08	0.32

*Paris*	Pa013	191	138	51	5.3	0.72	0.18	0.08	0.33
	Pa191	148	124	51	5.11	0.7	0.17	0.11	0.44
	Pa245	102	90	40	4.71	0.73	0.14	0.11	0.21
	Pa265	91	79	39	4.62	0.71	0.19	0.09	0.38
	Pa269	171	146	56	5.19	0.65	0.22	0.11	0.36
	Pa282	83	68	34	4.6	0.7	0.07	0.21	0.22
	Pa295	125	112	54	4.95	0.69	0.19	0.1	0.43
	Pa398	1167	555	100	7.07	0.83	0.13	0.02	0.42
	Pa418	85	75	36	4.48	0.76	0.16	0.05	0.41
	Pa492	91	74	36	4.58	0.82	0.09	0.09	0.2
	Pa535	122	110	46	4.91	0.77	0.11	0.1	0.39
	Pa573	52	51	32	4.08	0.5	0.29	0.17	0.39

*Poznan*	Po001	45	43	19	3.91	0.84	0.12	0.04	0.28
	Po037	12	24	16	3.26	0.62	0.15	0.23	0.35
	Po059	56	56	28	4.13	0.76	0.13	0.11	0.24
	Po137	37	29	14	3.64	0.84	0.13	0.03	0.24
	Po179	36	32	19	3.66	0.77	0.05	0.16	0.18
	Po183	75	67	28	4.41	0.74	0.15	0.09	0.28
	Po210	35	33	18	3.69	0.92	0.03	0.05	0.12
	Po227	58	65	32	4.28	0.72	0.1	0.16	0.38
	Po267	38	42	23	3.91	0.8	0.02	0.18	0.16
	Po348	63	52	24	4.2	0.72	0.15	0.11	0.3
	Po406	44	42	18	3.89	0.84	0.06	0.06	0.24
	Po423	72	66	31	4.39	0.79	0.11	0.1	0.2

*Tartu*	Ta008	87	73	31	4.53	0.89	0.06	0.03	0.29
	Ta013	59	48	24	4.14	0.87	0.02	0.08	0.11
	Ta025	51	45	21	3.95	0.92	0.02	0.06	0.08
	Ta033	48	40	17	3.85	0.98	0	0.02	0.11
	Ta040	100	86	35	4.63	0.89	0.04	0.06	0.24
	Ta047	64	57	29	4.22	0.85	0.03	0.07	0.1
	Ta057	79	66	29	4.48	0.91	0.02	0.05	0.22
	Ta064	41	38	20	3.81	0.89	0.02	0.09	0.09
	Ta102	46	43	18	3.95	0.94	0.02	0.04	0.06
	Ta104	51	43	19	3.99	0.91	0.04	0.06	0.07
	Ta110	78	63	28	4.37	0.92	0.05	0.02	0.19
	Ta125	59	60	30	4.29	0.87	0.03	0.07	0.21

*Zurich*	Zu006	210	143	57	5.39	0.8	0.12	0.06	0.34
	Zu007	131	100	35	4.91	0.9	0.04	0.05	0.24
	Zu015	730	386	99	6.6	0.83	0.11	0.05	0.41
	Zu018	210	142	53	5.42	0.74	0.13	0.1	0.3
	Zu033	279	187	58	5.68	0.75	0.12	0.1	0.33
	Zu039	144	115	45	5.04	0.81	0.09	0.08	0.24
	Zu057	261	187	62	5.65	0.67	0.15	0.15	0.32
	Zu062	144	115	47	4.99	0.74	0.16	0.1	0.32
	Zu067	168	128	50	5.21	0.78	0.1	0.07	0.33
	Zu080	110	88	40	4.73	0.82	0.09	0.09	0.15
	Zu082	212	158	56	5.42	0.77	0.09	0.11	0.31
	Zu087	106	85	32	4.71	0.77	0.13	0.07	0.25
	Zu094	254	185	62	5.6	0.84	0.08	0.05	0.28
	Zu105	126	86	32	4.87	0.79	0.1	0.1	0.1
	Zu113	158	109	44	5.11	0.75	0.15	0.09	0.19
	Zu119	136	95	36	4.93	0.85	0.05	0.1	0.12
	Zu126	223	171	56	5.48	0.81	0.12	0.06	0.33
	Zu133	238	162	57	5.51	0.76	0.15	0.07	0.33
	Zu141	112	114	41	5.21	0.79	0.12	0.08	0.21
	Zu154	201	136	48	5.3	0.81	0.1	0.06	0.25
	Zu155	245	81	27	4.8	0.87	0.07	0.05	0.12
	Zu158	149	132	45	5.34	0.85	0.06	0.06	0.21
	Zu173	191	168	51	5.55	0.79	0.12	0.07	0.32
	Zu179	180	110	45	5.06	0.83	0.13	0.02	0.28
	Zu904	172	131	45	5.19	0.88	0.04	0.06	0.27
	Zu905	161	124	46	5.1	0.83	0.09	0.06	0.26
	Zu906	237	156	53	5.49	0.87	0.07	0.04	0.29
	Zu907	205	146	46	5.35	0.79	0.13	0.001	0.28
	Zu908	182	122	51	5.26	0.74	0.13	0.11	0.27
	Zu910	220	159	57	5.47	0.74	0.12	0.09	0.28
	Zu911	213	136	51	5.4	0.83	0.09	0.06	0.22
	Zu912	113	86	41	4.76	0.67	0.16	0.14	0.25

We provide information on the study site features including the city, their size, connectivity, and urban intensity inferred using a set of remote sensing-based proxies on soil, grey infrastructure, and vegetation, including the Second Brightness Index (BI2), the Color Index (CI), the Urban Index (UI), and the Normalized Difference Vegetation Index (NDVI) within a 100 and 800 m buffer centered in the centroid of the urban green area (Table 4). The data are part of the interdisciplinary research project BioVeins investigating different aspects of urban biodiversity and ecosystem services in urban green areas in European cities (https://www.biodiversa.org/1012). The data can be linked to other taxonomic groups such as nocturnal insects and bats [2], sampled in the same study locations and during the same period. The raw data are available from the repository Envidat [3] with the DOI doi:10.16904/envidat.210.

**Table 4:** Site features for each study site based on remote sensing data. For each of the 80 study sites in the five cities, we provide the coordinates where the trap-nest was located, the proximity index (Prox), and patch area (Area) used to select the study sites, and the values of the Second Brightness Index (BI2), Color Index (CI), Urban Index (UI), and Normalized Difference Vegetation Index (NDVI) at 100- and 800-meter radii.

City	Site	X	Y	Prox.	Area (m^2^)	BI2_100_	BI2_800_	CI_100_	CI_800_	UI_100_	UI_800_	NDVI_100_	NDVI_800_
*Antwerp*	An011	4.36	51.16	1218.96	1,085,854	0.18	0.18	−0.12	−0.10	−0.38	−0.33	0.67	0.55
	An016	4.42	51.18	931.47	12,426	0.16	0.15	−0.13	−0.07	−0.42	−0.31	0.68	0.54
	An020	4.37	51.18	6.82	20,169	0.15	0.13	−0.06	0.02	−0.28	−0.09	0.49	0.30
	An056	4.48	51.21	247.14	1,054,885	0.20	0.16	−0.21	−0.11	−0.52	−0.34	0.78	0.58
	An057	4.39	51.21	1.52	6704	0.13	0.10	0.03	0.02	−0.03	−0.10	0.25	0.05
	An062	4.44	51.22	3.31	11,116	0.14	0.11	0.05	0.04	−0.09	−0.03	0.31	0.22
	An068	4.42	51.22	2.31	93,542	0.14	0.11	−0.08	0.02	−0.29	−0.02	0.46	0.20
	An073	4.39	51.22	49.92	56,928	0.19	0.13	−0.09	0.00	−0.34	−0.18	0.57	0.14
	An082	4.47	51.24	4.48	60,943	0.17	0.15	−0.19	−0.02	−0.50	−0.14	0.76	0.33
	An088	4.46	51.25	7.69	14,401	0.14	0.15	−0.04	−0.05	−0.26	−0.25	0.54	0.47
	An092	4.45	51.26	91.92	56,166	0.18	0.17	−0.06	−0.07	−0.36	−0.31	0.62	0.54
	An102	4.43	51.29	3995.62	52,059	0.16	0.17	0.03	−0.03	−0.20	−0.24	0.48	0.50

*Paris*	Pa013	2.17	48.70	24.13	126,628	0.19	0.18	−0.14	−0.14	−0.45	−0.41	0.66	0.61
	Pa191	2.30	48.80	29.42	24,993	0.17	0.16	−0.16	−0.01	−0.46	−0.16	0.67	0.35
	Pa245	2.42	48.84	2792.45	5,933,064	0.18	0.17	−0.10	−0.07	−0.48	−0.29	0.69	0.46
	Pa265	2.37	48.83	2.00	3553	0.15	0.14	0.03	0.02	−0.01	−0.06	0.22	0.23
	Pa269	2.34	48.82	5.39	159,611	0.16	0.15	−0.11	0.00	−0.42	−0.13	0.62	0.30
	Pa282	2.38	48.83	3.80	9890	0.15	0.14	−0.02	−0.01	−0.10	0.02	0.27	0.10
	Pa295	2.37	48.83	2.01	8339	0.15	0.14	−0.01	0.01	−0.11	0.00	0.28	0.14
	Pa398	2.36	48.84	2.98	169,327	0.21	0.14	−0.11	−0.03	−0.47	−0.04	0.65	0.14
	Pa418	2.29	48.84	9.83	4630	0.14	0.13	0.01	0.01	0.01	−0.01	0.14	0.14
	Pa492	2.26	48.85	45,794.28	9148	0.15	0.15	−0.07	−0.04	−0.29	−0.21	0.46	0.35
	Pa535	2.32	48.87	49.76	164,101	0.18	0.14	−0.07	−0.04	−0.34	−0.03	0.49	0.11
	Pa573	2.32	48.88	1.79	4607	0.13	0.13	−0.01	−0.01	0.04	0.07	0.09	−0.01
*Poznan*	Po001	16.98	52.31	862.43	30,443	0.16	0.17	−0.06	−0.10	−0.29	−0.34	0.56	0.57
	Po037	16.90	52.36	11.66	48,772	0.17	0.15	−0.22	−0.08	−0.50	−0.30	0.69	0.48
	Po059	16.88	52.37	5.96	8200	0.13	0.14	−0.03	−0.04	−0.22	−0.24	0.44	0.40
	Po137	16.93	52.39	31.09	187,103	0.17	0.15	−0.13	−0.07	−0.44	−0.28	0.68	0.47
	Po179	16.90	52.40	3.46	56,886	0.17	0.13	−0.17	0.01	−0.45	−0.09	0.66	0.24
	Po183	16.95	52.40	2136.45	10,423	0.14	0.15	−0.05	−0.04	−0.30	−0.19	0.49	0.35
	Po210	16.93	52.41	7.95	13,222	0.15	0.12	−0.08	0.01	−0.24	−0.06	0.44	0.21
	Po227	16.87	52.41	10.50	8406	0.14	0.15	−0.08	−0.06	−0.33	−0.28	0.53	0.46
	Po267	16.95	52.43	325.97	1,059,825	0.17	0.16	−0.16	−0.11	−0.44	−0.35	0.68	0.53
	Po348	16.93	52.44	18.63	18,721	0.16	0.15	−0.09	−0.07	−0.37	−0.29	0.57	0.47
	Po406	16.92	52.46	468.47	5624	0.15	0.14	−0.04	−0.02	−0.23	−0.19	0.45	0.40
	Po423	16.93	52.47	12,829.47	27,974	0.14	0.15	−0.11	−0.12	−0.32	−0.36	0.56	0.56

*Tartu*	Ta008	26.77	58.35	14.27	6338	0.16	0.16	−0.17	−0.15	−0.40	−0.37	0.66	0.61
	Ta013	26.74	58.35	2.74	122,857	0.17	0.16	−0.23	−0.05	−0.41	−0.16	0.68	0.34
	Ta025	26.70	58.37	2.87	33,237	0.15	0.15	−0.17	−0.08	−0.36	−0.21	0.60	0.44
	Ta033	26.68	58.38	5.78	6225	0.14	0.16	−0.05	−0.06	−0.18	−0.20	0.40	0.42
	Ta040	26.73	58.37	314.56	36,590	0.15	0.14	−0.08	−0.07	−0.22	−0.16	0.43	0.35
	Ta047	26.72	58.38	57.84	131,100	0.14	0.14	−0.23	−0.10	−0.38	−0.21	0.65	0.43
	Ta057	26.69	58.38	5.36	5066	0.16	0.16	−0.11	−0.08	−0.28	−0.22	0.52	0.44
	Ta064	26.74	58.37	14.97	183,227	0.16	0.14	−0.22	−0.10	−0.42	−0.22	0.56	0.39
	Ta102	26.70	58.39	22.54	13,236	0.15	0.16	−0.18	−0.14	−0.38	−0.32	0.64	0.56
	Ta104	26.76	58.38	5.32	37,412	0.18	0.17	−0.21	−0.11	−0.41	−0.26	0.67	0.50
	Ta110	26.73	58.39	7.02	8623	0.15	0.15	−0.11	−0.09	−0.27	−0.24	0.53	0.45
	Ta125	26.73	58.39	26.38	245,706	0.15	0.15	−0.26	−0.12	−0.45	−0.29	0.73	0.53

*Zurich*	Zu006	8.52	47.35	104.93	104,871	0.17	0.16	−0.19	−0.12	−0.49	−0.32	0.77	0.59
	Zu007	8.56	47.35	7.01	3717	0.08	0.10	−0.24	−0.28	−0.02	−0.11	0.08	0.16
	Zu015	8.56	47.36	167.23	39,258	0.17	0.14	−0.20	−0.06	−0.46	−0.20	0.74	0.51
	Zu018	8.53	47.36	56.97	57,666	0.17	0.13	−0.13	−0.11	−0.39	−0.14	0.68	0.36
	Zu033	8.56	47.36	28.24	10,400	0.12	0.14	−0.04	−0.07	−0.15	−0.21	0.50	0.52
	Zu039	8.54	47.36	10.96	36,883	0.15	0.10	−0.12	−0.18	−0.32	0.01	0.52	0.12
	Zu057	8.53	47.37	6.74	13,040	0.13	0.11	−0.14	−0.01	−0.29	0.01	0.57	0.25
	Zu062	8.54	47.37	6.16	18,037	0.12	0.11	−0.03	−0.02	−0.06	0.04	0.39	0.22
	Zu067	8.51	47.37	14.78	275,320	0.18	0.14	−0.18	−0.05	−0.48	−0.17	0.75	0.49
	Zu080	8.54	47.38	8.75	26,855	0.14	0.11	−0.28	−0.02	−0.38	−0.02	0.64	0.27
	Zu082	8.49	47.38	17.51	13,854	0.16	0.15	−0.13	−0.06	−0.34	−0.18	0.68	0.49
	Zu087	8.52	47.39	4.87	22,711	0.13	0.12	−0.02	0.01	−0.13	0.00	0.34	0.21
	Zu094	8.47	47.39	974.64	96,182	0.20	0.17	−0.17	−0.17	−0.42	−0.40	0.69	0.67
	Zu105	8.50	47.40	67.97	9576	0.16	0.14	−0.21	−0.05	−0.36	−0.16	0.67	0.39
	Zu113	8.52	47.40	34,334.06	46,486	0.18	0.15	−0.11	−0.15	−0.27	−0.34	0.57	0.62
	Zu119	8.54	47.40	25.45	108,059	0.16	0.14	−0.15	−0.05	−0.39	−0.19	0.63	0.49
	Zu126	8.50	47.40	15.67	11,748	0.17	0.16	−0.10	−0.09	−0.31	−0.26	0.61	0.55
	Zu133	8.54	47.41	13.91	3511	0.14	0.14	−0.06	−0.04	−0.21	−0.16	0.52	0.45
	Zu141	8.48	47.41	32.05	8421	0.15	0.16	−0.07	−0.12	−0.26	−0.32	0.53	0.58
	Zu154	8.51	47.41	750.61	57,150	0.17	0.16	−0.07	−0.17	−0.32	−0.38	0.56	0.65
	Zu155	8.55	47.41	6.51	4346	0.17	0.14	−0.03	−0.01	−0.17	−0.08	0.33	0.35
	Zu158	8.53	47.41	7.75	5936	0.12	0.15	0.00	−0.02	−0.07	−0.13	0.35	0.41
	Zu173	8.51	47.42	25.03	5607	0.13	0.16	−0.09	−0.11	−0.26	−0.30	0.56	0.59
	Zu179	8.53	47.42	2778.23	103,083	0.19	0.17	−0.21	−0.11	−0.46	−0.30	0.76	0.57
	Zu904	8.52	47.39	5.04	8253	0.13	0.13	0.01	0.02	−0.09	0.01	0.28	0.18
	Zu905	8.56	47.41	7.02	10,987	0.14	0.15	−0.03	−0.02	−0.14	−0.11	0.43	0.37
	Zu906	8.59	47.40	9.10	10,629	0.15	0.15	−0.05	−0.07	−0.22	−0.20	0.53	0.48
	Zu907	8.49	47.40	25.21	22,894	0.15	0.14	−0.08	−0.06	−0.24	−0.17	0.53	0.40
	Zu908	8.58	47.35	262.43	102,401	0.17	0.16	−0.25	−0.16	−0.54	−0.38	0.81	0.65
	Zu910	8.53	47.34	14.50	53,898	0.17	0.13	−0.12	−0.14	−0.30	−0.13	0.63	0.34
	Zu911	8.50	47.43	18.09	3219	0.15	0.17	−0.05	−0.09	−0.20	−0.28	0.43	0.53
	Zu912	8.55	47.35	8.71	89,860	0.16	0.08	−0.13	−0.30	−0.31	−0.08	0.47	0.09

## 2 Experimental Design, Materials and Methods

### 2.1 Data source

The data was acquired in the European cities of Antwerp, Belgium (51°15′N, 4°24′E), Greater Paris, France (48°51′N, 8°05′E), Poznan, Poland (52°24′N, 16°55′E), Tartu, Estonia (58°22′N, 26°43′E), Zurich, Switzerland (47°22′N, 8°33′E). The climate of Antwerp is oceanic, the climate of Paris is temperate, the climate of Poznan is continental, the climate of Tartu is mild continental boreal and the climate of Zurich is mild continental temperate. The agglomeration of greater Paris is the most populated one in Europe with more than seven million inhabitants (2.18 million inhabitants only in the city of Paris [19]). Antwerp has the second highest population (0.53 million inhabitants [19]) followed by Poznan (0.53 million inhabitants [19]), Zurich (0.4 million inhabitants [19]), and Tartu (0.09 million inhabitants [19]).

### 2.2 Site selection

We selected patches among urban green areas mapped and defined in the European Urban Atlas [see 20], which includes mostly public urban green areas in the form of parks, cemeteries, and ruderal patches. We used an orthogonal gradient of patch size (area in m2) and connectivity. Connectivity was calculated using the Proximity Index (PI) which considers the area and the distance to all nearby patches with a favorable habitat, within a given search radius (in our case 5000 m), and is defined as:

$$PI=\sum_{s=1}^{n}\frac{a_{ijs}}{h_{ijs}^{2}}$$

Where *a_ijs_* is the area (m^2^) of a patch *ijs* within specified neighbourhood (m) of a patch *ij*, and $h_{ijs}^{2}$ is the distance (m) between the patch *ijs*, based on patch edge-to-edge distance.

Thus, the PI measures the degree of patch isolation, with highest values given to less isolated patches. We considered as favourable habitat all patches with high probability of having trees (besides urban green areas, also forest and low density urban, with less than 30% impervious surface, see [20]). The search radius was set to 5 km from each focal patch, the maximum possible with the available cartography. In fact, lower buffer values (from 500 m onwards) did not greatly change the PI values, because the distances are squared, thus greatly limiting the impact of patches beyond a certain distance. To select patches using the orthogonal design, all possible patches were classified in six size classes and six classes of the PI (36 possible combinations). Within these combinations, patches were selected randomly (random stratified sampling design). Due to resource limitations, we only used 1⁄3 of the possible combinations in Antwerp, Paris, Poznan, and Tartu (maximizing the gradient) and the full range of combinations in Zurich (32 combinations, the other combinations were not available in the city). This resulted in the final selection of 80 sites (Fig. 1): 32 in Zurich and 12 in each of the remaining cities. Sites were selected keeping a minimum distance of 500 m (except for two sites in Zurich selected by their position in the patch and connectivity gradient, separated by 260 m). Median distance to the nearest site was 6610 m in Antwerp (minimum = 966 m, maximum = 15,375 m), 7852 m in Paris (minimum = 721 m, maximum = 31,891 m), 3912 m in Poznan (minimum = 1630 m, maximum = 17,189 m), 3913 m in Tartu (minimum = 788 m, maximum = 10,520 m), and 4299 m in Zurich (minimum = 371 m, maximum = 10,560 m). Furthermore, pairwise distances among sites were in 99% of the cases larger than 750 m.

### 2.3 Remote sensing indices

Urban intensity has been inferred using remote sensing indices on soil, impervious surfaces and vegetation. Particularly, we used the BI2, CI, UI, and NDVI. The BI2 results from the following equation:

BI2=√(((ρRED*ρRED)+(ρGREEN*ρGREEN)+(ρNIR*ρNIR))/3)

Where ρRED, ρGREEN and ρNIR are the responses in red, green, and near-infrared bands, respectively. This index is sensitive to the brightness of soils, which in turn is influenced by soil moisture, presence of salts and organic matter content on the soil surface. Thus, brightness values greater than 0.3 are an indicator of soil problems with less decomposed organic materials, which can be reflected in a lower development of trees. In turn, low values of brightness are associated with soils with high moisture content and decomposed organic materials, favoring the growth of tree plants.

The Colour Index (CI) was introduced by Pouget et al. [21] and results from the following equation:

CI=(ρRED−ρGREEN)/(ρRED+ρGREEN)

Where ρRED and ρGREEN are the responses in the red and green bands, respectively. Although this index was developed to differentiate various types of soils in arid environments, it can help to compute better vegetation indices for incomplete canopies. In most cases, the CI provides complementary information with the BI2 and the NDVI, allowing to differentiate plants and soil more effectively, especially in study areas with less than 10% vegetation [21]. Typically, low CI values have been shown to be correlated with the presence of a high concentration of carbonates or sulfates, nutrients that can serve as fertilizers for plant growth. Meanwhile, higher values have been correlated with crusty and sandy soils and with a low content of organic matter. Thus, this index seems to be a good indicator of soil degradation.

The Urban Index (UI) was developed by Kawamura et al. [22] to effectively detect the structural details of urban cores. The UI was calculated using the following equation:

UI=(ρSWIR2−ρNIR)/(ρSWIR2+ρNIR).

Where ρMIR2 and ρNIR are the responses in the second short wave and near-infrared bands, respectively. Thus, it is a good index for detecting built and non-built areas and can also be used to identify building densities. The built-up area tends to have UI values greater than 0, while negative values close to −1 tend to be green areas.

Finally, the NDVI was developed by Tucker [23] and is the one of the most common indices widely applied for monitoring vegetation dynamics. This index results from the following equation:

NDVI=(ρNIR−ρRED)/(ρNIR+ρRED)

Where ρNIR and ρRED are the responses in near infrared and red bands, respectively. This index indicates the photosynthetic capacity, or the energy absorbed by plant canopies, hence, the amount of healthy vegetation. Thus, higher NDVI values indicate a higher density of green vegetation. Specifically, in urban environments, NDVI values greater than 0.5 correspond to vigorous green areas, while NDVI values between 0.2 to 0.5 indicate moisture-stressed vegetation, such as natural meadows. NDVI values near zero and decreasing negative values indicate non-vegetated features, such as artificial and barren surfaces, water bodies, snow, and clouds.

These four indices can be used to charecterize the existing vegetation and urban infrastructure in the vicinity of sampling sites. Remotely sensed data can be used to improve research on biodiversity and ecosystem services, being a valuable tool to support more sustainable urban planning and management.

### 2.4 Floristic inventories

Between April and July 2018, we sampled all available plants of potential interest for pollinators (i.e. we excluded the families Cyperaceae, Juncaceae and Poaceae) in a buffer of 100 m radius around each trap nest on green areas both public and private within the defined radius in three sampling periods. Each buffer was divided in 16 cells (see Fig. 1). In each buffer, we documented all the plant species found, in order to obtain an estimate of both plant richness and frequency (as the number of cells inside a buffer each species was found). To identify plant species, we used identification guides for European [14,^15^,24] and Swiss flora [25], as well as specialized guides for ornamental plants [13,^16^,26] and previous species inventories, e.g. [9] in Zurich. The total duration of each sampling in a site was restricted to about 2.5 h to standardize sampling effort. Note that the late winter and early spring flowers were missed (e.g. *Crocus* spp., *Galanthus* spp.). The species, genus, and family richness of each sampling site are given in Table 3. The list of all taxa and the number of observations per taxon are given in the Supplementary material, Table A1.

### 2.5 Floral counts on standardized plots

We calculated the floral abundance in a site and sampling period by using 1 m^2^ quadrats randomly distributed inside the 100 m buffer. The number of quadrats was determined according to the amount of green areas in each buffer, with a minimum of seven quadrats, when less than 20% of the buffer was covered by green areas, and a maximum of 15 quadrats, when more than 90%. To obtain the floral abundance, we first defined a set of floral units on where we classified the different plant species (see Table 1). The floral abundance of each floral unit type was calculated in the following way. For single flowers, the floral abundance was obtained by summing all the individual flowers (Table 1). For single capitula (in Dipsacoideae species), single compound cymes, single corymbs, single panicles, single racemes, single umbels, we took seven different floral units, counted all the flowers and computed a mean number of flowers per floral unit. The mean number of flowers per floral unit was calculated separately for each site and sampling period. The floral abundance was then obtained by multiplying the number of floral units and the mean number of flowers per floral unit (see Table 1). Finally, for single capitula (in Asteraceae), single catkins, single corymbs or cymes in the *Euphorbia* genus, single dense clusters (including only *Sangisorba* spp.) and single spikes (including the genus *Plantago* spp. and *Tamarix* spp.) the floral abundance was computed by summing only the number of floral units, that is, we did not estimate a mean number of flowers per floral unit.

### 2.6 Taxonomic treatment

Taxonomy assignment largely followed the criteria of Checklist of the National Data and Information Centre of the Swiss Flora [27], together with The World Flora Online database [28], and other resources, e.g. RHS Dictionary of Gardening [16]. Varieties, taxa within species complexes, and cultivars were mostly grouped into aggregates (e.g. Taraxacum officinale aggr.) or left at the genus level (e.g. *Leucanthemum* sp.) without further distinction.

### 2.7 Traits

We aimed to select important determinants of plant-pollinator interactions. We developed a data set of 11 traits (see Table 2) for 2313 plant species. We used 11 functional traits including start and duration of the flowering period, growth type, inflorescence type, pollination mode, blossom class, symmetry, plant height and the presence of rewards in the form of pollen, nectar and oils. Additionally, we included the origin of the plant species, which are no functional traits *per se.* For functional traits, we used as main sources the TRY plant trait database [10], the national data, and information centre for the Swiss flora [6], the Bundesamt für Naturschutz (BfN) [7], Faegri and van der Pijl [17], Frey and Moretti [9], Missouri Botanical Garden Plant Finder [5], Plants For A Future [4], Plants of the World Online [12], and BiolFlor [8]. Regarding the origin status of the plant species, a species was considered to be native when its origin was Europe and exotic if it originated elsewhere. To document the origin status of each plant species, we used the Global Biodiversity Information System [29]. Cultivar groups not derived from native plants were considered to be alien.

The start and duration of the flowering period are given in months. For exotic plants from the Southern hemisphere, we do not provide information on the phenology. We defined the pollination mode for each species, based on Frey and Moretti [9]. Here, we distinguished whether a species is biotically or abiotically pollinated, i.e., mainly either by insects (entomophilous) or by wind (anemophilous). Concerning growth form, we defined four broad categories, that is, tree, shrub, herb, and climber. Trees included woody species typically classified as phanaerophytes, including species described as small trees or tall shrubs (e.g. *Crataegus* spp., *Ligustrum* spp.). Shrubs included mostly chamaephytes. Herbs included all herbaceous plants regardless of their height or growth form. Finally, climbers included woody and non-woody epyphites such as lianas and vines.

The inflorescence types considered are the same as the ones in the floral counts (see Supplementary material, Table A1). We considered the type of structural blossom classes according to Faegri and van der Pijl [17]. Concerning symmetry, each plant was classified in three main categories of actinomorphy (two or more axis of symmetry), zygomorphy (one axis of symmetry) or without symmetry. Finally, for the rewards, we reported whether the plant species had been shown to provide floral resources in the form of nectar, oil and pollen.

## CRediT Author Statement

**Joan Casanelles-Abella:** Conceptualisation, Methodology, Investigation, Validation, Data curation, Writing Original draft, Writing Review & Editing, Formal analysis, Project administration; **David Frey:** Methodology, Validation, Resources, Data curation, Writing Review & Editing; **Stefanie Müller:** Conceptualisation, Methodology, Investigation, Data curation, Writing Review & Editing; **Cristiana Aleixo:** Investigation, Data curation, Resources, Writing Review & Editing; **Marta Alós Ortí:** Investigation, Writing Review & Editing; **Nicolas Deguines:** Investigation, Validation, Writing Review & Editing; **Tiit Hallikma:** Investigation, Validation, Writing Review & Editing; **Lauri Laanisto:** Methodology, Writing Review & Editing; **Ülo Niinemets:** Funding acquisition, Writing Review & Editing; **Pedro Pinho:** Funding acquisition, Methodology, Investigation, Data curation, Resources, Writing Review & Editing; **Roeland Samson:** Funding acquisition, Methodology; **Lucía Villarroya-Villalba:** Investigation, Validation, Writing Review & Editing; **Marco Moretti:** Funding acquisition, Conceptualisation, Methodology, Writing Review & Editing, Supervision, Project administration.

## Declaration of Competing Interest

The authors declare that they have no known competing financial interests or personal relationships which have or could be perceived to have influenced the work reported in this article.

## Appendix Supplementary materials

Image, application 1

## Footnotes

Supplementary material associated with this article can be found in the online version at doi:10.1016/j.dib.2021.107243.
