# Supplementary material for: A dataset of the flowering plants (Angiospermae) in urban green areas in five European cities
Source: Data Brief. 2021 Jun 25;37:107243. doi: 10.1016/j.dib.2021.107243 (PMC8258796; doi:10.1016/j.dib.2021.107243)
Supplement: Supplementary file 1 [file mmc1.pdf]

Supplementary material for 'A Dataset of the Flowering Plants (Angiospermae) in Urban Green Areas in five European Cities'

Casanelles-Abella, J.; Frey, D. ; Müller, S. ; Aleixo, C.; Alós Ortí, M.; Deguines, N.; Hallikma, T.;

Laanisto, L.; Niinemets, Ü.; Pinho, P.; Samson, R.; Villarroya-Villalba, L.; Moretti, M.

Appendix A

Table SA. List of all the plant species recorded in the 80 sampling sites in the five European cities, that is, Antwerp (An, 12), Paris (Pa, 12), Poznan (Po, 12), Tartu (Ta, 12), and Zurich (Zu, 32). For every species, the number of sites in a city where the species occurred is provided. Note that the study sites included botanical gardens (i.e. one in Paris and two in Zürich), which contained a large number of rare species hardly found in nurseries or gardens. CV = cultivated species

Family	Species		Authority	An	Pa	Po	Ta	Zu
Caprifoliaceae	*Abelia*	*chinensis*	R.Br.	0	1	0	0	0
Caprifoliaceae	*Abelia*	*parvifolia*	Hemsl.	0	1	0	0	0
Caprifoliaceae	*Abelia*	sp.		0	0	0	0	2
Caprifoliaceae	*Abelia*	*triflora*	R.Br. ex Wall.	0	1	0	0	0
Caprifoliaceae	*Abelia*	*× grandiflora*	(Ravelli ex André) Rehder	0	7	0	0	1
Malvaceae	*Abelmoschus*	*esculentus*	(L.) Moench	0	0	0	0	1
Malvaceae	*Abelmoschus*	*moschatus*	Medik.	0	1	0	0	0
Malvaceae	*Abutilon*	*megapotamicum*	Naudin	0	1	0	0	0
Malvaceae	*Abutilon*	*theophrasti*	Medik.	0	0	0	0	1
Fabaceae	*Acacia*	*melanoxylon*	R. Br.	0	0	0	0	1
Acanthaceae	*Acanthus*	*hungaricus*	(Borbás) Baen.	0	0	0	0	3
Acanthaceae	*Acanthus*	*mollis*	L.	0	3	0	0	0
Acanthaceae	*Acanthus*	*spinosus*	L.	0	1	0	0	0
Myrtaceae	*Acca*	*sellowiana*	(O. Berg) Burret	0	1	0	0	0
Sapindaceae	*Acer*	*campestre*	L.	0	0	0	0	5
Sapindaceae	*Acer*	*platanoides*	L.	0	0	0	1	13

Sapindaceae	*Acer*	*pseudoplatanus*	L.	1	0	0	2	9
Sapindaceae	*Acer*	*tataricum*	L.	0	0	0	0	1
Asteraceae	*Achillea*	*ageratum*	L.	0	1	0	0	0
Asteraceae	*Achillea*	*alpina*	L.	0	1	0	0	0
Asteraceae	*Achillea*	*atrata*	L.	0	0	0	0	4
Asteraceae	*Achillea*	*clypeolata*	Sm.	0	1	0	0	0
Asteraceae	*Achillea*	*filipendulina*	Lam.	0	1	0	0	4
Asteraceae	*Achillea*	*ligustica*	All.	0	1	0	0	0
Asteraceae	*Achillea*	*millefolium aggr.*		7	7	11	12	27
Asteraceae	*Achillea*	*odorata*	L.	0	1	0	0	0
Asteraceae	*Achillea*	*ptarmica*	L.	0	1	1	1	5
Asteraceae	*Achillea*	*ptarmicifolia*	(Willd.) Rupr. ex Heimerl	0	1	0	0	0
Asteraceae	*Achillea*	*tomentosa*	L.	0	1	0	0	0
Lamiaceae	*Acinos*	*arvensis*	(Lam.) Dandy	0	1	2	0	1
Ranunculaceae	*Aconitum*	*ferox Wall.*	ex Ser.	0	1	0	0	0
Ranunculaceae	*Aconitum*	*lycoctonum*	L.	0	1	0	0	0
Ranunculaceae	*Aconitum*	*napellus*	L.	0	2	0	0	2
Ranunculaceae	*Aconitum*	*lycoctonum*	L.	0	0	0	0	1
Ranunculaceae	*Aconitum*	*lycoctonum subsp. neapolitanum*	(Ten.) Nyman	0	0	0	0	1
Campanulaceae	*Adenophora*	*liliifolia*	(L.) A. DC.	0	0	0	0	2
Asteraceae	*Adenostyles*	*glabra*	(Mill.) DC.	0	0	0	0	1
Ranunculaceae	*Adonis*	*aestivalis*	L.	0	0	0	0	3
Ranunculaceae	*Adonis*	*amurensis*	Regel & Radde	0	0	0	0	1
Ranunculaceae	*Adonis*	*annua*	L.	0	1	0	0	0

Ranunculaceae	*Adonis*	*vernalis*	L.	0	0	0	0	2
Lamiaceae	*Aegiphila*	*bogotensis*	(Spreng.) Moldenke	0	0	0	1	0
Apiaceae	*Aegopodium*	*podagraria*	L.	3	1	2	12	8
Orchidaceae	*Aerobium*	sp.		0	1	0	0	0
Sapindaceae	*Aesculus*	*hippocastanum*	L.	3	6	0	0	6
Sapindaceae	*Aesculus*	*parviflora*	Walter	0	2	0	0	0
Sapindaceae	*Aesculus*	*pavia*	L.	0	0	0	0	1
Sapindaceae	*Aesculus*	*× carnea*	Zeyh.	1	0	1	0	8
Brassicaceae	*Aethionema*	*grandiflorum*	Boiss. & Hohen.	0	2	0	0	0
Apiaceae	*Aethusa*	*cynapium*	L.	0	0	0	3	0
Amaryllidaceae	*Agapanthus*	CV		0	4	0	0	2
Lamiaceae	*Agastache*	*rupestris*	(Greene) Standl.	0	1	0	0	0
Asparagaceae	*Agave*	*parryi*	Engelm.	0	1	0	0	0
Asteraceae	*Ageratina*	*altissima*	(L.) R. M. King & H. Rob.	0	0	0	0	1
Asteraceae	*Ageratum*	*houstonianum*	Mill.	1	4	0	0	10
Rosaceae	*Agrimonia*	*eupatoria*	L.	0	1	0	0	7
Rosaceae	*Agrimonia*	*procera*	Wallr.	0	1	0	0	0
Caryophyllaceae	*Agrostemma*	*githago*	L.	0	1	0	0	1
Simaroubaceae	*Ailanthus*	*altissima*	(Mill.) Swingle	0	0	0	0	1
Lamiaceae	*Ajuga*	*genevensis*	L.	0	0	0	0	1
Lamiaceae	*Ajuga*	*reptans*	L.	1	9	0	3	25
Malvaceae	*Alcea*	*rosea*	L.	1	5	2	0	18
Rosaceae	*Alchemilla*	*conjuncta* aggr.		0	0	0	0	1
Rosaceae	*Alchemilla*	*mollis*	(Buser) Rothm.	0	1	0	0	1

Rosaceae	*Alchemilla*	*vulgaris aggr.*		1	1	1	6	20
Rosaceae	*Alchemilla*	*speciosa*	Buser	0	0	0	0	1
Droseraceae	*Aldrovanda*	*vesiculosa*	L.	0	0	0	0	1
Alismataceae	*Alisma*	*lanceolatum*	With.	0	1	0	0	1
Alismataceae	*Alisma*	*plantago-aquatica*	L.	0	0	0	0	2
Brassicaceae	*Alliaria*	*petiolata*	(M. Bieb.) Cavara & Grande	0	5	2	1	23
Amaryllidaceae	*Allium*	*angulosum*	L.	0	1	0	0	1
Amaryllidaceae	*Allium*	*carinatum*	L.	0	0	0	0	1
Amaryllidaceae	*Allium*	*cernuum*	Roth	0	1	0	0	0
Amaryllidaceae	*Allium*	*cristophii*	Trautv.	0	1	0	0	1
Amaryllidaceae	*Allium*	*giganteum*	Regel	1	0	1	0	9
Amaryllidaceae	*Allium*	*mairei*	H.Lév.	0	0	0	0	1
Amaryllidaceae	*Allium*	*marschalianum*	Vved.	0	1	0	0	0
Amaryllidaceae	*Allium*	*moly*	L.	0	1	0	0	0
Amaryllidaceae	*Allium*	*nigrum*	L.	0	0	0	0	1
Amaryllidaceae	*Allium*	*oleraceum*	L.	0	0	0	2	0
Amaryllidaceae	*Allium*	*sativum*	L.	0	1	0	0	0
Amaryllidaceae	*Allium*	*schoenoprasum*	L.	0	2	0	0	11
Amaryllidaceae	*Allium*	sp.		0	1	0	0	0
Amaryllidaceae	*Allium*	*sphaerocephalon*	L.	0	0	0	0	8
Amaryllidaceae	*Allium*	*tuberosum*	Rottler ex Spreng.	0	1	0	0	0
Amaryllidaceae	*Allium*	*ursinum*	L.	0	1	0	0	19
Amaryllidaceae	*Allium*	*victorialis*	L.	0	0	0	0	1
Xanthorrhoeaceae	*Aloe*	*aristata*	Haw.	0	1	0	0	0

Xanthorrhoeaceae	*Aloe*	*vera*	(L.) Burm. f.	0	0	0	0	1
Verbenaceae	*Aloysia*	*citriodora*	Palau	0	0	0	0	1
Verbenaceae	*Aloysia*	*polystachya*	(Griseb.) Moldenke	0	0	0	0	1
Alstroemeriaceae	*Alstroemeria*	*aurea*	Graham	0	2	0	0	0
Amaranthaceae	*Alternanthera*	sp.		0	1	0	0	0
Malvaceae	*Althaea*	*cannabina*	L.	0	1	0	0	0
Malvaceae	*Althaea*	*officinalis*	L.	0	0	1	0	3
Brassicaceae	*Alyssum*	*montanum*	L.	0	1	0	0	0
Brassicaceae	*Alyssum*	*murale*	Waldst. & Kit.	1	1	0	0	0
Amaranthaceae	*Amaranthus*	*albus*	L.	0	1	0	0	0
Amaranthaceae	*Amaranthus*	*caudatus*	L.	0	0	0	0	1
Amaranthaceae	*Amaranthus*	*hybridus aggr.*		0	0	0	0	1
Amaryllidaceae	*Amaryllis*	*belladonna*	L.	0	1	0	0	0
Rosaceae	*Amelanchier*	*canadensis*	(L.) Medik.	0	0	0	0	1
Rosaceae	*Amelanchier*	*lamarckii*	F. G. Schroed	0	0	0	0	8
Apiaceae	*Ammi*	*majus*	L.	0	1	0	0	0
Apiaceae	*Ammi*	*visnaga*	(L.) Lam.	0	1	0	0	3
Fabaceae	*Amorpha*	*ouachitensis*	Wilbur	0	1	0	0	0
Apocynaceae	*Amsonia*	*orientalis*	Decne.	1	1	0	0	0
Apocynaceae	*Amsonia*	*tabernaemontana*	Walter	0	1	0	0	0
Orchidaceae	*Anacamptis*	*pyramidalis*	(L.) Rich.	0	1	0	0	0
Asteraceae	*Anacyclus*	*pyrethrum*	(L.) Lag.	0	1	0	0	0
Primulaceae	*Anagallis*	*arvensis*	L.	0	8	0	0	9
Primulaceae	*Anagallis*	*tenella*	(L.) L.	0	0	0	0	1

Asteraceae	*Anaphalis*	*margaritacea*	(L.) Benth. & Hook.f.	0	1	0	0	0
Boraginaceae	*Anchusa*	*arvensis*	(L.) M. Bieb.	0	1	1	2	2
Boraginaceae	*Anchusa*	*italica*	Retz.	0	1	0	0	0
Boraginaceae	*Anchusa*	*officinalis*	L.	0	2	0	0	0
Ericaceae	*Andromeda*	*polifolia*	L.	0	0	0	0	1
Primulaceae	*Androsace*	*villosa*	L.	0	1	0	0	0
Primulaceae	*Androsace*	*vitaliana*	(L.) Lapeyr.	0	0	0	0	1
Ranunculaceae	*Anemone*	*armena*	Boiss.	0	0	0	0	1
Ranunculaceae	*Anemone*	*blanda*	Schott & Kotschy	0	0	0	0	3
Ranunculaceae	*Anemone*	*canadensis*	L.	0	0	0	0	1
Ranunculaceae	*Pulsatilla*	*halleri*	(All.) Willd.	0	0	0	0	1
Ranunculaceae	*Anemone*	*hupehensis*	(É.Lemoine) É. Lemoine	1	3	2	0	5
Ranunculaceae	*Pulsatilla*	*montana*	(Hoppe) Rchb.	0	0	0	0	1
Ranunculaceae	*Anemone*	*multifida*	Poir.	0	1	0	0	0
Ranunculaceae	*Anemone*	*nemorosa*	L.	0	2	0	1	9
Ranunculaceae	*Anemone*	*palmata*	L.	0	1	0	0	0
Ranunculaceae	*Pulsatilla*	*vulgaris*	Mill.	0	0	0	0	1
Ranunculaceae	*Anemone*	*ranunculoides*	L.	0	0	0	4	1
Ranunculaceae	*Anemone*	*rivularis*	Buch.-Ham. ex DC.	0	0	0	0	1
Ranunculaceae	*Anemone*	*rupestris*	Thomson	0	0	0	0	1
Ranunculaceae	*Anemone*	*sylvestris*	L.	0	1	0	0	1
Saururaceae	*Anemopsis*	*californica*	(Nutt.) Hook. & Arn.	0	0	0	0	1

Apiaceae	*Angelica*	*archangelica*	L.	0	0	0	0	1
Apiaceae	*Angelica*	*glauca*	Edgew.	0	1	0	0	0
Malvaceae	*Anoda*	*cristata*	(L.) Schltdl.	0	1	0	0	0
Asteraceae	*Anthemis*	*arvensis*	L.	0	0	0	2	1
Asteraceae	*Anthemis*	*cotula*	L.	0	1	0	0	0
Asteraceae	*Anthemis*	*maritima*	L.	0	1	0	0	0
Asteraceae	*Anthemis*	*marschalliana*	Willd.	0	1	0	0	0
Asteraceae	*Anthemis*	*tinctoria*	L.	0	0	0	0	8
Asparagaceae	*Anthericum*	*liliago*	L.	0	2	0	0	1
Asparagaceae	*Anthericum*	*ramosum*	L.	0	0	0	0	7
Apiaceae	*Anthriscus*	*cerefolium*	(L.) Hoffm.	0	1	0	0	0
Apiaceae	*Anthriscus*	*sylvestris*	(L.) Hoffm.	4	8	2	10	13
Fabaceae	*Anthyllis*	*hermanniae*	L.	0	1	0	0	0
Fabaceae	*Anthyllis*	*vulneraria*	L.	0	1	0	0	14
Plantaginaceae	*Antirrhinum*	*latifolium*	Mill.	0	1	0	0	0
Plantaginaceae	*Antirrhinum*	*majus*	L.	0	2	0	0	16
Asparagaceae	*Aphyllanthes*	*monspeliensis*	L.	0	1	0	0	1
Apiaceae	*Apium*	*graveolens*	L.	0	1	0	0	0
Apiaceae	*Apium*	*nodiflorum*	(L.) Lag.	0	1	0	0	1
Apocynaceae	*Apocynum*	*androsaemifolium*	L.	0	1	0	0	0
Apocynaceae	*Apocynum*	*cannabinum*	L.	0	1	0	0	0
Ranunculaceae	*Aquilegia*	*alpina*	L.	0	1	0	0	0
Ranunculaceae	*Aquilegia*	*ecalcarata*	Maxim.	0	0	0	0	1
Ranunculaceae	*Aquilegia*	*einseleana*	F. W. Schultz	0	1	0	0	0
Ranunculaceae	*Aquilegia*	*glandulosa*	Fisch. ex Link.	0	1	0	0	0
Ranunculaceae	*Aquilegia*	*olympica*	Boiss.	0	1	0	0	0

Ranunculaceae	*Aquilegia*	*vulgaris*	L.	0	4	4	4	33
Brassicaceae	*Arabidopsis*	*thaliana*	(L.) Heynh.	0	2	1	0	4
Brassicaceae	*Arabis*	*caucasica*	Willd.	0	1	0	0	0
Brassicaceae	*Arabis*	*hirsuta*	(L.) Scop.	0	0	0	0	4
Brassicaceae	*Arabis*	*procurrens*	Waldst. & Kit.	0	1	0	0	0
Brassicaceae	*Arabis*	*pumila*	Jacq.	0	1	0	0	0
Brassicaceae	*Arabis*	*soyeri*	Reut. & Huet	0	1	0	0	0
Ericaceae	*Arbutus*	*unedo*	L.	0	1	0	0	0
Asteraceae	*Arctium*	*lappa*	L.	1	1	0	0	4
Asteraceae	*Arctium*	*minus*	Bernh.	0	2	0	0	0
Asteraceae	*Arctium*	*tomentosum*	Mill.	0	0	2	0	0
Caryophyllaceae	*Arenaria*	*purpurascens*	Ramond ex DC.	0	1	0	0	0
Caryophyllaceae	*Arenaria*	*serpyllifolia aggr.*		3	1	0	0	24
Caryophyllaceae	*Arenaria*	*tetraquetra*	L.	0	1	0	0	0
Papaveraceae	*Argemone*	*albiflora*	Hornem.	0	1	0	0	0
Papaveraceae	*Argemone*	*mexicana*	L.	0	1	0	0	1
Asteraceae	*Argyranthemum*	*frutescens*	(L.) Sch. Bip.	3	1	1	0	10
Asteraceae	*Argyranthemum*	sp.		0	1	0	0	0
Fabaceae	*Argyrocytisus*	*battandieri*	(Maire) Raynaud	0	2	0	0	0
Fabaceae	*Argyrolobium*	*zanonii*	(Turra) P. W. Ball	0	0	0	0	1
Araceae	*Arisaema*	*sikokianum*	Franch. & Sav.	0	0	0	0	1
Iridaceae	*Aristea*	*ecklonii*	Baker	0	1	0	0	0
Iridaceae	*Aristea*	*platycaulis*	Baker	0	1	0	0	0
Aristolochiaceae	*Aristolochia*	*clematitis*	L.	0	0	0	0	2
Plumbaginaceae	*Armeria*	*alpina*	Willd.	0	1	0	0	0

Plumbaginaceae	*Armeria*	*canescens*	(Host) Boiss.	0	1	0	0	0
Plumbaginaceae	*Armeria*	*maritima*	(Mill.) Willd.	0	1	0	0	3
Plumbaginaceae	*Armeria*	*splendens*	(Lag. & Rodr.) Webb	0	1	0	0	0
Brassicaceae	*Armoracia*	*rusticana*	P. Gaertn. & al.	0	1	0	0	0
Asteraceae	*Artemisia*	*abrotanum*	L.	0	0	0	0	1
Asteraceae	*Artemisia*	*vulgaris*	L.	0	0	0	0	1
Asparagaceae	*Arthropodium*	*cirrhatum*	(G.Forst.) R.Br.	0	0	0	0	1
Araceae	*Arum*	*italicum*	Mill.	0	0	0	0	1
Rosaceae	*Aruncus*	*dioicus*	(Walter) Fernald	0	0	0	2	2
Apocynaceae	*Asclepias*	*incarnata*	L.	0	0	0	0	1
Apocynaceae	*Asclepias*	*purpurascens*	L.	0	1	0	0	0
Apocynaceae	*Asclepias*	*syriaca*	L.	0	1	0	0	1
Apocynaceae	*Asclepias*	*tuberosa*	L.	0	0	0	0	1
Asparagaceae	*Asparagus*	*maritimus*	(L.) Mill.	0	1	0	0	0
Rubiaceae	*Asperula*	*suberosa*	Sibth. & Sm.	0	1	0	0	0
Rubiaceae	*Asperula*	*taurina*	L.	0	0	0	0	1
Rubiaceae	*Asperula*	*tinctoria*	L.	0	0	0	0	1
Xanthorrhoeaceae	*Asphodeline*	*lutea*	(L.) Rchb.	0	2	0	0	2
Xanthorrhoeaceae	*Asphodeline*	*taurica*	(Pall.) Endl.	0	1	0	0	0
Xanthorrhoeaceae	*Asphodelus*	*acaulis*	Desf.	0	1	0	0	0
Xanthorrhoeaceae	*Asphodelus*	*fistulosus*	L.	0	2	0	0	0
Xanthorrhoeaceae	*Asphodelus*	*macrocarpus*	Parl.	0	1	0	0	0
Asteraceae	*Aster*	*alpinus*	L.	0	1	0	0	0
Asteraceae	*Aster*	*novae-angliae*	L.	1	2	1	0	4
Asteraceae	*Aster*	*pyrenaeus*	Desf. ex DC.	0	1	0	0	0

Asteraceae	Not identified	Not identified		3	1	0	1	0
Saxifragaceae	*Astilbe*	sp.		0	0	0	0	1
Saxifragaceae	*Astilboides*	*tabularis*	(Hemsl.) Engl.	0	1	0	0	0
Fabaceae	*Astragalus*	*cicer*	L.	0	1	0	0	1
Fabaceae	*Astragalus*	*glycyphyllos*	L.	0	0	1	0	1
Fabaceae	*Astragalus*	*onobrychis*	L.	0	1	0	0	0
Fabaceae	*Astragalus*	*sempervirens*	Lam.	0	0	0	0	1
Apiaceae	*Astrantia*	*bavarica*	F. W. Schultz	0	0	0	0	1
Apiaceae	*Astrantia*	*carniolica*	Wulfen	0	0	0	0	1
Apiaceae	*Astrantia*	*major*	L.	1	1	0	0	4
Amaranthaceae	*Atriplex*	*hortensis*	L.	0	0	0	0	3
Solanaceae	*Atropa*	*bella-donna*	L.	0	0	0	0	1
Brassicaceae	*Aubrieta*	*deltoidea*	(L.) DC.	0	2	0	0	8
Brassicaceae	*Aurinia*	*petraea*	(Ard.) Schur	0	1	0	0	0
Brassicaceae	*Aurinia*	*saxatilis*	(L.) Desv.	1	1	0	0	4
Alismataceae	*Baldellia*	*ranunculoides*	(L.) Parl.	0	0	0	0	1
Lamiaceae	*Ballota*	*hirsuta*	Benth.	0	1	0	0	0
Lamiaceae	*Ballota*	*nigra*	L.	0	1	4	0	0
Fabaceae	*Baptisia*	*australis*	(L.) R. Br.	0	1	0	0	0
Brassicaceae	*Barbarea*	*vulgaris*	R. Br.	1	0	7	12	13
Begoniaceae	*Begonia*	× *semperflorens-cultorum*	H. K. Krauss	6	2	1	0	9
Begoniaceae	*Begonia*	sp.		2	1	0	2	10
Asteraceae	*Bellis*	*perennis*	L.	11	12	11	8	32
Berberidaceae	*Berberis*	*julianae*	C.K.Schneid.	0	1	0	0	4
Berberidaceae	*Berberis*	*thunbergii*	DC.	0	0	0	0	8

Berberidaceae	*Berberis*	*vulgaris*	L.	0	0	0	0	3
Saxifragaceae	*Bergenia*	*ciliata*	(Haw.) Strenb.	0	0	0	0	1
Saxifragaceae	*Bergenia*	*crassifolia*	(L.) Fritsch	0	1	0	1	6
Saxifragaceae	*Bergenia*	*stracheyi*	Engl.	0	0	0	0	1
Asteraceae	*Berkheya*	*purpurea*	Mast.	0	1	0	0	0
Brassicaceae	*Berteroa*	*incana*	(L.) DC.	0	1	6	1	0
Amaranthaceae	*Beta*	*vulgaris*	L.	0	0	0	0	1
Betulaceae	*Betula*	*alleghaniensis*	Britton	0	0	0	0	1
Betulaceae	*Betula*	*pendula*	Roth	0	0	0	0	5
Betulaceae	*Betula*	*pubescens aggr.*		0	0	0	0	3
Asteraceae	*Bidens*	*bipinnata*	L.	0	1	0	0	0
Asteraceae	*Bidens*	*cernua*	L.	0	0	0	0	1
Asteraceae	*Bidens*	*ferulifolia*	(Jacq.) Sweet	1	0	0	0	3
Asteraceae	*Bidens*	*frondosa*	L.	1	0	0	0	0
Asteraceae	*Bidens*	*lemmonii*	A.Gray	0	1	0	0	0
Asteraceae	*Bidens*	*pilosa*	L.	1	1	0	0	0
Asteraceae	*Bidens*	sp.		0	1	0	0	0
Bignoniaceae	*Bignonia*	*capreolata*	L.	0	1	0	0	0
Brassicaceae	*Biscutella*	*laevigata*	L.	0	1	0	0	1
Fabaceae	*Bituminaria*	*bituminosa*	(L.) C. H. Stirt.	0	1	0	0	0
Gentianaceae	*Blackstonia*	*perfoliata*	(L.) Huds.	0	1	0	0	1
Orchidaceae	*Bletilla*	*striata*	(Thunb.) Rchb. f.	0	1	0	0	1
Urticaceae	*Boehmeria*	*nivea*	(L.) Gaudich.	0	0	0	0	1
Boraginaceae	*Borago*	*officinalis*	L.	0	3	0	1	13
Asteraceae	*Borrichia*	*frutescens*	(L.) DC.	0	1	0	0	0
Nyctaginaceae	*Bougainvillea*	sp.		0	0	0	0	4

Asteraceae	*Brachyglottis*	*greyi*	(Hook. f.) B. Nord.	0	1	0	0	1
Brassicaceae	*Brassica*	*juncea*	(L.) Czern.	0	0	0	0	1
Brassicaceae	*Brassica*	*napus*	L.	0	0	0	0	2
Brassicaceae	*Brassica*	*nigra*	(L.) W. D. J. Koch	0	0	0	0	1
Brassicaceae	*Brassica*	*oleracea*	L.	0	1	0	0	2
Brassicaceae	*Brassica*	*rapa*	L.	0	1	1	0	6
Brassicaceae	*Brassica*	sp.		0	2	0	0	0
Asparagaceae	*Brimeura*	*amethystina*	(L.) Chouard.	0	1	0	0	0
Solanaceae	*Brugmansia*	*pittieri*	(Saff.) Moldenke	0	0	0	0	1
Solanaceae	*Brugmansia*	sp.		1	0	0	0	0
Boraginaceae	*Brunnera*	*macrophylla*	(Adams) I. M. Johnst.	0	2	0	0	6
Cucurbitaceae	*Bryonia*	*cretica*	L.	0	0	1	0	2
Cucurbitaceae	*Bryonia*	*dioica*	Jacq.	0	0	0	0	2
Scrophulariaceae	*Buddleja*	*davidii*	Franch.	3	7	1	0	13
Scrophulariaceae	*Buddleja*	*glomerata*	H. L. Wendl.	0	1	0	0	0
Boraginaceae	*Buglossoides*	*purpurocaerulea*	(L.) I. M. Johnst.	0	3	0	0	1
Xanthorrhoeaceae	*Bulbine*	*annua*	(L.) Willd.	0	1	0	0	0
Brassicaceae	*Bunias*	*orientalis*	L.	0	1	0	1	0
Apiaceae	*Bunium*	*bulbocastanum*	L.	0	0	0	0	1
Asteraceae	*Buphthalmum*	*salicifolium*	L.	0	2	0	1	4
Apiaceae	*Bupleurum*	*falcatum*	L.	0	1	0	0	0
Apiaceae	*Bupleurum*	*fruticosum*	L.	0	1	0	0	0
Butomaceae	*Butomus*	*umbellatus*	L.	0	0	0	1	1
Buxaceae	*Buxus*	*balearica*	Lam.	0	1	0	0	0

Buxaceae	*Buxus*	*sempervirens*	L.	0	0	0	0	3
Fabaceae	*Caesalpinia*	*gilliesii*	(Hook.) D. Dietr.	0	1	0	0	0
Calceolariaceae	*Calceolaria*	*integrifolia*	L.	0	1	0	0	0
Calceolariaceae	*Calceolaria*	sp.		0	1	0	0	0
Asteraceae	*Calendula*	*arvensis*	L.	0	1	0	0	6
Asteraceae	*Calendula*	*officinalis*	L.	2	2	2	3	14
Solanaceae	*Calibrachoa*	*parviflora*	(Juss.) D'Arcy	0	1	0	0	0
Solanaceae	*Calibrachoa*	sp.		2	0	1	0	0
Araceae	*Calla*	*palustris*	L.	0	1	0	0	0
Myrtaceae	*Callistemon*	*citrinus*	(Curtis) Skeels	0	1	0	0	1
Myrtaceae	*Callistemon*	*salignus*	(Sm.) Colv. ex Sweet	0	1	0	0	0
Myrtaceae	*Callistemon*	sp.		0	0	0	0	1
Asteraceae	*Callistephus*	*chinensis*	(L.) Nees	0	0	0	0	3
Ericaceae	*Calluna*	*vulgaris*	(L.) Hull	1	0	0	1	4
Ranunculaceae	*Caltha*	*leptosepala*	DC.	0	0	0	0	1
Ranunculaceae	*Caltha*	*palustris*	L.	0	1	0	1	6
Calycanthaceae	*Calycanthus*	*chinensis*	Chang Pt. Li	0	0	0	0	1
Calycanthaceae	*Calycanthus*	*floridus*	L.	0	1	0	0	0
Calycanthaceae	*Calycanthus*	*occidentalis*	Hook. & Arn.	0	0	0	0	1
Convolvulaceae	*Calystegia*	*sepium*	(L.) R. Br.	6	7	2	3	30
Asparagaceae	*Camassia*	*leichtlinii*	(Baker) S.Watson	0	0	0	0	1
Asparagaceae	*Camassia*	sp.		0	1	0	0	0
Brassicaceae	*Camelina*	*sativa*	(L.) Crantz	0	0	0	0	1
Theaceae	*Camellia*	*japonica*	L	0	1	0	0	1

Theaceae	*Camellia*	sp.		0	0		0	0	4
Campanulaceae	*Campanula*	*alliariifolia*	Willd.	0	1		0	0	0
Campanulaceae	*Campanula*	*americana*	L.	0	0		0	0	1
Campanulaceae	*Campanula*	*barbata*	L.	0	0		0	0	1
Campanulaceae	*Campanula*	*carpatica*	Jacq.	0	1		0	0	0
Campanulaceae	*Campanula*	*cervicaria*	L.	0	0		0	0	1
Campanulaceae	*Campanula*	*cochleariifolia*	Lam.	0	1		0	0	2
Campanulaceae	*Campanula*	*fenestrellata*	Feer	0	1		0	0	0
Campanulaceae	*Campanula*	*glomerata*	L.	0	1		0	0	6
Campanulaceae	*Campanula*	*grossekii*	Heuff.	0	1		0	0	0
Campanulaceae	*Campanula*	*latifolia*	L.	0	3		0	0	0
Campanulaceae	*Campanula*	*medium*	L.	0	1		0	0	7
Campanulaceae	*Campanula*	*patula*	L.	0	1		0	4	0
Campanulaceae	*Campanula*	*persicifolia*	L.	4	1		1	0	6
Campanulaceae	*Campanula*	*portenschlagiana*	Schult.	2	4		0	0	10
Campanulaceae	*Campanula*	*poscharskyana*	Degen	2	2		1	0	5
Campanulaceae	*Campanula*	*rapunculoides*	L.	0	1		0	0	9
Campanulaceae	*Campanula*	*rapunculus*	L.	0	2		0	0	4
Campanulaceae	*Campanula*	*rhomboidalis*	L.	0	1		0	0	0
Campanulaceae	*Campanula*	*rotundifolia*	L.	2	2		0	1	10
Campanulaceae	*Campanula*	*sarmatica*	Ker Gawl.	0	0		0	0	1
Campanulaceae	*Campanula*	sp.		1	1		0	0	0
Campanulaceae	*Campanula*	*spicata*	L.	0	0		0	0	1
Campanulaceae	*Campanula*	*tommasiniana*	K.Koch	0	1		0	0	0
Campanulaceae	*Campanula*	*trachelium*	L.	1	0		2	0	6

Bignoniaceae	*Campsis*	*grandiflora*	(Thunb.) K. Schum.	0	1	0	0	0
Bigoniaceae	*Campsis*	*radicans*	(L.) Seem.	1	2	0	0	2
Cannaceae	*Canna*	*indica*	L.	0	1	0	0	0
Cannaceae	*Canna*	sp.		0	1	0	0	0
Cannabaceae	*Cannabis*	*sativa*	L.	0	0	0	0	1
Brassicaceae	*Capsella*	*bursa-pastoris*	(L.) Medik.	3	11	9	10	24
Solanaceae	*Capsicum*	*annuum*	L.	1	0	0	0	7
Solanaceae	*Capsicum*	*pubescens*	Ruiz & Pav.	0	0	0	0	1
Fabaceae	*Caragana*	*brevispina*	Benth.	0	1	0	0	0
Brassicaceae	*Cardamine*	*amara*	L.	0	0	0	0	4
Brassicaceae	*Cardamine*	*bulbifera*	(L.) Crantz	0	0	0	0	1
Brassicaceae	*Cardamine*	*flexuosa* aggr.		0	0	0	0	7
Brassicaceae	*Cardamine*	*heptaphylla*	(Vill.) O. E. Schulz	0	0	0	0	6
Brassicaceae	*Cardamine*	*hirsuta*	L.	1	1	0	0	16
Brassicaceae	*Cardamine*	*impatiens*	L.	1	0	1	0	0
Brassicaceae	*Cardamine*	*kitaibelii*	Bech.	0	0	0	0	1
Brassicaceae	*Cardamine*	*pentaphyllos*	(L.) Crantz	0	0	0	0	1
Brassicaceae	*Cardamine*	*pratensis* aggr.		0	0	0	0	23
Brassicaceae	*Cardamine*	*× killiasii*	(Brügger) Brügger	0	0	0	0	1
Sapindaceae	*Cardiospermum*	*grandiflorum*	Sw.	0	0	0	0	1
Sapindaceae	*Cardiospermum*	*halicacabum*	L.	0	0	0	0	1
Asteraceae	*Carduus*	*crispus*	L.	0	3	1	2	0
Asteraceae	*Carduus*	*defloratus*	L.	0	0	0	0	1
Asteraceae	*Carduus*	*nutans*	L.	0	1	0	0	0

Asteraceae	*Carduus*	sp.		0	0	0	0	1
Asteraceae	*Carlina*	*vulgaris* aggr.		0	0	0	0	1
Fabaceae	*Carmichaelia*	*stevensonii*	(Cheesman) Heenan	0	1	0	0	0
Hydrangenaceae	*Carpenteria*	*californica*	Torr.	0	1	0	0	0
Betulaceae	*Carpinus*	*betulus*	L.	0	0	0	0	7
Betulaceae	*Carpinus*	*laxiflora*	(Siebold & Zucc.) Blume	0	0	0	0	1
Asteraceae	*Carthamus*	*caeruleus*	L.	0	1	0	0	0
Asteraceae	*Carthamus*	*tinctorius*	L.	0	0	0	0	1
Apiaceae	*Carum*	*carvi*	L.	0	1	1	7	4
Lamiaceae	*Caryopteris*	*× clandonensis*	A. Simmonds ex Rehd.	0	0	0	0	3
Fagaceae	*Castanea*	*sativa*	Mill.	1	0	0	0	0
Bignoniaceae	*Catalpa*	*bignonioides*	Walter	3	1	0	0	1
Asteraceae	*Catananche*	*caerulea*	L.	0	1	0	0	0
Apocynaceae	*Catharanthus*	*roseus*	(L.) G. Don	2	0	0	0	0
Rhamnaceae	*Ceanothus*	*americanus*	L.	0	1	0	0	0
Rhamnaceae	*Ceanothus*	*thyrsiflorus*	Eschw.	0	1	0	0	0
Rhamnaceae	*Ceanothus*	*× delilianus*	Spach	0	0	0	0	1
Celastraceae	*Celastrus*	*orbiculatus*	Thunb.	0	0	0	0	1
Amaranthaceae	*Celosia*	*argentea*	L.	1	4	0	0	2
Asteraceae	*Centaurea*	*aspera*	L.	0	2	0	0	0
Asteraceae	*Centaurea*	*benedicta*	(L.) L.	0	0	0	0	1
Asteraceae	*Centaurea*	*calcitrapa*	L.	0	1	0	0	0
Asteraceae	*Centaurea*	*collina*	L.	0	1	0	0	0

Asteraceae	*Centaurea*	*cyanus*	L.	0	5	1	0	13
Asteraceae	*Centaurea*	*jacea*	L.	3	2	1	9	26
Asteraceae	*Centaurea*	*macrocephala*	Muss. Puschk. ex Willd.	0	1	0	0	0
Asteraceae	*Centaurea*	*montana*	L.	0	1	2	2	7
Asteraceae	*Centaurea*	*nigra*	L.	0	1	0	0	0
Asteraceae	*Centaurea*	*nigrescens*	Willd.	0	1	0	0	0
Asteraceae	*Centaurea*	*scabiosa*	L.	0	2	0	0	11
Asteraceae	*Centaurea*	*solstitialis*	L.	0	1	0	0	0
Gentianaceae	*Centaurium*	*erythraea*	Rafn	0	1	0	0	2
Caprifoliaceae	*Centranthus*	*angustifolius*	(Mill.) DC.	0	0	0	0	1
Caprifoliaceae	*Centranthus*	*calcitrapa*	(L.) Dufr.	0	1	0	0	0
Caprifoliaceae	*Centranthus*	*ruber*	(L.) DC.	0	7	0	0	7
Orchidaceae	*Cephalanthera*	*damasonium*	(Mill.) Druce	0	0	0	0	2
Caprifoliaceae	*Cephalaria*	*alpina*	(L.) Roem. & Schult.	0	1	0	0	1
Caprifoliaceae	*Cephalaria*	*leucantha*	(L.) Schrad. ex Roem. & Schult.	0	1	0	0	0
Caryophyllaceae	*Cerastium*	*arvense*	L.	0	2	0	12	1
Caryophyllaceae	*Cerastium*	*brachypetalum*	Pers.	3	0	0	0	3
Caryophyllaceae	*Cerastium*	*candidissimum*	Correns	0	1	0	0	0
Caryophyllaceae	*Cerastium*	*fontanum*	Baumg.	0	3	6	7	27
Caryophyllaceae	*Cerastium*	*glomeratum*	Thuill.	0	1	0	0	18
Caryophyllaceae	*Cerastium*	*pumilum* aggr.		0	1	0	0	2
Caryophyllaceae	*Cerastium*	*semidecandrum*	L.	0	1	0	0	0
Caryophyllaceae	*Cerastium*	*tomentosum*	L.	3	1	1	3	3

Plumbaginaceae	*Ceratostigma*	*plumbaginoides*	Bunge	0	3	0	0	3
Plumbaginaceae	*Ceratostigma*	*willmottianum*	Stapf	0	3	0	0	0
Fabaceae	*Cercis*	*canadensis*	L.	0	1	0	0	0
Fabaceae	*Cercis*	*siliquastrum*	L.	0	3	0	0	4
Boraginaceae	*Cerinthe*	*minor*	L.	0	1	0	0	1
Solanaceae	*Cestrum*	*parqui*	(Lam.) L'Hér.	0	1	0	0	0
Rosaceae	*Chaenomeles*	*× superba*	(Frahm) Rehder	0	1	0	0	12
Plantaginaceae	*Chaenorhinum*	*origanifolium*	(L.) Kostel.	0	1	0	0	0
Plantaginaceae	*Chaenorrhinum*	*minus*	(L.) Lange	0	1	0	0	13
Scrophulariaceae	*Chaenostoma*	*cordatum*	(Thunb.) Benth.	2	0	0	0	2
Apiaceae	*Chaerophyllum*	*aureum*	L.	0	1	0	0	0
Apiaceae	*Chaerophyllum*	*bulbosum*	L.	0	1	0	0	0
Apiaceae	*Chaerophyllum*	*temulum*	L.	0	4	0	0	0
Fabaceae	*Chamaecytisus*	*hirsutus*	(L.) Link	0	1	0	0	0
Asteraceae	*Chamaemelum*	*nobile*	(L.) All.	0	0	0	0	1
Asteraceae	*Cheirolophus*	*benoistii*	(Humbert) Holub	0	1	0	0	0
Papaveraceae	*Chelidonium*	*majus*	L.	3	8	11	7	21
Amaranthaceae	*Chenopodium*	*album* aggr.		0	2	0	0	5
Amaranthaceae	*Chenopodium*	*ambrosioides*	L.	0	0	0	0	1
Amaranthaceae	*Chenopodium*	*polyspermum*	L.	0	0	0	0	1
Amaranthaceae	*Chenopodium*	*quinoa*	Willd.	0	0	0	0	1
Bignoniaceae	*Chilopsis*	*linearis*	(Cav.) Sweet	0	1	0	0	0
Oleaceae	*Chionanthus*	*virginicus*	L.	0	0	0	0	1
Bignoniaceae	*Chitalpa*	*tashkentensis*	T.S.Elias & Wisura	0	0	0	0	1
Rutaceae	*Choisya*	*ternata*	Kunth	1	8	0	0	0

Asteraceae	*Chondrilla*	*juncea*	L.	0	1	0	0	0
Asteraceae	*Chrysanthemum*	× hortorum	hort.	0	0	1	1	4
Asteraceae	*Chrysogonum*	*virginianum*	L.	0	1	0	0	1
Saxifragaceae	*Chrysosplenium*	*alternifolium*	L.	0	0	0	1	0
Fabaceae	*Cicer*	*arietinum*	L.	0	0	0	0	1
Asteraceae	*Cichorium*	*endivia*	L.	0	0	1	0	0
Asteraceae	*Cichorium*	*intybus*	L.	0	3	7	0	15
Onagraceae	*Circaea*	*lutetiana*	L.	0	0	0	0	24
Asteraceae	*Cirsium*	*arvense*	(L.) Scop.	5	8	2	1	17
Asteraceae	*Cirsium*	*eriophorum*	(L.) Scop.	0	1	0	0	0
Asteraceae	*Cirsium*	*erisithales*	(Jacq.) Scop.	0	0	0	0	1
Asteraceae	*Cirsium*	*helenioides*	(L.) Hill	0	0	0	0	1
Asteraceae	*Cirsium*	*monspessulanum*	(L.) Hill	0	1	0	0	0
Asteraceae	*Cirsium*	*palustre*	(L.) Scop.	0	1	0	0	3
Asteraceae	*Cirsium*	sp.		0	1	0	0	0
Asteraceae	*Cirsium*	*tuberosum*	(L.) All.	0	1	0	0	0
Asteraceae	*Cirsium*	*vulgare*	(Savi) Ten.	2	5	0	3	13
Cistaceae	*Cistus*	*albidus*	L.	0	1	0	0	1
Cistaceae	*Cistus*	*ladanifer*	L.	0	5	0	0	0
Cistaceae	*Cistus*	*monspeliensis*	L.	0	2	0	0	0
Cistaceae	*Cistus*	*salviifolius*	L.	0	1	0	0	2
Cistaceae	*Cistus*	sp.		0	1	0	0	0
Cistaceae	*Cistus*	× *nigricans*	Pourr.	0	1	0	0	0
Cistaceae	*Cistus*	× *purpureus*	Lam.	0	1	0	0	0
Cucurbitaceae	*Citrullus*	*colocynthis*	(L.) Schrad.	0	0	0	0	1

Cucurbitaceae	*Citrullus*	*lanatus*	(Thunb.) Matsum. & Nakai	1	0		0	0	0
Rutaceae	*Citrus*	*limon*	(L.) Osbeck	0	1		0	0	1
Rutaceae	*Citrus*	*medica*	L.	0	0		0	0	1
Asteraceae	*Cladanthus*	*arabicus*	(L.) Cass.	0	1		0	0	0
Onargaceae	*Clarkia*	*amoena*	Macbr.	0	1		0	0	0
Montiaceae	*Claytonia*	*perfoliata*	Willd.	1	1		0	0	0
Ranunculaceae	*Clematis*	*integrifolia*	L.	0	1		0	0	1
Ranunculaceae	*Clematis*	*montana*	Buch.-Ham. ex DC.	0	0		0	0	2
Ranunculaceae	*Clematis*	*orientalis*	L.	0	1		0	0	0
Ranunculaceae	*Clematis*	*recta*	L.	0	1		0	0	0
Ranunculaceae	*Clematis*	sp.		0	0		0	0	8
Ranunculaceae	*Clematis*	*stans*	Siebold & Zucc.	0	0		0	0	1
Ranunculaceae	*Clematis*	*tangutica*	(Maxim.) Korsh.	0	1		0	0	0
Ranunculaceae	*Clematis*	*vitalba*	L.	0	3		0	0	4
Ranunculaceae	*Clematis*	*viticella*	L.	0	0		0	0	1
Cleomaceae	*Cleome*	*houtteana*	Schltdl.	0	2		0	0	3
Lamiaceae	*Acinos*	*alpinum*	(L.) Moench	0	1		0	0	0
Lamiaceae	*Calamintha*	*grandiflora*	(L.) Moench	0	1		0	0	0
Lamiaceae	*Clinopodium*	*nepeta*	(L.) Kuntze	0	2		0	0	5
Lamiaceae	*Calamintha*	*thymifolium*	(Scop.) Rchb.	0	1		0	0	0
Lamiaceae	*Clinopodium*	*vulgare*	L.	0	1		5	0	7
Amaryllidaceae	*Clivia*	*miniata*	(Lindl.) Bosse	0	0		0	0	1
Apiaceae	*Cnidium*	*dubium*	Thell.	0	0		0	1	0
Asteraceae	*Coleostephus*	*myconis*	(L.) Rchb. f.	0	1		0	0	0

Lamiaceae	*Coleus*	sp.		0	1	0	0	0
Fabaceae	*Colutea*	*arborescens*	L.	0	3	1	0	0
Fabaceae	*Colutea*	*persica*	Boiss.	0	1	0	0	0
Apiaceae	*Conium*	*maculatum*	L.	0	1	0	0	4
Ranunculaceae	*Consolida*	*ajacis*	(L.) Schur	0	1	0	0	0
Ranunculaceae	*Consolida*	*regalis*	Gray	0	1	0	0	0
Asparagaceae	*Convallaria*	*majalis*	L.	0	2	2	0	11
Convolvulaceae	*Convolvulus*	*arvensis*	L.	0	9	6	0	14
Convolvulaceae	*Convolvulus*	*cantabrica*	L.	0	0	0	0	1
Convolvulaceae	*Convolvulus*	*sabatius*	Viv.	0	1	0	0	0
Convolvulaceae	*Calystegia*	*sylvatica*	(Kit.) Griseb.	0	1	0	0	0
Convolvulaceae	*Convolvulus*	*tricolor*	L.	0	1	0	0	5
Malvaceae	*Corchorus*	*olitorius*	L.	0	0	0	0	1
Asteraceae	*Coreopsis*	*lanceolata*	L.	0	1	0	0	2
Asteraceae	*Coreopsis*	sp.		0	0	0	0	1
Asteraceae	*Coreopsis*	*tinctoria*	Nutt.	0	1	0	0	3
Apiaceae	*Coriandrum*	*sativum*	L.	0	2	0	0	3
Cornaceae	*Cornus*	*alba*	L.	0	1	0	0	2
Cornaceae	*Cornus*	*controversa*	Hemsl.	0	0	0	0	9
Cornaceae	*Cornus*	*florida*	L.	0	1	0	0	0
Cornaceae	*Cornus*	*kousa*	F. Buerger ex Hance	0	0	0	0	7
Cornaceae	*Cornus*	*mas*	L.	0	0	0	0	5
Cornaceae	*Cornus*	*sanguinea*	L.	0	6	4	2	10
Cornaceae	*Cornus*	*sericea*	L.	4	2	2	1	19
Cornaceae	*Cornus*	sp.		0	0	0	1	0

Argophyllaceae	*Corokia*	*cotoneaster*	Raoul	0	0	0	0	1
Fabaceae	*Coronilla*	*scorpioides*	(L.) W. D. J. Koch	0	1	0	0	0
Fabaceae	*Coronilla*	*vaginalis*	Lam.	0	0	0	0	1
Fabaceae	*Coronilla*	*valentina*	L.	0	2	0	0	0
Papaveraceae	*Corydalis*	*flexuosa*	Franch.	0	1	0	0	0
Papaveraceae	*Corydalis*	*lutea*	(L.) DC.	0	1	1	0	1
Papaveraceae	*Corydalis*	*saxicola*	Bunting	0	0	0	0	1
Papaveraceae	*Corydalis*	*solida*	(L.) Clairv.	0	1	0	4	0
Hamamelidaceae	*Corylopsis*	*himalayana*	Griff.	0	0	0	0	1
Hamamelidaceae	*Corylopsis*	*sinensis*	Hemsl.	0	0	0	0	1
Hamamelidaceae	*Corylopsis*	*spicata*	Siebold & Zucc.	0	0	0	0	1
Asteraceae	*Cosmos*	*bipinnatus*	Cav.	0	4	0	2	15
Asteraceae	*Cosmos*	*parviflorus*	(Jacq.) Pers.	0	1	0	0	0
Asteraceae	*Cosmos*	*sulphureus*	Cav.	1	3	0	0	13
Asteraceae	*Cota*	*altissima*	(L.) J.Gay	0	1	0	0	0
Asteraceae	*Cota*	*tinctoria*	(L.) J.Gay	0	1	0	0	0
Anacardiaceae	*Cotinus*	*coggygria*	Scop.	0	6	0	0	2
Rosaceae	*Cotoneaster*	*coriaceus*	Franch.	0	1	0	0	0
Rosaceae	*Cotoneaster*	*dammeri*	C. K. Schneid.	0	0	0	0	1
Rosaceae	*Cotoneaster*	*divaricatus*	Rehder & E. H. Wilson	0	0	0	0	7
Rosaceae	*Cotoneaster*	*horizontalis*	Decne.	3	1	2	0	9
Rosaceae	*Cotoneaster*	*integrifolius*	(Roxb.) G. Klotz	0	2	0	0	0
Rosaceae	*Cotoneaster*	*microphyllus*	Wall. ex Lindl.	0	1	0	0	0
Rosaceae	*Cotoneaster*	*salicifolius*	Franch.	0	0	0	0	2
Rosaceae	*Cotoneaster*	*zabelii*	C. K. Schneid.	0	1	0	0	0

Asteraceae	*Cotula*	*coronopifolia*	L.	1	0		0	0	1
Brassicaceae	*Crambe*	*maritima*	L.	0	1		0	0	0
Crassulaceae	*Crassula*	*muricata*	Thunb.	0	1		0	0	0
Rosaceae	*Crataegus*	*crus-galli*	L.	0	1		0	0	0
Rosaceae	*Crataegus*	*laevigata*	(Poir.) DC.	0	0		0	0	9
Rosaceae	*Crataegus*	*monogyna*	Jacq.	1	1		1	0	9
Rosaceae	*Crataegus*	sp.		0	1		0	0	0
Asteraceae	*Crepis*	*biennis*	L.	0	0		0	0	23
Asteraceae	*Crepis*	*capillaris*	Wallr.	4	5		4	0	31
Asteraceae	*Crepis*	*foetida*	L.	0	1		0	0	0
Asteraceae	*Crepis*	sp.		0	0		0	0	5
Asteraceae	*Crepis*	*pyrenaica*	(L.) Greuter	0	1		0	0	0
Asteraceae	*Crepis*	*tectorum*	L.	0	1		0	0	0
Asteraceae	*Crepis*	*vesicaria*	L.	0	0		0	0	21
Iridaceae	*Crocosmia*	*× crocosmiiflora*	(Lemoine) N. E. Br.	0	0		0	0	1
Rubiaceae	*Cruciata*	*glabra*	(L.) Ehrend.	0	0		0	0	3
Rubiaceae	*Cruciata*	*laevipes*	Opiz	0	1		0	0	0
Apiaceae	*Cryptotaenia*	*japonica*	Hassk.	0	1		0	0	0
Cucurbitaceae	*Cucumis*	*melo*	L.	0	0		0	0	6
Cucurbitaceae	*Cucumis*	*sativus*	L.	0	0		0	0	11
Cucurbitaceae	*Cucurbita*	*maxima*	Duchesne	0	0		0	0	11
Cucurbitaceae	*Cucurbita*	*moschata*	Duchesne	0	0		0	0	1
Cucurbitaceae	*Cucurbita*	*pepo*	L.	0	0		0	2	11
Lythraceae	*Cuphea*	*hyssopifolia*	Kunth	0	0		0	0	1
Lythraceae	*Cuphea*	*ignea*	A. DC.	0	0		0	0	5

Primulaceae	*Cyclamen*	*cilicium*	Boiss. & Heldr.	0	1	0	0	0
Primulaceae	*Cyclamen*	*purpurascens*	Mill.	0	0	0	0	1
Primulaceae	*Cyclamen*	*repandum*	Sm.	0	1	0	0	0
Plantaginaceae	*Cymbalaria*	*muralis*	P. Gaertn. & al.	1	5	0	0	6
Asteraceae	*Cynara*	*cardunculus subsp. cardunculus*	L.	0	1	0	0	0
Asteraceae	*Cynara*	*cardunculus subsp. scolymus*	(L.) Berger	0	1	0	0	2
Boraginaceae	*Cynoglossum*	*amabile*	Stapf & J. R. Drumm.	0	0	0	0	3
Orchidaceae	*Cypripedium*	*calceolus*	L.	0	0	0	0	1
Cyrillaceae	*Cyrilla*	*racemiflora*	L.	0	0	0	0	1
Fabaceae	*Cytisophyllum*	*sessilifolium*	(L.) O.Lang	0	1	0	0	0
Fabaceae	*Cytisus*	*decumbens*	(Durande) Spach	0	0	0	0	1
Fabaceae	*Cytisus*	*emeriflorus*	Rchb.	0	0	0	0	1
Fabaceae	*Cytisus*	*hirsutus*	L.	0	0	0	0	1
Fabaceae	*Cytisus*	*nigricans*	L.	0	1	0	0	0
Fabaceae	*Cytisus*	*scoparius*	(L.) Link	0	1	0	0	2
Fabaceae	*Cytisus*	*spinescens*	C. Presl	0	0	0	0	1
Papaveraceae	*Dactylicapnos*	*torulosa*	Hutch.	0	0	0	0	1
Orchidaceae	*Dactylorhiza*	*fuchsii*	(Druce) Soó	0	1	0	1	1
Orchidaceae	*Dactylorhiza*	*maculata*	(L.) Soó	0	0	0	1	1
Orchidaceae	*Dactylorhiza*	*praetermissa*	(Druce) Soó	0	1	0	0	0
Asteraceae	*Dahlia*	*merckii*	Lehm.	0	1	0	0	0
Asteraceae	*Dahlia*	*× hortensis*	Cav.	1	4	1	1	3
Thymelaeaceae	*Daphne*	*alpina*	L.	0	1	0	0	1

Thymelaeaceae	*Daphne*	*tangutica*	Maxim.	0	1	0	0	0
Solanaceae	*Datura*	sp.		1	0	0	0	0
Solanaceae	*Datura*	*stramonium*	L.	0	0	0	0	2
Apiaceae	*Daucus*	*carota*	L.	0	3	2	0	29
Hydrangenaceae	*Decumaria*	*barbara*	L.	0	1	0	0	0
Hydrangenaceae	*Deinanthe*	*caerulea*	Stapf	0	1	0	0	1
Aizoaceae	*Delosperma*	*cooperi*	(Hook.) L. Bolus	0	2	0	0	2
Aizoaceae	*Delosperma*	*nubigenum*	(Schltr.) L. Bolus	0	1	0	0	0
Aizoaceae	*Delosperma*	*sutherlandii*	(Hook.) N. E. Br.	0	1	0	0	0
Ranunculaceae	*Delphinium*	*delavayi*	Franch.	0	1	0	0	0
Ranunculaceae	*Delphinium*	*dubium*	(Rouy & Foucaud) Pawl.	0	0	0	0	3
Ranunculaceae	*Delphinium*	*× cultorum*	L.	1	3	0	1	6
Ranunculaceae	*Delphinium*	*formosum*	Boiss. & A.Huet	0	1	0	0	0
Ranunculaceae	*Delphinium*	*montanum*	DC.	0	1	0	0	0
Ranunculaceae	*Delphinium*	*pictum*	Willd.	0	1	0	0	0
Ranunculaceae	*Delphinium*	*speciosum*	M.Bieb.	0	1	0	0	0
Ranunculaceae	*Delphinium*	*staphisagria*	L.	0	1	0	0	0
Fabaceae	*Desmodium*	*canadense*	(L.) DC.	0	1	0	0	0
Hydrangenaceae	*Deutzia*	*discolor*	Hemsl.	0	1	0	0	0
Hydrangenaceae	*Deutzia*	*gracilis*	Siebold & Zucc.	0	1	0	0	0
Hydrangenaceae	*Deutzia*	*scabra*	Thunb.	4	6	1	0	12
Caryophyllaceae	*Dianthus*	*armeria*	L.	0	2	1	0	15
Caryophyllaceae	*Dianthus*	*arpadianus*	Ade & Bornm.	0	1	0	0	0
Caryophyllaceae	*Dianthus*	*barbatus*	L.	1	1	0	0	11
Caryophyllaceae	*Dianthus*	*carthusianorum*	L.	0	1	0	0	12

Caryophyllaceae	*Dianthus*	*caryophyllus*	L.	0	0	0	0	1
Caryophyllaceae	*Dianthus*	*chinensis*	L.	3	0	1	0	4
Caryophyllaceae	*Dianthus*	*deltoides*	L.	0	2	0	0	2
Caryophyllaceae	*Dianthus*	*furcatus*	Balb.	0	1	0	0	0
Caryophyllaceae	*Dianthus*	*giganteus*	d'Urv.	0	1	0	0	1
Caryophyllaceae	*Dianthus*	*gratianopolitanus*	Vill.	0	1	0	0	1
Caryophyllaceae	*Dianthus*	*hoeltzeri*	C.Winkl.	0	0	0	0	1
Caryophyllaceae	*Dianthus*	*petraeus*	Waldst. & Kit.	0	1	0	0	0
Caryophyllaceae	*Dianthus*	*pinifolius*	Sm.	0	1	0	0	0
Caryophyllaceae	*Dianthus*	*plumarius*	L.	0	7	0	0	0
Caryophyllaceae	*Dianthus*	*pyrenaicus*	Pourr.	0	1	0	0	0
Caryophyllaceae	*Dianthus*	*seguieri*	Vill.	0	0	0	0	1
Caryophyllaceae	*Dianthus*	*serotinus*	Waldst. & Kit.	0	1	0	0	0
Caryophyllaceae	*Dianthus*	sp.		0	1	0	0	11
Caryophyllaceae	*Dianthus*	*sternbergii*	Sieber ex Capelli	0	1	0	0	0
Caryophyllaceae	*Dianthus*	*superbus*	L.	0	0	0	0	2
Scrophulariaceae	*Diascia*	*barberae*	Hook. f.	0	0	0	0	2
Scrophulariaceae	*Diascia*	*rigescens*	E. Mey. ex Benth.	0	1	0	0	0
Papaveraceae	*Dicentra*	*eximia*	(Ker Gawl.) Torr.	0	0	0	0	1
Papaveraceae	*Dicentra*	*formosa*	(Haw.) Walp.	0	1	0	0	0
Asparagaceae	*Dichelostemma*	*congestum*	(Sm.) Kunth	0	1	0	0	0
Asparagaceae	*Dichelostemma*	*ida-maia*	(Alph.Wood) Greene	0	1	0	0	0
Acanthaceae	*Dicliptera*	*squarrosa*	Nees	0	0	0	0	1
Rutaceae	*Dictamnus*	*albus*	L.	0	2	0	0	0

Iridaceae	*Dierama*	*medium*	N. E. Br.	0	1		0	0	0
Iridaceae	*Dierama*	*pulcherrimum*	(Hook.f.) Baker	0	1		0	0	0
Iridaceae	*Dietes*	*bicolor*	(Steud.) Sweet ex Klatt	0	1		0	0	0
Plantaginaceae	*Digitalis*	*ferruginea*	L.	0	1		0	0	0
Plantaginaceae	*Digitalis*	*grandiflora*	Mill.	0	0		0	0	2
Plantaginaceae	*Digitalis*	*lutea*	L.	0	3		0	0	1
Plantaginaceae	*Digitalis*	*parviflora*	Jacq.	0	1		0	0	0
Plantaginaceae	*Digitalis*	*purpurea*	L.	2	2		0	0	3
Droseraceae	*Dionaea*	*muscipula*	J. Ellis	0	0		0	0	1
Caprifoliaceae	*Dipelta*	*floribunda*	Maxim.	0	1		0	0	0
Brassicaceae	*Diplotaxis*	*erucoides*	(L.) DC.	0	2		0	0	0
Brassicaceae	*Diplotaxis*	*muralis*	(L.) DC.	0	0		0	0	1
Brassicaceae	*Diplotaxis*	*tenuifolia*	(L.) DC.	0	1		1	0	8
Caprifoliaceae	*Dipsacus*	*fullonum*	L.	0	0		0	0	7
Caprifoliaceae	*Dipsacus*	*sativus*	(L.) Honck.	0	1		0	0	0
Rhamnaceae	*Discaria*	*chacaye*	(G. Don) Tortosa	0	1		0	0	0
Asteraceae	*Doellingeria*	*umbellata*	(Mill.) Nees	0	1		0	0	0
Asteraceae	*Doronicum*	*columnae*	Ten.	0	0		0	3	0
Asteraceae	*Doronicum*	*grandiflorum*	Lam.	0	1		0	0	0
Asteraceae	*Doronicum*	*pardalianches*	L.	0	1		0	0	1
Fabaceae	*Dorycnium*	*germanicum*	(Gremli) Rikli	0	0		0	0	1
Fabaceae	*Dorycnium*	*herbaceum*	Vill.	0	0		0	0	1
Fabaceae	*Dorycnium*	*hirsutum*	(L.) Ser.	0	1		0	0	0
Fabaceae	*Dorycnium*	*pentaphyllum*	Scop.	0	1		0	0	0
Fabaceae	*Dorycnium*	*rectum*	(L.) Ser.	0	1		0	0	0

Brassicaceae	*Draba*	*aizoides*	L.	0	0	0	0	1
Brassicaceae	*Draba*	*muralis*	L.	0	1	0	0	1
Brassicaceae	*Draba*	sp.		0	0	0	0	2
Lamiaceae	*Dracocephalum*	*foetidum*	Bunge	0	1	0	0	0
Apocynaceae	*Dregea*	*sinensis*	Hemsl.	0	1	0	0	0
Aizoaceae	*Drosanthemum*	*floribundum*	(Haw.) Schwantes	1	0	0	0	0
Droseraceae	*Drosera*	*anglica*	Huds.	0	0	0	0	1
Droseraceae	*Drosera*	*capensis*	L	0	0	0	0	1
Droseraceae	*Drosera*	*hamiltonii*	C. R. P. Andrews	0	0	0	0	1
Rosaceae	*Dryas*	*octopetala*	L.	0	0	0	0	1
Rosaceae	*Potentilla*	*rupestris*	L.	0	1	0	0	0
Rosaceae	*Duchesnea*	*indica*	(Andrews) Focke	2	9	1	0	14
Fabaceae	*Ebenus*	*cretica*	L.	0	1	0	0	0
Cucurbitaceae	*Ecballium*	*elaterium*	(L.) A. Rich.	0	1	0	0	2
Crassulaceae	*Echeveria*	sp.		0	0	0	0	1
Asteraceae	*Echinacea*	*pallida*	(Nutt.) Nutt.	0	1	0	0	0
Asteraceae	*Echinacea*	*purpurea*	(L.) Moench	3	1	0	1	7
Alismataceae	*Echinodorus*	*cordifolius*	(L.) Griseb.	0	1	0	0	0
Asteraceae	*Echinops*	*ritro*	L.	0	2	0	0	0
Asteraceae	*Echinops*	*sphaerocephalus*	L.	1	1	0	0	7
Boraginaceae	*Echium*	*plantagineum*	L.	0	2	1	0	1
Boraginaceae	*Echium*	*vulgare*	L.	0	3	6	0	12
Campanulaceae	*Edraianthus*	*tenuifolius*	(A. DC.) A. DC.	0	1	0	0	0
Elaeagnaceae	*Elaeagnus*	*angustifolia*	L.	0	0	0	0	1
Lamiaceae	*Elsholtzia*	*ciliata*	(Thunb.) Hyl.	0	1	0	0	0

Lamiaceae	*Elsholtzia*	*stauntonii*	Benth.	0	1	0	0	0
Proteaceae	*Embothrium*	*coccineum*	J. R. Forst. & G. Forst.	0	1	0	0	0
Papaveraceae	*Eomecon*	*chionantha*	Hance	0	0	0	0	1
Ephedraceae	*Ephedra*	*altissima*	Desf.	0	1	0	0	0
Ephedraceae	*Ephedra*	*equisetina*	Bunge	0	1	0	0	0
Onagraceae	*Epilobium*	*angustifolium*	L.	0	1	0	2	4
Onagraceae	*Epilobium*	*canum*	(Greene) P. H. Raven	0	1	0	0	0
Onagraceae	*Epilobium*	*roseum*	DC.	2	2	0	0	14
Onagraceae	*Epilobium*	*dodonaei*	Vill.	0	1	0	0	1
Onagraceae	*Epilobium*	*hirsutum*	L.	3	4	1	0	20
Onagraceae	*Epilobium*	*montanum*	L.	1	1	0	0	11
Onagraceae	*Epilobium*	*parviflorum*	Schreb.	2	0	0	0	12
Onagraceae	*Epilobium*	*tetragonum*	L.	0	0	0	1	0
Berberidaceae	*Epimedium*	*alpinum*	L.	0	1	0	0	1
Berberidaceae	*Epimedium*	*perralderianum*	Coss.	0	0	0	0	1
Berberidaceae	*Epimedium*	*pinnatum*	Fisch. ex DC	0	0	0	0	2
Berberidaceae	*Epimedium*	*× versicolor*	E. Morren	0	0	0	0	1
Berberidaceae	*Epimedium*	sp.		0	1	0	0	6
Orchidaceae	*Epipactis*	*palustris*	(L.) Crantz	0	0	0	0	1
Caryophyllaceae	*Eremogone*	*gypsophiloides*	(L.) Fenzl	0	1	0	0	0
Caryophyllaceae	*Eremogone*	*longifolia*	(M. Bieb.) Fenzl	0	1	0	0	0
Xanthorrhoeaceae	*Eremurus*	sp.		0	0	0	0	1
Ericaceae	*Erica*	*carnea*	L.	0	0	0	0	1
Ericaceae	*Erica*	*cinerea*	L.	0	1	0	0	0

Ericaceae	*Erica*	*erigena*	R.Ross	0	1	0	0	0
Ericaceae	*Erica*	*tetralix*	L.	0	0	0	0	2
Asteraceae	*Erigeron*	*acris*	L.	0	1	0	0	0
Asteraceae	*Erigeron*	*annuus*	(L.) Desf.	3	3	6	1	27
Asteraceae	*Erigeron*	*caespitosus*	Nutt.	0	1	0	0	0
Asteraceae	*Erigeron*	*canadensis*	L.	7	1	0	6	19
Asteraceae	*Erigeron*	*glabellus*	Nutt.	0	1	0	0	0
Asteraceae	*Erigeron*	*glabratus*	Bluff & Fingerh.	0	1	0	0	0
Asteraceae	*Erigeron*	*karvinskianus*	DC.	1	2	0	0	0
Plantaginaceae	*Erinus*	*alpinus*	L.	0	2	0	0	0
Polygonaceae	*Eriogonum*	*grande*	Greene	0	1	0	0	0
Polygonaceae	*Eriogonum*	*umbellatum*	Torr.	0	1	0	0	0
Geraniaceae	*Erodium*	*absinthoides*	Willd.	0	1	0	0	1
Geraniaceae	*Erodium*	*botrys*	(Cav.) Bertol.	0	1	0	0	0
Geraniaceae	*Erodium*	*chrysanthum*	L'Hér.	0	1	0	0	0
Geraniaceae	*Erodium*	*ciconium*	(L.) L'Hér.	1	0	2	0	0
Geraniaceae	*Erodium*	*cicutarium*	(L.) L'Hér.	2	1	3	1	2
Geraniaceae	*Erodium*	*malacoides*	(L.) L'Hér.	0	1	0	0	0
Geraniaceae	*Erodium*	*manescavi*	Coss.	0	2	0	0	0
Geraniaceae	*Erodium*	*trifolium*	(Cav.) Guitt.	0	1	0	0	0
Geraniaceae	*Erodium*	*× hybridum*	Sünderm.	0	0	0	0	1
Brassicaceae	*Erophila*	*verna* aggr.		0	0	0	0	2
Brassicaceae	*Eruca*	*vesicaria*	(L.) Cav.	0	1	0	0	0
Brassicaceae	*Erucastrum*	*gallicum*	(Willd.) O. E. Schulz	0	1	0	4	1
Brassicaceae	*Erucastrum*	*nasturtiifolium*	(Poir.) O. E. Schulz	0	0	0	0	5

Apiaceae	*Eryngium*	*alpinum*	L.	0	0		0	0	2
Apiaceae	*Eryngium*	*amethystinum*	L.	0	1		0	0	1
Apiaceae	*Eryngium*	*bourgatii*	Gouan	0	1		0	0	0
Apiaceae	*Eryngium*	*campestre*	L.	0	1		0	0	0
Apiaceae	*Eryngium*	*giganteum*	M. Bieb.	0	1		0	0	1
Apiaceae	*Eryngium*	*integrifolium*	Walter	0	0		0	0	1
Apiaceae	*Eryngium*	*maritimum*	L.	0	0		0	0	1
Apiaceae	*Eryngium*	*planum*	L.	0	1		0	0	0
Brassicaceae	*Erysimum*	*asperum*	(Nutt.) DC.	0	1		0	0	0
Brassicaceae	*Erysimum*	*capitatum*	(Douglas) Greene	0	1		0	0	1
Brassicaceae	*Erysimum*	*cheiranthoides*	L.	0	1		2	0	4
Brassicaceae	*Erysimum*	*cheiri*	(L.) Crantz	0	5		0	0	1
Brassicaceae	*Erysimum*	*perofskianum*	Fisch. & C.A.Mey.	0	1		0	0	0
Brassicaceae	*Erysimum*	*rhaeticum*	(Hornem.) DC.	0	0		0	0	1
Brassicaceae	*Erysimum*	*sylvestre*	(Crantz) Scop.	0	0		0	0	1
Fabaceae	*Erythrina*	*crista-galli*	L.	0	0		0	0	1
Liliaceae	*Erythronium*	*dens-canis*	L.	0	0		0	1	0
Escalloniaceae	*Escallonia*	*rubra*	(Ruiz & Pav.) Pers.	0	1		0	0	0
Escalloniaceae	*Escallonia*	*× demissa*	Eastw.	0	1		0	0	0
Papaveraceae	*Eschscholzia*	*californica*	Cham.	0	2		1	0	10
Celastraceae	*Euonymus*	*europaeus*	L.	1	1		0	0	9
Celastraceae	*Euonymus*	*fortunei*	(Turcz.) Hand.-Mazz.	1	0		0	0	0
Celastraceae	*Euonymus*	*grandiflorus*	Wall.	0	1		0	0	0

Celastraceae	*Euonymus*	*japonicus*	Thunb.	0	0	0	0	1
Celastraceae	*Euonymus*	*sachalinensis*	(F. Schmidt) Maxim.	0	0	0	0	1
Asteraceae	*Eupatorium*	*cannabinum*	L.	3	3	0	0	7
Asteraceae	*Eupatorium*	*perfoliatum*	L.	0	1	0	0	0
Asteraceae	*Eupatorium*	*purpureum*	L.	0	1	0	0	0
Euphorbiaceae	*Euphorbia*	*amygdaloides*	L.	0	4	0	0	2
Euphorbiaceae	*Euphorbia*	*characias*	L.	1	2	0	0	3
Euphorbiaceae	*Euphorbia*	*cyparissias*	L.	0	1	1	2	1
Euphorbiaceae	*Euphorbia*	*dulcis*	L.	0	1	0	0	0
Euphorbiaceae	*Euphorbia*	*epithymoides*	L.	0	0	0	0	1
Euphorbiaceae	*Euphorbia*	*esula*	L.	0	2	0	0	0
Euphorbiaceae	*Euphorbia*	*exigua*	L.	0	1	0	0	1
Euphorbiaceae	*Euphorbia*	*griffithii*	Hook. f.	0	0	0	0	1
Euphorbiaceae	*Euphorbia*	*helioscopia*	L.	0	0	0	0	19
Euphorbiaceae	*Euphorbia*	*hypericifolia*	L.	1	0	0	0	3
Euphorbiaceae	*Euphorbia*	*illirica*	Lam.	0	1	0	0	0
Euphorbiaceae	*Euphorbia*	*lathyris*	L.	0	5	0	0	0
Euphorbiaceae	*Euphorbia*	*myrsinites*	L.	0	1	0	0	0
Euphorbiaceae	*Euphorbia*	*palustris*	L.	0	1	0	0	1
Euphorbiaceae	*Euphorbia*	*peplus*	L.	1	2	0	0	13
Euphorbiaceae	*Euphorbia*	*platyphyllos*	L.	0	1	0	0	2
Euphorbiaceae	*Euphorbia*	*serrata*	L.	0	1	0	0	0
Euphorbiaceae	*Euphorbia*	sp.		0	1	0	0	22
Euphorbiaceae	*Euphorbia*	*spinosa*	L.	0	1	0	0	0
Euphorbiaceae	*Euphorbia*	*stricta*	L.	0	1	0	0	0

Euphorbiaceae	*Euphorbia*	*umbellata*	(Pax) Bruyns	0	1	0	0	0
Euphorbiaceae	*Euphorbia*	*verrucosa*	L.	0	0	0	0	1
Orobanchaceae	*Euphrasia*	*stricta*	J. F. Lehm.	1	0	0	0	0
Asteraceae	*Euryops*	*chrysanthemoides*	(DC.) B. Nord.	0	1	0	0	0
Rosaceae	*Exochorda*	*racemosa*	(Lindl.) Rehder	0	1	0	0	1
Solanaceae	*Fabiana*	*imbricata*	Ruiz & Pav.	0	1	0	0	0
Polygonaceae	*Fagopyrum*	*acutatum*	Hammer	0	1	0	0	0
Polygonaceae	*Fagopyrum*	*esculentum*	Moench	0	1	0	0	4
Polygonaceae	*Fagopyrum*	*tataricum*	(L.) Gaertn.	0	1	0	0	0
Fagaceae	*Fagus*	*sylvatica*	L.	1	0	0	0	1
Polygonaceae	*Fallopia*	*aubertii*	(L. Henry) Holub	0	1	0	0	0
Polygonaceae	*Fallopia*	*baldschuanica*	(Regel) Holub	0	1	0	0	0
Apiaceae	*Ferula*	*assa-foetida*	L.	0	0	0	0	1
Moraceae	*Ficus*	*carica*	L.	0	0	0	0	1
Rosaceae	*Filipendula*	*camschatica*	(Pall.) Maxim.	0	0	0	0	1
Rosaceae	*Filipendula*	*ulmaria*	(L.) Maxim.	1	1	0	1	16
Rosaceae	*Filipendula*	*vulgaris*	Moench	0	1	0	0	2
Malvaceae	*Firmiana*	*simplex*	(L.) W. Wight	0	1	0	0	0
Phyllanthaceae	*Flueggea*	*virosa*	(Roxb. ex Willd.) Royle	0	1	0	0	0
Apiaceae	*Foeniculum*	*vulgare*	Mill.	0	2	0	0	13
Oleaceae	*Forsythia*	*suspensa*	(Thunb.) Vahl	0	1	0	0	0
Oleaceae	*Forsythia*	*viridissima*	Lindl.	0	1	0	0	0
Oleaceae	*Forsythia*	*× intermedia*	Zabel	0	1	0	0	18
Rosaceae	*Fragaria*	*× ananassa*	(Weston) Raizer	0	0	1	0	15
Rosaceae	*Fragaria*	*vesca*	L.	1	1	0	4	21

Rosaceae	*Fragaria*	*viridis*	Duchesne	0	1	0	0	0
Oleaceae	*Fraxinus*	*excelsior*	L.	0	0	0	0	2
Oleaceae	*Fraxinus*	*ornus*	L.	0	1	0	0	3
Liliaceae	*Fritillaria*	*imperialis*	L.	0	0	0	1	0
Liliaceae	*Fritillaria*	*meleagris*	L.	0	0	0	0	1
Amaranthaceae	*Froelichia*	*floridana*	(Nutt.) Moq.	0	1	0	0	0
Onagraceae	*Fuchsia*	*boliviana*	Carrière	0	0	0	0	1
Onagraceae	*Fuchsia*	*corymbiflora*	Ruiz & Pav.	0	0	0	0	1
Onagraceae	*Fuchsia*	*glazioviana*	Taub.	0	1	0	0	0
Onagraceae	*Fuchsia*	*magellanica*	Lam.	0	4	0	0	1
Onagraceae	*Fuchsia*	*paniculata*	Lindl.	0	0	0	0	1
Onagraceae	*Fuchsia*	*regia*	(Vand. ex Vell.) Munz	0	1	0	0	1
Onagraceae	*Fuchsia*	sp.		1	0	0	0	13
Onagraceae	*Fuchsia*	*× bacillaris*	Lindl.	0	0	0	0	1
Cistaceae	*Fumana*	*ericoides*	(Cav.) Gand.	0	0	0	0	1
Papaveraceae	*Fumaria*	*bastardii*	Boreau	0	1	0	0	0
Papaveraceae	*Fumaria*	*capreolata*	L.	0	1	0	0	0
Papaveraceae	*Fumaria*	*muralis*	Sond. ex W. D. J. Koch	0	1	0	0	0
Papaveraceae	*Fumaria*	*officinalis*	L.	0	2	0	1	6
Papaveraceae	*Fumaria*	*parviflora*	Lam.	0	1	0	0	0
Liliaceae	*Gagea*	*lutea*	(L.) Ker Gawl.	0	0	0	7	0
Liliaceae	*Gagea*	*minima*	(L.) Ker Gawl.	0	0	0	2	0
Asteraceae	*Gaillardia*	*grandiflora*	hort.	1	0	0	0	0
Asteraceae	*Gaillardia*	*grandiflora*	hort.	0	1	0	0	0

Asteraceae	*Gaillardia*	*grandiflora*	hort.	0	1	0	0	0
Asteraceae	*Galactites*	*tomentosa*	Moench	0	1	0	0	0
Amaryllidaceae	*Galanthus*	*nivalis*	L.	0	1	0	0	0
Fabaceae	*Galega*	*officinalis*	L.	0	3	0	0	0
Lamiaceae	*Galeopsis*	*angustifolia*	Hoffm.	0	0	0	0	1
Lamiaceae	*Galeopsis*	*ladanum*	L.	0	0	0	0	1
Lamiaceae	*Galeopsis*	*tetrahit*	L.	0	0	0	0	4
Asteraceae	*Galinsoga*	*parviflora*	Cav.	0	3	0	0	4
Asteraceae	*Galinsoga*	*quadriradiata*	Ruiz & Pav.	6	1	0	0	15
Rubiaceae	*Galium*	*mollugo* aggr.		1	2	0	6	32
Rubiaceae	*Galium*	*aparine*	L.	2	4	0	1	10
Rubiaceae	*Galium*	*arenarium*	Loisel.	0	1	0	0	0
Rubiaceae	*Galium*	*boreale*	L.	0	1	0	0	0
Rubiaceae	*Galium*	*glaucum*	L.	0	1	0	0	0
Rubiaceae	*Galium*	*lucidum*	All.	0	1	0	0	0
Rubiaceae	*Galium*	*odoratum*	(L.) Scop.	0	1	0	0	8
Rubiaceae	*Galium*	*palustre*	L.	0	2	0	0	0
Rubiaceae	*Galium*	*parisiense*	L.	0	4	0	0	0
Rubiaceae	*Galium*	*rubioides*	L.	0	1	0	0	0
Rubiaceae	*Galium*	*saxatile*	L.	0	2	0	0	0
Rubiaceae	*Galium*	*sylvaticum*	L.	0	1	0	0	0
Rubiaceae	*Galium*	*uliginosum*	L.	0	0	0	0	1
Rubiaceae	*Galium*	*verrucosum*	Huds.	0	0	0	0	13
Rubiaceae	*Galium*	*verum*	L.	0	1	0	1	12
Rubiaceae	*Gardenia*	*jasminoides*	J. Ellis	0	1	0	0	0
Garryaceae	*Garrya*	*fadyenii*	Hook.	0	0	0	0	1

Onagraceae	*Gaura*	*biennis*	L.	0	1		0	0	0
Onagraceae	*Gaura*	*lindheimeri*	Engelm. & A. Gray	3	6		0	0	8
Asteraceae	*Gazania*	sp.		2	1		1	0	4
Fabaceae	*Genista*	*anglica*	L.	0	0		0	0	1
Fabaceae	*Genista*	*cinerea*	(Vill.) DC.	0	1		0	0	0
Fabaceae	*Genista*	*hispanica*	L.	0	1		0	0	0
Fabaceae	*Genista*	*pilosa*	L.	0	0		0	0	1
Fabaceae	*Genista*	*sylvestris*	Scop.	0	1		0	0	0
Fabaceae	*Genista*	*tinctoria*	L.	0	5		0	0	1
Gentianaceae	*Gentiana*	*acaulis*	L.	0	0		0	0	2
Gentianaceae	*Gentiana*	*asclepiadea*	L.	0	1		0	0	0
Gentianaceae	*Gentiana*	*campestris*	L.	0	0		0	0	1
Gentianaceae	*Gentiana*	*dinarica*	Beck	0	0		0	0	1
Gentianaceae	*Gentiana*	*lutea*	L.	0	0		0	0	1
Gentianaceae	*Gentiana*	*macrophylla*	Pall.	0	0		0	0	1
Gentianaceae	*Gentiana*	sp.		0	1		0	0	0
Geraniaceae	*Pelargonium*	*elegans*	Willd.	0	0		0	0	1
Geraniaceae	*Geranium*	*albanum*	M. Bieb.	0	1		0	0	0
Geraniaceae	*Geranium*	*argenteum*	L.	0	1		0	0	0
Geraniaceae	*Geranium*	*cinereum*	Cav.	0	0		0	0	2
Geraniaceae	*Geranium*	*columbinum*	L.	0	0		0	0	3
Geraniaceae	*Geranium*	*dalmaticum*	(Beck) Rech. f.	0	1		0	0	0
Geraniaceae	*Geranium*	*dissectum*	L.	0	6		1	0	12
Geraniaceae	*Geranium*	*endressii*	J. Gray	0	7		0	0	2
Geraniaceae	*Geranium*	*himalayense*	Klotzsch	0	1		0	0	2

Geraniaceae	*Geranium*	*ibericum*	Cav.	0	1		0	0	0
Geraniaceae	*Geranium*	*macrorrhizum*	L.	4	5		2	0	16
Geraniaceae	*Geranium*	*maculatum*	L.	0	0		0	0	6
Geraniaceae	*Geranium*	*molle*	L.	3	9		6	0	17
Geraniaceae	*Geranium*	*nodosum*	L.	3	3		0	0	8
Geraniaceae	*Geranium*	*oxonianum*	P. F. Yeo	0	1		0	0	0
Geraniaceae	*Geranium*	*palustre*	L.	0	0		0	1	0
Geraniaceae	*Geranium*	*phaeum*	L.	1	3		1	0	2
Geraniaceae	*Geranium*	*pratense*	L.	4	7		1	3	31
Geraniaceae	*Geranium*	*psilostemon*	Ledeb.	0	1		0	0	0
Geraniaceae	*Geranium*	*purpureum*	Vill.	0	1		0	0	0
Geraniaceae	*Geranium*	*pusillum*	L.	8	6		3	1	1
Geraniaceae	*Geranium*	*pyrenaicum*	Burm. f.	1	4		0	0	3
Geraniaceae	*Geranium*	*reflexum*	L.	0	1		0	0	0
Geraniaceae	*Geranium*	*renardii*	Trautv.	0	1		0	0	4
Geraniaceae	*Geranium*	*richardsonii*	Fisch. & Trautv.	0	1		0	0	5
Geraniaceae	*Geranium*	*robertianum*	L.	6	10		3	0	29
Geraniaceae	*Geranium*	*rotundifolium*	L.	0	2		1	0	1
Geraniaceae	*Geranium*	*sanguineum*	L.	2	3		0	0	17
Geraniaceae	*Geranium*	sp.		9	1		2	0	15
Geraniaceae	*Geranium*	*versicolor*	L.	0	4		0	0	0
Geraniaceae	*Geranium*	*wallichianum*	D.Don ex Sweet	0	0		0	0	1
Geraniaceae	*Geranium*	*yesoense*	Franch. & Sav.	0	1		0	0	0
Rosaceae	*Geum*	*aleppicum*	Jacq.	0	1		0	0	0
Rosaceae	*Geum*	*coccineum*	Sibth. & Sm.	0	1		0	0	1
Rosaceae	*Geum*	*macrophyllum*	Willd.	0	1		0	0	0

Rosaceae	*Geum*	*pyrenaicum*	Mill.	0	1	0	0	0
Rosaceae	*Geum*	*rivale*	L.	0	0	0	3	1
Rosaceae	*Geum*	*urbanum*	L.	3	7	3	7	30
Rosaceae	*Geum*	*× sudeticum*	Tausch	0	0	0	0	1
Polemoniaceae	*Gilia*	*laciniata*	Ruiz & Pav.	0	1	0	0	0
Polemoniaceae	*Gilia*	*tricolor*	Benth.	0	0	0	0	3
Iridaceae	*Gladiolus*	*communis*	L.	0	1	0	0	0
Iridaceae	*Gladiolus*	*× hortulanus*	L.H. Bailey	0	1	0	0	4
Iridaceae	*Gladiolus*	*palustris*	Gaudin	0	0	0	0	1
Verbenaceae	*Glandularia*	*bipinnatifida*	(Schauer) Nutt.	1	0	0	0	0
Verbenaceae	*Glandularia*	sp.		1	0	0	0	3
Papaveraceae	*Glaucium*	*flavum*	Crantz	0	1	0	0	0
Asteraceae	*Glebionis*	*coronaria*	(L.) Cass. Ex Spach	0	1	0	0	1
Asteraceae	*Glebionis*	*segetum*	(L.) Fourr.	0	1	0	0	0
Lamiaceae	*Glechoma*	*hederacea*	L.	2	7	6	11	30
Plantaginaceae	*Globularia*	*bisnagarica*	L.	0	0	0	0	2
Plantaginaceae	*Globularia*	*cordifolia*	L.	0	1	0	0	0
Plantaginaceae	*Globularia*	*trichosantha*	Fisch. & C. A. Mey.	0	1	0	0	0
Plantaginaceae	*Globularia*	*vulgaris*	L.	0	1	0	0	0
Colchicaceae	*Gloriosa*	*superba*	L.	0	0	0	0	1
Fabaceae	*Glycyrrhiza*	*echinata*	L.	0	1	0	0	1
Fabaceae	*Glycyrrhiza*	*glabra*	L.	0	1	0	0	0
Scrophulariaceae	*Gomphostigma*	*virgatum*	(L. f.) Baill.	0	1	0	0	0
Amaranthaceae	*Gomphrena*	*globosa*	L.	0	1	0	0	0
Plumbaginaceae	*Goniolimon*	*tataricum*	(L.) Boiss.	0	1	0	0	0

Plantaginaceae	*Gratiola*	*officinalis*	L.	0	1	0	0	0
Asteraceae	*Grindelia*	*integrifolia*	DC.	0	1	0	0	0
Orchidaceae	*Gymnadenia*	*conopsea*	(L.) R. Br.	1	1	0	0	0
Caryophyllaceae	*Gypsophila*	*elegans*	M. Bieb.	0	0	0	0	2
Caryophyllaceae	*Gypsophila*	*muralis*	L.	0	0	0	0	1
Caryophyllaceae	*Gypsophila*	*paniculata*	L.	2	0	0	0	5
Caryophyllaceae	*Gypsophila*	*repens*	L.	0	1	0	0	3
Caryophyllaceae	*Gypsophila*	*scorzonerifolia*	Ser.	0	1	0	0	0
Caryophyllaceae	*Gypsophila*	*tenuifolia*	M.Bieb.	0	1	0	0	0
Asteraceae	*Haplopappus*	*glutinosus*	Cass.	0	1	0	0	0
Plantaginaceae	*Hebe*	*traversii*	Allan	0	0	0	0	1
Fabaceae	*Hedysarum*	*coronarium*	L.	0	1	0	0	0
Asteraceae	*Helenium*	*aromaticum*	(Hook.) L.H.Bailey	0	0	0	0	1
Asteraceae	*Helenium*	CV		3	2	2	0	4
Cistaceae	*Helianthemum*	*apenninum*	(L.) Mill.	0	1	0	0	0
Cistaceae	*Helianthemum*	*nummularium*	(L.) Mill.	0	1	0	0	1
Asteraceae	*Helianthus*	*annuus*	L.	0	4	1	2	19
Asteraceae	*Helianthus*	*giganteus*	L.	0	1	0	0	0
Asteraceae	*Helianthus*	*hirsutus*	Raf.	0	0	0	0	1
Asteraceae	*Helianthus*	*mollis*	Lam.	0	1	0	0	0
Asteraceae	*Helianthus*	*tuberosus* aggr.		0	1	0	0	3
Asteraceae	*Helichrysum*	*italicum*	(Roth) G. Don	0	1	0	0	2
Asteraceae	*Helichrysum*	*litoreum*	Guss.	0	1	0	0	0
Asteraceae	*Helichrysum*	*sibthorpii*	Rouy	0	1	0	0	0
Asteraceae	*Helichrysum*	*stoechas*	(L.) Moench	0	1	0	0	0

Asteraceae	*Heliopsis*	*helianthoides*	(L.) Sweet	0	1		0	0	7
Boraginaceae	*Heliotropium*	*arborescens*	L.	0	1		0	0	0
Boraginaceae	*Heliotropium*	*europaeum*	L.	0	1		0	0	0
Ranunculaceae	*Helleborus*	*foetidus*	L.	0	0		0	0	2
Ranunculaceae	*Helleborus*	*lividus*	Aiton ex Curtis	0	1		0	0	0
Ranunculaceae	*Helleborus*	*niger*	L.	0	0		0	1	1
Ranunculaceae	*Helleborus*	*viridis*	L.	0	0		0	0	2
Asteraceae	*Picris*	*echioides*	(L.) Holub	0	4		0	0	0
Xanthorrhoeaceae	*Hemerocallis*	*citrina*	Baroni	0	1		0	0	0
Xanthorrhoeaceae	*Hemerocallis*	*fulva*	(L.) L.	1	5		3	1	18
Xanthorrhoeaceae	*Hemerocallis*	*lilio-asphodelus*	L.	0	3		1	0	4
Xanthorrhoeaceae	*Hemerocallis*	*middendorffii*	Trautv. & C. A. Mey.	0	0		0	0	1
Ranunculaceae	*Hepatica*	*nobilis*	Schreb.	0	0		0	1	0
Apiaceae	*Heracleum*	*sphondylium*	L.	2	1		0	1	16
Iridaceae	*Hesperantha*	*coccinea*	Goldblatt & J. C. Manning	0	1		0	0	0
Brassicaceae	*Hesperis*	*matronalis*	L.	0	1		0	3	5
Rosaceae	*Heteromeles*	*arbutifolia*	Greene	0	0		0	0	3
Asteraceae	*Heterotheca*	*villosa*	(Pursh) Shinners	0	1		0	0	0
Saxifragaceae	*Heuchera*	*americana*	L.	1	0		0	0	5
Saxifragaceae	*Heuchera*	*hallii*	A. Gray	0	0		0	0	1
Saxifragaceae	*Heuchera*	*micrantha*	Douglas ex Lindl.	0	1		0	0	0
Saxifragaceae	*Heuchera*	*pubescens*	Pursh	0	1		0	0	0
Saxifragaceae	*Heuchera*	*rubescens*	Torr.	0	2		0	0	0
Saxifragaceae	*Heuchera*	*sanguinea*	Engelm.	0	5		0	0	8

Saxifragaceae	*Heuchera*	*villosa*	Michx.	0	1	0	0	0
Saxifragaceae	*Heuchera*	*× brizoides*	hort. ex Lemoine	0	1	0	0	10
Malvaceae	*Hibiscus*	*cannabinus*	L.	0	1	0	0	0
Malvaceae	*Hibiscus*	*rosa-sinensis*	L.	0	1	0	0	0
Malvaceae	*Hibiscus*	sp.		0	0	0	0	4
Malvaceae	*Hibiscus*	*syriacus*	L.	0	4	0	0	17
Malvaceae	*Hibiscus*	*trionum*	L.	0	0	0	0	4
Asteraceae	*Hieracium*	*aurantiacum*	L.	0	1	0	0	2
Asteraceae	*Hieracium*	*bernardii subsp. Templare*	(Arrigoni) Greuter	0	1	0	0	0
Asteraceae	*Hieracium*	*bombycinum*	Boiss. & Reut. ex Rchb.f.	0	0	0	0	1
Asteraceae	*Hieracium*	*caesium* aggr.		0	1	0	0	0
Asteraceae	*Hieracium*	*cymosum*	L.	0	0	0	0	2
Asteraceae	*Hieracium*	*piloselloides*	L.	3	1	1	0	25
Asteraceae	*Hieracium*	*peletierianum*	Mérat	0	1	0	0	0
Asteraceae	*Hieracium*	*piliferum* aggr.		0	0	0	0	1
Asteraceae	*Hieracium*	*pilosella*	L.	2	1	4	1	20
Asteraceae	*Hieracium*	*sabaudum* aggr.		0	1	0	0	0
Asteraceae	*Hieracium*	sp.		0	1	1	0	10
Asteraceae	*Hieracium*	*tomentosum*	L.	0	1	0	0	1
Asteraceae	*Hieracium*	*sabaudum aggr.*	L.	0	2	0	2	11
Asteraceae	*Hieracium*	*villosum*	Jacq.	0	1	0	0	0
Fabaceae	*Hippocrepis*	*comosa*	L.	0	0	0	0	2
Fabaceae	*Hippocrepis*	*emerus*	(L.) Lassen	0	2	0	0	0

Lamiaceae	*Horminum*	*pyrenaicum*	L.	0	1	0	0	0
Asparagaceae	*Hosta*	*capitata*	(Koidz.) Nakai	0	1	0	0	0
Asparagaceae	*Hosta*	*plantaginea*	(Lam.) Asch.	0	1	0	0	0
Asparagaceae	*Hosta*	*sieboldiana*	(Hook.) Engl.	0	1	0	0	1
Asparagaceae	*Hosta*	CV		0	0	2	0	16
Asparagaceae	*Hosta*	*ventricosa*	Stearn	0	0	0	1	0
Saururaceae	*Houttuynia*	*cordata*	Thunb.	0	2	0	0	1
Cannabaceae	*Humulus*	*lupulus*	L.	0	0	0	1	0
Asparagaceae	*Hyacinthoides*	*non-scripta*	(L.) Rothm.	0	2	0	0	12
Asparagaceae	*Hyacinthus*	*orientalis*	L.	0	0	0	1	1
Hydrangenaceae	*Hydrangea*	*arborescens*	L.	3	2	2	0	0
Hydrangenaceae	*Hydrangea*	*aspera*	D. Don	0	0	0	0	11
Hydrangenaceae	*Hydrangea*	*macrophylla*	(Thunb.) Ser.	5	7	2	1	26
Hydrangenaceae	*Hydrangea*	*paniculata*	Siebold	0	1	0	0	0
Hydrangenaceae	*Hydrangea*	*petiolaris*	Siebold & Zucc.	2	0	1	0	7
Hydrangenaceae	*Hydrangea*	*quercifolia*	W. Bartram	5	1	0	0	7
Hydrangenaceae	*Hydrangea*	*seemannii*	L. Riley	0	1	0	0	0
Papaveraceae	*Hylomecon*	*japonica*	(Thunb.) Prantl & Kündig	0	0	0	0	1
Fabaceae	*Hymenocarpos*	*circinnatus*	(L.) Savi	0	1	0	0	0
Asteraceae	*Hymenonema*	*laconicum*	Boiss. & Heldr. ex Boiss. & Heldr.	0	1	0	0	0
Solanaceae	*Hyoscyamus*	*niger*	L.	0	1	0	0	1
Hypericaceae	*Hypericum*	*androsaemum*	L.	1	2	0	0	9
Hypericaceae	*Hypericum*	*calycinum*	L.	4	0	0	0	9

Hypericaceae	*Hypericum*	*hircinum*	L.	0	1		0	0	0
Hypericaceae	*Hypericum*	*hookerianum*	Wight & Arn.	0	1		0	0	0
Hypericaceae	*Hypericum*	*humifusum*	L.	0	0		1	0	3
Hypericaceae	*Hypericum*	*linariifolium*	Vahl	0	1		0	0	0
Hypericaceae	*Hypericum*	*olympicum*	L.	0	1		0	0	0
Hypericaceae	*Hypericum*	*orientale*	L.	0	1		0	0	0
Hypericaceae	*Hypericum*	*patulum*	Thunb.	0	1		0	0	0
Hypericaceae	*Hypericum*	*perforatum*	L.	3	5		3	0	20
Hypericaceae	*Hypericum*	*prolificum*	L.	1	0		0	0	0
Hypericaceae	*Hypericum*	*radicans*	N. Robson	0	0		0	0	1
Hypericaceae	*Hypericum*	*repens*	L.	0	1		0	0	0
Hypericaceae	*Hypericum*	*sampsonii*	Hance	0	1		0	0	0
Hypericaceae	*Hypericum*	*scabrum*	L.	0	0		0	0	1
Hypericaceae	*Hypericum*	*tetrapterum*	Fr.	0	2		0	0	0
Hypericaceae	*Hypericum*	*umbellatum*	A. Kern.	0	1		0	0	0
Hypericaceae	*Hypericum*	*× inodorum*	Mill.	0	1		0	0	0
Asteraceae	*Hypochaeris*	*maculata*	L.	0	1		0	0	0
Asteraceae	*Hypochaeris*	*radicata*	L.	1	1		7	0	30
Asteraceae	*Hypochaeris*	*uniflora*	Vill.	0	1		0	0	0
Hypoxidaceae	*Hypoxis*	*hemerocallidea*	Lall.	0	1		0	0	0
Lamiaceae	*Hyssopus*	*officinalis*	L.	0	1		0	0	3
Brassicaceae	*Iberis*	*amara*	L.	0	1		0	0	1
Brassicaceae	*Iberis*	*pinnata*	L.	0	1		0	0	0
Brassicaceae	*Iberis*	*saxatilis*	L.	0	0		0	0	6
Brassicaceae	*Iberis*	*sempervirens*	L.	0	4		0	0	10
Brassicaceae	*Iberis*	*umbellata*	L.	1	1		0	0	9

Martyniaceae	*Ibicella*	*lutea*	(Lindl.) Van Eselt.	0	0		0	0	1
Aquifoliaceae	*Ilex*	*aquifolium*	L.	1	2		0	0	9
Balsaminaceae	*Impatiens*	*balfourii*	Hook. f.	0	1		0	0	5
Balsaminaceae	*Impatiens*	*balsamina*	L.	0	1		0	0	0
Balsaminaceae	*Impatiens*	*glandulifera*	Royle	0	1		0	0	5
Balsaminaceae	*Impatiens*	*hawkeri*	W. Bull	0	0		0	0	1
Balsaminaceae	*Impatiens*	*parviflora*	DC.	1	1		4	10	2
Balsaminaceae	*Impatiens*	sp.		1	0		0	0	0
Balsaminaceae	*Impatiens*	*walleriana*	Hook. f.	2	2		1	0	15
Fabaceae	*Indigofera*	*tinctoria*	L.	0	1		0	0	0
Asteraceae	*Inula*	*conyzae*	(Griess.) Meikle	0	0		0	0	2
Asteraceae	*Inula*	*ensifolia*	L.	0	0		0	0	2
Asteraceae	*Inula*	*helenium*	L.	0	0		1	0	3
Asteraceae	*Inula*	*hirta*	L.	0	1		0	0	1
Asteraceae	*Inula*	*salicina*	L.	0	0		0	0	3
Asteraceae	*Inula*	*spiraeifolia*	L.	0	1		0	0	0
Solanaceae	*Iochroma*	*australe*	Griseb.	0	1		0	0	0
Convolvulaceae	*Ipomoea*	*alba*	L.	1	0		0	0	0
Convolvulaceae	*Ipomoea*	*indica*	(Burm.) Merr.	1	1		0	0	0
Convolvulaceae	*Ipomoea*	*purpurea*	(L.) Roth	0	0		0	0	6
Convolvulaceae	*Ipomoea*	*nil*	(L.) Roth	0	1		0	0	0
Convolvulaceae	*Ipomoea*	*quamoclit*	L.	0	1		0	0	0
Convolvulaceae	*Ipomoea*	*repens*	(L.) Lam.	0	1		0	0	0
Convolvulaceae	*Ipomoea*	sp.		3	0		0	0	0
Iridaceae	*Iris*	*barbatula*	Noltie & K. Y. Guan	0	0		0	0	1

Iridaceae	*Iris*	*brevicaulis*	Raf.	0	1	0	0	0
Iridaceae	*Iris*	*domestica*	(L.) Goldblatt & Mabb.	0	1	0	0	0
Iridaceae	*Iris*	*ensata*	Thunb.	0	1	0	0	1
Iridaceae	*Iris*	*graminea*	L.	0	1	0	0	0
Iridaceae	*Iris*	*laevigata*	Fisch.	0	1	0	0	0
Iridaceae	*Iris*	*orientalis*	Mill.	0	1	0	0	0
Iridaceae	*Iris*	*pallida*	Lam.	0	1	0	0	0
Iridaceae	*Iris*	*pseudacorus*	L.	3	3	2	1	8
Iridaceae	*Iris*	*setosa*	Pall. ex Link	0	1	0	0	0
Iridaceae	*Iris*	*sibirica*	L.	0	1	0	2	0
Iridaceae	*Iris*	*sintenisii*	Janka	0	1	0	0	0
Iridaceae	*Iris*	sp.		0	3	4	3	5
Iridaceae	*Iris*	*spuria*	L.	0	1	0	0	0
Iridaceae	*Iris*	*unguicularis*	Poir.	0	1	0	0	0
Iridaceae	*Iris*	*virginica*	L.	0	1	0	0	0
Brassicaceae	*Isatis*	*tinctoria*	L.	0	1	0	0	0
Asteraceae	*Ismelia*	*carinata*	(Schousb.) Sch.Bip.	0	1	0	0	1
Solanaceae	*Jaborosa*	*integrifolia*	Lam.	0	1	0	0	0
Asteraceae	*Senecio*	*cineraria*	DC.	0	2	0	0	4
Hydrangenaceae	*Jamesia*	*americana*	Torr. & A. Gray	0	1	0	0	0
Campanulaceae	*Jasione*	*laevis*	Lam.	0	0	0	0	1
Campanulaceae	*Jasione*	*montana*	L.	2	1	0	0	0
Oleaceae	*Jasminum*	*beesianum*	Forrest & Diels	0	1	0	0	1
Oleaceae	*Jasminum*	*fruticans*	L.	0	4	0	0	0

Oleaceae	*Jasminum*	*humile*	L.	0	1	0	0	0
Oleaceae	*Jasminum*	*parkeri*	Dunn	0	1	0	0	0
Juglandaceae	*Juglans*	*regia*	L.	0	0	0	0	1
Crassulaceae	*Kalanchoe*	*blossfeldiana*	Poelln.	3	0	0	0	0
Asteraceae	*Aster*	*lautureanus*	(Debeaux) Franch.	0	1	0	0	0
Asteraceae	*Aster*	*mongolicus*	Franch.	0	1	0	0	1
Ericaceae	*Kalmia*	*latifolia*	L.	1	0	0	0	0
Brassicaceae	*Kernera*	*saxatilis*	(L.) Sweet	0	1	0	0	0
Rosaceae	*Kerria*	*japonica*	(L.) DC	0	9	0	0	5
Malvaceae	*Kitaibelia*	*vitifolia*	Willd.	0	1	0	0	0
Caprifoliaceae	*Knautia*	*arvensis*	(L.) Coult.	0	2	0	2	18
Caprifoliaceae	*Knautia*	*dipsacifolia*	Kreutzer	0	1	0	0	1
Caprifoliaceae	*Knautia*	*macedonica*	Griseb.	1	0	0	0	3
Xanthorrhoeaceae	*Kniphofia*	*rufa*	sensu Codd, non Bak.	0	1	0	0	0
Xanthorrhoeaceae	*Kniphofia*	CV		0	0	0	0	3
Sapindaceae	*Koelreuteria*	*paniculata*	Laxm.	0	3	0	0	0
Caprifoliaceae	*Kolkwitzia*	*amabilis*	Graebn.	0	7	2	0	11
Fabaceae	*Laburnum*	*× watereri*	(Kirchn.)	0	3	1	0	2
Asteraceae	*Lactuca*	*bourgaei*	(Boiss.) Irish & N.Taylor	0	1	0	0	0
Asteraceae	*Lactuca*	*uralensis*	(Rouy) N.Kilian & Greuter	0	0	0	0	2
Asteraceae	*Lactuca*	*muralis*	(L.) Fresen.	0	8	0	0	0
Asteraceae	*Lactuca*	*sativa*	L.	0	0	0	0	1

Asteraceae	*Lactuca*	*serriola*	L.	1	1	0	0	20
Asteraceae	*Lactuca*	*tatarica*	(L.) C.A.Mey.	0	1	0	0	0
Asteraceae	*Lactuca*	*virosa*	L.	1	0	0	0	11
Lythraceae	*Lagerstroemia*	*indica*	L.	0	2	0	0	1
Lythraceae	*Lagerstroemia*	*limii*	Merr.	0	1	0	0	0
Lamiaceae	Not identified	Not identified		1	0	0	0	0
Lamiaceae	*Lamium*	*album*	L.	2	5	5	7	1
Lamiaceae	*Lamium*	*amplexicaule*	L.	0	0	2	1	10
Lamiaceae	*Lamium*	*galeobdolon*	(L.) L.	0	0	0	0	10
Lamiaceae	*Lamium*	*hybridum*	Vill.	0	1	0	2	0
Lamiaceae	*Lamium*	*maculatum*	(L.) L.	0	4	0	1	1
Lamiaceae	*Lamium*	*purpureum*	L.	1	2	5	0	10
Lamiaceae	*Lamium*	sp.		0	0	0	0	1
Papaveraceae	*Lamprocapnos*	*spectabilis*	(L.) Fukuhara	0	2	0	0	7
Verbenaceae	*Lantana*	*achyranthifolia*	Desf.	0	0	0	0	1
Verbenaceae	*Lantana*	*camara*	L.	1	5	0	0	1
Verbenaceae	*Lantana*	*montevidensis*	(Spreng.) Briq.	0	1	0	0	7
Verbenaceae	*Lantana*	*pastazensis*	Moldenke	0	0	0	0	1
Asteraceae	*Lapsana*	*communis*	L.	0	10	0	8	31
Apiaceae	*Laser*	*trilobum*	(L.) Borkh.	0	1	0	0	0
Apiaceae	*Laserpitium*	*gallicum*	L.	0	1	0	0	0
Apiaceae	*Laserpitium*	*siler*	L.	0	1	0	0	0
Fabaceae	*Lathyrus*	*aphaca*	L.	0	2	0	0	0
Fabaceae	*Lathyrus*	*latifolius*	L.	0	1	1	0	9
Fabaceae	*Lathyrus*	*linifolius*	(Reichard) Bassler	0	0	0	0	1

Fabaceae	*Lathyrus*	*pratensis*	L.	1	2		1	7	15
Fabaceae	*Lathyrus*	*rotundifolius*	Willd.	0	1		0	0	0
Fabaceae	*Lathyrus*	*sativus*	L.	0	1		0	0	2
Fabaceae	*Lathyrus*	*sylvestris*	L.	0	1		0	1	0
Fabaceae	*Lathyrus*	*vernus*	(L.) Bernh.	0	0		0	0	2
Lauraceae	*Laurus*	*nobilis*	L.	0	1		0	0	1
Lamiaceae	*Lavandula*	*angustifolia*	Mill.	8	7		4	0	23
Lamiaceae	*Lavandula*	*latifolia*	Medik.	0	1		0	0	0
Lamiaceae	*Lavandula*	*stoechas*	L.	1	2		0	0	9
Malvaceae	*Lavatera*	*olbia*	L.	0	1		0	0	0
Malvaceae	*Lavatera*	*thuringiaca*	L.	0	1		0	0	0
Brassicaceae	*Ledidium*	*draba*	L.	0	0		0	0	1
Ericaceae	*Ledum*	*palustre*	L.	0	0		0	0	1
Campanulaceae	*Legousia*	*speculum-veneris*	(L.) Chaix	0	0		0	0	1
Fabaceae	*Lens*	*culinaris*	Medik.	0	0		0	0	1
Asteraceae	*Leontodon*	*hispidus*	L.	5	0		2	11	27
Asteraceae	*Leontopodium*	*alpinum*	Cass.	0	0		0	0	2
Lamiaceae	*Leonurus*	*cardiaca*	L.	0	1		0	1	3
Lamiaceae	*Leonurus*	*macranthus*	Maxim.	0	0		0	0	1
Brassicaceae	*Cardaria*	*draba*	L.	0	1		0	0	0
Brassicaceae	*Lepidium*	*latifolium*	L.	0	1		0	0	0
Brassicaceae	*Lepidium*	*virginicum*	L.	0	0		0	0	6
Fabaceae	*Lespedeza*	*thunbergii*	(DC.) Nakai	0	1		0	0	0
Asteraceae	*Leucanthemum*	*adustum*	(W. D. J. Koch) Gremli	0	1		0	0	0
Asteraceae	*Leucanthemum*	*gaudinii*	Dalla Torre	0	0		0	0	1

Asteraceae	*Leucanthemum*	*vulgare* aggr.		3	8	0	12	26
Apiaceae	*Levisticum*	*officinale*	W. D. J. Koch	0	1	0	0	0
Asteraceae	*Liatris*	*spicata*	(L.) Willd.	0	1	0	0	1
Iridaceae	*Libertia*	*chilensis*	(Molina) Gunckel	0	1	0	0	0
Asteraceae	*Ligularia*	*dentata*	(A.Gray) Hara	0	0	0	0	1
Asteraceae	*Ligularia*	*przewalskii*	(Maxim.) Diels	0	0	0	0	1
Oleaceae	*Ligustrum*	*delavayanum*	Har.	0	1	0	0	0
Oleaceae	*Ligustrum*	*ibota*	Siebold	0	0	0	0	1
Oleaceae	*Ligustrum*	*japonicum*	Thunb.	1	0	0	0	2
Oleaceae	*Ligustrum*	*lucidum*	W. T. Aiton	0	1	0	0	0
Oleaceae	*Ligustrum*	*ovalifolium*	Hassk.	3	1	0	0	1
Oleaceae	*Ligustrum*	*sinense*	Lour.	0	1	0	0	0
Oleaceae	*Ligustrum*	*vulgare*	L.	3	6	1	1	20
Liliaceae	*Lilium*	*bulbiferum*	L.	0	1	0	0	0
Liliaceae	*Lilium*	*candidum*	L.	0	1	0	0	1
Liliaceae	*Lilium*	*martagon*	L.	0	0	0	0	2
Liliaceae	*Lilium*	*regale*	E. H. Wilson	0	1	0	0	0
Liliaceae	*Lilium*	sp.		1	1	1	0	3
Plumbaginaceae	*Limonium*	*platyphyllum*	Lincz.	0	1	0	0	0
Plumbaginaceae	*Limonium*	*sinuatum*	(L.) Mill.	0	1	0	0	0
Plantaginaceae	*Linaria*	*incarnata*	(Vent.) Spreng.	1	0	0	0	0
Plantaginaceae	*Linaria*	*maroccana*	Hook. f.	1	0	0	0	3
Plantaginaceae	*Linaria*	*purpurea*	(L.) Mill.	0	1	0	0	6
Plantaginaceae	*Linaria*	*repens*	(L.) Mill.	0	1	0	0	0
Plantaginaceae	*Linaria*	*vulgaris*	Mill.	1	1	1	1	12

Boraginaceae	*Lindelofia*	*longiflora*	(Benth.) Baill.	0	1	0	0	0
Linderniaceae	*Lindernia*	*grandiflora*	Nutt.	0	1	0	0	0
Linaceae	*Linum*	*bienne*	Mill.	0	0	0	0	2
Linaceae	*Linum*	*dolomiticum*	Borbás	0	0	0	0	1
Linaceae	*Linum*	*flavum*	L.	0	0	0	0	1
Linaceae	*Linum*	*grandiflorum*	Desf.	0	1	0	0	7
Linaceae	*Linum*	*narbonense*	L.	0	0	0	0	1
Linaceae	*Linum*	*perenne*	L.	0	1	0	0	1
Linaceae	*Linum*	*usitatissimum*	L.	0	0	1	0	7
Magnoliaceae	*Liriodendron*	*tulipifera*	L.	0	0	0	0	3
Asparagaceae	*Liriope*	*muscari*	(Decne.) L. H. Bailey	0	1	0	0	0
Boraginaceae	*Lithodora*	*zahnii*	(Heldr. ex Halácsy) I. M. Johnst.	0	1	0	0	0
Boraginaceae	*Lithospermum*	*erythrorhizon*	Siebold & Zucc.	0	0	0	0	1
Boraginaceae	*Lithospermum*	*officinale*	L.	0	1	0	0	1
Campanulaceae	*Lobelia*	*cardinalis*	L.	0	2	0	0	0
Campanulaceae	*Lobelia*	*erinus*	L.	4	2	0	2	10
Campanulaceae	*Lobelia*	*laxiflora*	Kunth	0	1	0	0	0
Campanulaceae	*Lobelia*	*pedunculata*	R.Br.	0	1	0	0	0
Campanulaceae	*Lobelia*	*siphilitica*	L.	0	1	0	0	1
Brassicaceae	*Lobularia*	*maritima*	(L.) Desv.	3	4	0	0	8
Caprifoliaceae	*Lomelosia*	*graminifolia*	(L.) Greuter & Burdet	0	1	0	0	1
Caprifoliaceae	*Lonicera*	*angustifolia*	Wall. ex. DC	0	1	0	0	0

Caprifoliaceae	*Lonicera*	*caprifolium*	L.	0	2	0	1	0
Caprifoliaceae	*Lonicera*	*etrusca*	Santi	0	1	0	0	0
Caprifoliaceae	*Lonicera*	*implexa*	Aiton	0	1	2	0	2
Caprifoliaceae	*Lonicera*	*japonica*	Thunb.	1	1	1	0	2
Caprifoliaceae	*Lonicera*	*nitida*	E. H. Wilsom	0	0	0	0	8
Caprifoliaceae	*Lonicera*	*periclymenum*	L.	0	1	0	0	1
Caprifoliaceae	*Lonicera*	*pileata*	Oliv.	0	0	0	0	14
Caprifoliaceae	*Lonicera*	*tatarica*	L.	0	0	0	0	1
Caprifoliaceae	*Lonicera*	*× purpusii*	Rehder	0	0	0	0	1
Caprifoliaceae	*Lonicera*	*xylosteum*	L.	0	1	0	0	12
Hamamelidaceae	*Loropetalum*	*chinense*	(R. Br.) Oliv.	0	0	0	0	1
Fabaceae	*Lotus*	*corniculatus*	L.	5	6	9	2	28
Fabaceae	*Lotus*	*maritimus*	L.	0	1	0	0	1
Fabaceae	*Lotus*	*pedunculatus*	Cav.	0	1	2	0	2
Fabaceae	*Lotus*	*tetragonolobus*	L.	0	0	0	0	1
Onagraceae	*Ludwigia*	*grandiflora*	(Michx.) Greuter & Burdet	0	1	0	0	0
Cucurbitaceae	*Luffa*	*cylindrica*	(L.) M.Roem.	0	0	0	0	1
Brassicaceae	*Lunaria*	*annua*	L.	0	4	1	0	2
Brassicaceae	*Lunaria*	*rediviva*	L.	0	1	0	0	3
Fabaceae	*Lupinus*	*albus*	L.	0	0	0	0	1
Fabaceae	*Lupinus*	*luteus*	L.	0	1	0	0	0
Fabaceae	*Lupinus*	*polyphyllus*	Lindl.	0	1	0	3	0
Fabaceae	*Lupinus*	*semperflorens*	Benth.	0	0	0	0	1
Fabaceae	*Lupinus*	sp.		0	0	0	0	1
Solanaceae	*Lycianthes*	*rantonnettii*	(Carrière) Bitter	0	0	0	0	2

Solanaceae	*Lycium*	*barbarum*	L.	0	1	1	0	1
Solanaceae	*Lycopersicon*	*esculentum*	Mill.	1	1	0	2	10
Lamiaceae	*Lycopus*	*europaeus*	L.	1	0	0	0	1
Araceae	*Lysichiton*	*americanus*	Hultèn & H. St. John	0	0	0	0	1
Araceae	*Lysichiton*	*camtschatcensis*	(L.) Schott	0	0	0	0	1
Primulaceae	*Lysimachia*	*atropurpurea*	L.	0	0	0	0	1
Primulaceae	*Lysimachia*	*ciliata*	L.	0	1	0	0	0
Primulaceae	*Lysimachia*	*clethroides*	Duby	0	1	0	0	3
Primulaceae	*Lysimachia*	*deltoidea*	Wight	0	1	0	0	0
Primulaceae	*Lysimachia*	*ephemerum*	L.	0	1	0	0	1
Primulaceae	*Lysimachia*	*maritima*	(L.) Galasso, Banfi & Soldano	0	1	0	0	0
Primulaceae	*Lysimachia*	*nemorum*	L.	0	0	0	0	2
Primulaceae	*Lysimachia*	*nummularia*	L.	1	2	0	3	12
Primulaceae	*Lysimachia*	*punctata*	L.	2	5	0	0	15
Primulaceae	*Lysimachia*	*thyrsiflora*	L.	0	0	0	0	2
Primulaceae	*Lysimachia*	*vulgaris*	L.	1	2	0	0	8
Lythraceae	*Lythrum*	*hyssopifolia*	L.	0	1	0	0	0
Lythraceae	*Lythrum*	*salicaria*	L.	3	4	0	0	15
Asteraceae	*Madia*	*elegans*	D.Don ex Lindl.	0	2	0	0	0
Magnoliaceae	*Magnolia*	*kobus*	DC.	0	0	0	0	1
Magnoliaceae	*Magnolia*	*obovata*	Thunb.	0	0	0	0	2
Magnoliaceae	*Magnolia*	*× soulangeana*	Soul.-Bod.	0	0	0	0	5
Berberidaceae	*Mahonia*	*aquifolium*	(Pursh) Nutt.	0	0	0	0	13
Aizoaceae	*Malephora*	*crocea*	(Jacq.) Schwantes	0	1	0	0	0

Euphorbiaceae	*Mallotus*	*apelta*	(Lour.) Müll. Arg.	0	1	0	0	0
Rosaceae	*Malus*	*domestica*	Borkh.	0	0	0	1	7
Rosaceae	*Malus*	*floribunda*	Siebold ex Van Houtte	0	0	0	0	1
Rosaceae	*Malus*	*sylvestris*	(L.) Mill.	0	1	0	0	0
Rosaceae	*Malus*	*× moerlandsii*	Door.	0	0	0	0	1
Malvaceae	*Malva*	*alcea*	L.	0	6	0	0	9
Malvaceae	*Malva*	*cretica*	Cav.	2	0	1	0	1
Malvaceae	*Malva*	*moschata*	L.	0	1	0	0	10
Malvaceae	*Malva*	*neglecta*	Wallr.	0	4	3	0	0
Malvaceae	*Malva*	*stipulacea*	Cav.	0	1	0	0	0
Malvaceae	*Malva*	*sylvestris*	L.	3	8	0	1	17
Malvaceae	*Malva*	*verticillata*	L.	0	0	0	0	1
Apocynaceae	*Mandevilla*	CV		0	0	0	0	15
Asteraceae	*Mantisalca*	*salmantica*	(L.) Briq. & Cavill.	0	1	0	0	0
Lamiaceae	*Marrubium*	*vulgare*	L.	0	0	0	0	1
Asteraceae	*Matricaria*	*chamomilla*	L.	1	9	5	0	10
Asteraceae	*Matricaria*	*discoidea*	DC.	0	4	0	0	7
Brassicaceae	*Matthiola*	*incana*	(L.) R.Br.	0	0	0	0	3
Brassicaceae	*Matthiola*	*sinuata*	(L.) R.Br.	0	1	0	0	0
Brassicaceae	*Matthiola*	*tricuspidata*	(L.) R.Br.	0	1	0	0	0
Papaveraceae	*Meconopsis*	*cambrica*	(L.) Vig.	0	3	0	0	7
Fabaceae	*Medicago*	*arabica*	(L.) Huds.	0	5	0	0	1
Fabaceae	*Medicago*	*arborea*	L.	0	1	0	0	0
Fabaceae	*Medicago*	*doliata*	Carmign.	0	1	0	0	0

Fabaceae	*Medicago*	*lupulina*	L.	0	9	1	10	4
Fabaceae	*Medicago*	*marina*	L.	0	1	0	0	0
Fabaceae	*Medicago*	*minima*	(L.) L.	0	1	0	0	0
Fabaceae	*Medicago*	*sativa*	L.	0	2	2	1	15
Fabaceae	*Medicago*	*scutellata*	(L.) Mill.	0	1	0	0	0
Fabaceae	*Medicago*	*suffruticosa*	DC.	0	1	0	0	0
Fabaceae	*Medicago*	*× varia*	Martyn	0	0	0	0	1
Asteraceae	*Melampodium*	*montanum*	Benth.	0	1	0	0	0
Fabaceae	*Melilotus*	*albus*	Medik.	2	1	2	4	5
Fabaceae	*Melilotus*	*altissimus*	Thuill.	0	1	0	0	0
Fabaceae	*Melilotus*	*indicus*	(L.) All.	0	1	0	0	0
Fabaceae	*Melilotus*	*officinalis*	Lam.	0	1	1	1	2
Lamiaceae	*Melissa*	*officinalis*	L.	0	2	0	1	3
Lamiaceae	*Mentha*	*aquatica*	L.	1	1	0	0	1
Lamiaceae	*Mentha*	*cervina*	L.	0	1	0	0	0
Lamiaceae	*Mentha*	*longifolia*	(L.) Huds.	0	0	0	0	12
Lamiaceae	*Mentha*	*pulegium*	L.	0	1	0	0	1
Lamiaceae	*Mentha*	*requienii*	Benth.	0	0	0	0	1
Lamiaceae	*Mentha*	*spicata* aggr.		1	2	0	0	9
Lamiaceae	*Mentha*	*suaveolens*	Ehrh.	0	1	0	0	0
Lamiaceae	*Mentha*	*× piperita*	L.	0	1	0	0	0
Lamiaceae	*Mentha*	*× verticillata*	L.	0	0	0	1	0
Menyanthaceae	*Menyanthes*	*trifoliata*	L.	0	0	0	0	1
Boraginaceae	*Mertensia*	*paniculata*	(Aiton) G. Don	0	1	0	0	0
Rosaceae	*Mespilus*	*germanica*	L.	0	1	0	0	1

Apocynaceae	*Metastelma*	*strictum*	Gleason & Moldenke	0	0	0	0	1
Phrymaceae	*Mimulus*	*cardinalis*	Douglas ex Benth.	0	0	0	0	1
Phrymaceae	*Mimulus*	*guttatus*	DC.	0	2	0	0	2
Caryophyllaceae	*Minuartia*	*labillardierei*	Briq.	0	1	0	0	0
Caryophyllaceae	*Minuartia*	*laricifolia*	(L.) Schinz & Thell.	0	1	0	0	0
Caryophyllaceae	*Minuartia*	*stellata*	Petitm.	0	1	0	0	0
Caryophyllaceae	*Minuartia*	*verna*	(L.) Hiern	0	1	0	0	0
Nyctaginaceae	*Mirabilis*	*jalapa*	L.	0	1	0	0	2
Saxifragaceae	*Mitella*	sp.		0	0	0	0	1
Boraginaceae	*Moltkia*	*suffruticosa*	(L.) Hegi	0	1	0	0	0
Lamiaceae	*Monarda*	*didyma*	L.	0	0	0	0	2
Lamiaceae	*Monarda*	*fistulosa*	L.	0	1	0	0	1
Lamiaceae	*Monarda*	*punctata*	L.	0	0	0	0	1
Brassicaceae	*Moricandia*	*arvensis*	(L.) DC.	0	1	0	0	0
Caprifoliaceae	*Morina*	*longifolia*	Wall. ex DC.	0	0	0	0	1
Polygonaceae	*Muehlenbeckia*	*complexa*	Meisn.	0	0	0	0	1
Asparagaceae	*Muscari*	sp.		0	0	2	5	8
Asparagaceae	*Muscari*	*comosum*	(L.) Mill.	0	1	0	0	0
Asparagaceae	*Muscari*	*neglectum* aggr.		0	0	2	5	9
Asteraceae	*Mycelis*	*muralis*	(L.) Dumort.	2	4	0	0	20
Boraginaceae	*Myosotis*	*alpestris*	F. W. Schmidt	0	0	0	0	1
Boraginaceae	*Myosotis*	*arvensis*	Hill	5	5	5	12	12
Boraginaceae	*Myosotis*	*discolor*	Pers.	0	2	0	0	0
Boraginaceae	*Myosotis*	*ramosissima*	Rochel	0	0	0	0	7

Boraginaceae	*Myosotis*	*rehsteineri*	Wartm.	0	0	0	0	1
Boraginaceae	*Myosotis*	*scorpioides* aggr.	L.	0	2	0	1	4
Boraginaceae	*Myosotis*	*sylvatica*	Hoffm.	2	4	1	1	17
Haloragaceae	*Myriophyllum*	*aquaticum*	(Vell.) Verdc.	0	1	0	0	0
Berberidaceae	*Nandina*	*domestica*	Thunb.	0	1	0	0	2
Amaryllidaceae	*Narcissus*	*poëticus*	L.	0	0	0	3	1
Amaryllidaceae	*Narcissus*	sp.		0	1	0	0	1
Amaryllidaceae	*Narcissus*	*pseudonrcissus*	L.	0	0	0	0	11
Nartheciaceae	*Narthecium*	*ossifragum*	(L.) Huds.	0	0	0	0	1
Brassicaceae	*Nasturtium*	*officinale*	R. Br.	0	2	0	0	1
Scrophulariaceae	*Nemesia*	*floribunda*	Lehm.	0	1	0	0	0
Boraginaceae	*Nemophila*	*maculata*	Benth. ex Lindl.	0	1	0	0	0
Lamiaceae	*Nepeta*	*argolica*	Bory & Chaub.	0	1	0	0	0
Lamiaceae	*Nepeta*	*cataria*	L.	2	1	0	0	2
Lamiaceae	*Nepeta*	*grandiflora*	M.Bieb.	0	1	0	0	0
Lamiaceae	*Nepeta*	*nepetella*	L.	0	1	0	0	0
Lamiaceae	*Nepeta*	*nuda*	L.	0	1	0	0	0
Lamiaceae	*Nepeta*	*racemosa*	Lam.	0	1	0	0	0
Lamiaceae	*Nepeta*	sp.		0	0	0	0	1
Lamiaceae	*Nepeta*	*tenuifolia*	Benth.	0	0	0	0	1
Lamiaceae	*Nepeta*	*× faasenii*	Bergmans ex Stearn	0	0	0	0	3
Apocynaceae	*Nerium*	*oleander*	L.	1	4	0	0	10
Solanaceae	*Nicandra*	*physalodes*	(L.) Gaertn.	0	0	0	0	1
Solanaceae	*Nicotiana*	*alata*	Link & Otto	0	1	0	0	0
Solanaceae	*Nicotiana*	*glauca*	Graham	0	1	0	0	0

Solanaceae	*Nicotiana*	*langsdorffii*	Weinmann	0	1	0	0	0
Solanaceae	*Nicotiana*	*quadrivalvis*	Pursh	1	0	0	0	0
Solanaceae	*Nicotiana*	*rustica*	L.	0	0	0	0	2
Solanaceae	*Nicotiana*	*tabacum*	L.	0	2	0	0	1
Ranunculaceae	*Nigella*	*damascena*	L.	0	2	0	0	9
Ranunculaceae	*Nigella*	*sativa*	L.	0	0	0	0	1
Amaryllidaceae	*Nothoscordum*	*gracile*	(Aiton) Stearn	0	1	0	0	0
Asteraceae	*Notobasis*	*syriaca*	(L.) Cass.	0	1	0	0	0
Nymphaeaceae	*Nuphar*	*advena*	(Aiton) W. T. Aiton	0	0	0	0	1
Nymphaeaceae	*Nuphar*	*lutea*	(L.) Sm.	0	3	1	1	0
Nymphaeaceae	*Nuphar*	sp.		0	0	0	0	1
Nymphaeaceae	*Nymphaea*	*alba*	L.	1	3	0	1	4
Nymphaeaceae	*Nymphaea*	*candida*	C. Presl	0	0	0	0	1
Nymphaeaceae	*Nymphaea*	*mexicana*	Zucc.	0	0	0	0	1
Nymphaeaceae	*Nymphaea*	*odorata*	Aiton	0	0	0	0	1
Nymphaeaceae	*Nymphaea*	sp.		0	1	0	0	4
Nymphaeaceae	*Nymphaea*	*tetragona*	Georgi	0	0	0	0	1
Menyanthaceae	*Nymphoides*	*peltata*	(S. G. Gmel.) Kuntze	0	1	0	0	1
Caryophyllaceae	*Oberna*	*multifida*	Ikonn.	0	1	0	0	0
Lamiaceae	*Ocimum*	*basilicum*	L.	0	1	0	0	5
Lamiaceae	*Ocimum*	*gratissimum*	L.	0	0	0	0	1
Lamiaceae	*Ocimum*	*kilimandscharicum*	Gürke	0	0	0	0	1
Lamiaceae	*Ocimum*	sp.		0	0	0	0	1
Apiaceae	*Oenanthe*	*crocata*	L.	0	2	0	0	0

Apiaceae	*Oenanthe*	*fistulosa*	L.	0	0	0	0	1
Apiaceae	*Oenanthe*	*sarmentosa*	C. Presl ex DC.	0	0	0	0	1
Apiaceae	*Oenanthe*	*silaifolia*	M.Bieb.	0	1	0	0	0
Onagraceae	*Oenothera*	*biennis* agr.	L.	0	1	2	2	15
Onagraceae	*Oenothera*	*fruticosa*	L.	0	1	0	0	1
Onagraceae	*Oenothera*	*magallanica*	Phil.	0	1	0	0	0
Onagraceae	*Oenothera*	*parviflora*	L.	0	0	0	1	2
Onagraceae	*Oenothera*	*rosea*	L'Hér. ex Aiton	0	1	0	0	0
Onagraceae	*Oenothera*	sp.		0	0	0	0	3
Onagraceae	*Oenothera*	*speciosa*	Nutt.	0	1	0	0	0
Fabaceae	*Onobrychis*	*viciifolia*	Scop.	0	1	0	0	10
Fabaceae	*Ononis*	*cristata*	Mill.	0	0	0	0	1
Fabaceae	*Ononis*	*natrix*	L.	0	1	0	0	0
Fabaceae	*Ononis*	*repens*	L.	0	1	0	0	0
Fabaceae	*Ononis*	*spinosa*	L.	0	1	0	0	1
Fabaceae	*Ononis*	*viscosa*	L.	0	1	0	0	0
Boraginaceae	*Onosma*	*stellulata*	Waldst. & Kit.	0	1	0	0	0
Asparagaceae	*Ophiopogon*	*planiscapus*	Nakai	0	1	0	0	0
Lamiaceae	*Origanum*	*majorana*	L.	0	0	0	0	1
Lamiaceae	*Origanum*	*vulgare*	L.	1	2	0	1	22
Apiaceae	*Orlaya*	*grandiflora*	(L.) Hoffm.	0	1	0	0	0
Asparagaceae	*Ornithogalum*	*sigmoideum*	Freyn & Sint.	0	1	0	0	0
Asparagaceae	*Ornithogalum*	*umbellatum* aggr.	L.	0	1	3	1	5
Fabaceae	*Ornithopus*	*perpusillus*	L.	2	0	0	0	0
Fabaceae	*Ornithopus*	*sativus*	Brot.	0	1	0	0	0
Orobanchaceae	*Orobanche*	*hederae*	Duby	0	0	0	0	1

Orobanchaceae	*Orobanche*	*picridis*	F. W. Schultz	0	1		0	0	0
Araceae	*Orontium*	*aquaticum*	L.	0	0		0	0	1
Oleaceae	*Osmanthus*	*heterophyllus*	(G. Don) P. S. Green	0	1		0	0	0
Asteraceae	*Osteospermum*	*ecklonis*	(DC.) Norl.	2	1		1	0	6
Asteraceae	*Osteospermum*	*fruticosum*	(L.) Norl.	0	1		0	0	0
Asteraceae	*Otanthus*	*maritimus*	(L.) Hoffmanns. & Link	0	1		0	0	0
Oxalidaceae	*Oxalis*	*acetosella*	L.	0	0		0	2	2
Oxalidaceae	*Oxalis*	*articulata*	Savigny	1	0		0	0	1
Oxalidaceae	*Oxalis*	*corniculata*	L.	4	6		1	0	17
Oxalidaceae	*Oxalis*	*exilis*	A. Cunn.	0	1		0	0	0
Oxalidaceae	*Oxalis*	*floribunda*	Lehm.	0	1		0	0	0
Oxalidaceae	*Oxalis*	*latifolia*	Kunth	0	3		0	0	0
Oxalidaceae	*Oxalis*	*stricta*	L.	0	0		1	0	19
Oxalidaceae	*Oxalis*	*triangularis*	A. St.-Hil.	0	0		0	0	1
Paeoniaceae	*Paeonia*	*broteri*	Boiss. & Reut.	0	1		0	0	0
Paeoniaceae	*Paeonia*	*daurica subsp. mlokosewitschii*	(Lomakin) D. Y. Hong	0	0		0	0	1
Paeoniaceae	*Paeonia*	*officinalis*	L.	0	1		0	1	1
Paeoniaceae	*Paeonia*	lactiflora	Pall.	0	0		3	4	8
Asteraceae	*Pallenis*	*maritima*	(L.) Greuter	0	1		0	0	0
Asteraceae	*Pallenis*	*spinosa*	(L.) Cass.	0	1		0	0	0
Amaryllidaceae	*Pancratium*	*illyricum*	L.	0	1		0	0	0
Bignoniaceae	*Pandorea*	*jasminoides*	(Lindl.) K.Schum.	0	0		0	0	1
Papaveraceae	*Papaver*	*croceum*	Ledeb.	0	0		0	0	1

Papaveraceae	*Papaver*	*dubium*	L.	0	2		0	0	0
Papaveraceae	*Papaver*	*hybridum*	L.	0	1		0	0	0
Papaveraceae	*Papaver*	*nudicaule*	L.	0	1		0	0	0
Papaveraceae	*Papaver*	*orientale*	L.	0	1		0	0	0
Papaveraceae	*Papaver*	*rhoeas*	L.	3	6		1	1	20
Papaveraceae	*Papaver*	*somniferum*	L.	0	1		1	1	4
Plantaginaceae	*Parahebe*	*catarractae*	(G. Forst.) W. R. B. Oliv.	0	1		0	0	0
Boraginaceae	*Pardoglossum*	*cheirifolium*	(L.) E. Barbier & Mathez	0	1		0	0	0
Urticaceae	*Parietaria*	*judaica*	L.	0	11		0	0	0
Celastraceae	*Parnassia*	*palustris*	L.	0	0		0	0	1
Caryophyllaceae	*Paronychia*	*capitata*	(L.) Lam.	0	0		0	0	1
Hamamelidaceae	*Parrotia*	*persica*	C.A.Mey	0	0		0	0	1
Vitaceae	*Parthenocissus*	*inserta*	(A. Kern.) Fritsch	0	0		0	0	3
Vitaceae	*Parthenocissus*	*quinquefolia*	(L.) Planch.	0	0		0	0	1
Passifloraceae	*Passiflora*	*caerulea*	L.	0	1		0	0	0
Passifloraceae	*Passiflora*	*edulis*	Sims	0	0		0	0	1
Apiaceae	*Pastinaca*	*sativa*	L.	0	1		0	6	4
Caprifoliaceae	*Patrinia*	*triloba*	Miq.	0	0		0	0	1
Paulowniaceae	*Paulownia*	*tomentosa*	(Thunb.) Steud.	2	0		0	0	0
Orobanchaceae	*Pedicularis*	*sceptrum-carolinum*	L.	0	0		0	0	1
Geraniaceae	*Pelargonium*	*endlicherianum*	Fenzl	0	1		0	0	0
Geraniaceae	*Pelargonium*	*graveolens*	L'Hér.	0	0		0	0	1
Geraniaceae	*Pelargonium*	CV		7	8		6	1	18
Geraniaceae	*Pelargonium*	*tomentellum*	DC.	0	1		0	0	0

Geraniaceae	*Pelargonium*	*× hortorum*	L.H. Bailey	0	0	0	0	2
Geraniaceae	*Pelargonium*	*zonale*	(L.) L'Hér. ex Aiton	0	4	6	0	0
Plantaginaceae	*Penstemon*	*barbatus*	(Cav.) Roth	0	1	0	0	1
Plantaginaceae	*Penstemon*	*digitalis*	Nutt. ex Sims	0	1	0	0	1
Plantaginaceae	*Penstemon*	*kunthii*	G. Don	0	1	0	0	0
Plantaginaceae	*Penstemon*	*labrosus*	(A. Gray) Mast. ex Hook. f.	0	1	0	0	0
Plantaginaceae	*Penstemon*	*serrulatus*	Menzies ex Sm.	0	1	0	0	0
Plantaginaceae	*Penstemon*	*smallii*	A. Heller	0	1	0	0	0
Plantaginaceae	*Penstemon*	*triflorus*	A. Heller	1	0	0	0	0
Boraginaceae	*Pentaglottis*	*sempervirens*	(L.) Tausch ex L.H.Bailey	0	1	0	0	0
Penthoraceae	*Penthorum*	*sedoides*	L.	0	0	0	0	1
Lamiaceae	*Perovskia*	*atriplicifolia*	Benth.	1	3	0	0	3
Polygonaceae	*Polygonum*	*bistorta*	L.	2	1	0	0	6
Polygonaceae	*Polygonum*	*campanulatum*	Hook. F.	0	1	0	0	0
Polygonaceae	*Polygonum*	*capitatum*	D. Don	0	0+E1472:F1472	0	0	1
Polygonaceae	*Polygonum*	*persicaria*	L.	1	1	0	0	0
Polygonaceae	*Polygonum*	*orientale*	L.	0	0	0	0	2
Polygonaceae	*Polygonum*	*virginianum*	L.	0	1	0	0	0
Caryophyllaceae	*Petrorhagia*	*prolifera*	(L.) P. W. Ball & Heywood	0	0	0	0	5
Caryophyllaceae	*Petrorhagia*	*saxifraga*	(L.) Link	0	1	0	0	12

Apiaceae	*Petroselinum*	*crispum*	(Mill.) Fuss	0	0	0	0	1
Solanaceae	*Petunia*	*axillaris*	(Lam.) Britton, Sterns & Poggenb.	3	2	0	0	0
Solanaceae	*Petunia*	*× hybrida*	(Hook.) Villm.	5	3	6	3	16
Apiaceae	*Peucedanum*	*cervaria*	(L.) Lapeyr.	0	0	0	0	1
Boraginaceae	*Phacelia*	*tanacetifolia*	Benth.	0	2	0	0	6
Orchidaceae	*Phalaenopsis*	sp.		1	0	0	0	1
Fabaceae	*Phaseolus*	*coccineus*	L.	0	1	0	0	2
Fabaceae	*Phaseolus*	*vulgaris*	L.	0	0	0	0	1
Hydrangeaceae	*Philadelphus*	*coronarius*	L.	3	6	0	4	1
Hydrangeaceae	*Philadelphus*	*schrenkii*	Rupr.	0	1	0	0	0
Hydrangeaceae	*Philadelphus*	*subcanus*	Koehne	0	1	0	0	0
Oleaceae	*Phillyrea*	*latifolia*	L.	0	0	0	0	1
Lamiaceae	*Phlomis*	*fruticosa*	L.	1	4	0	0	2
Lamiaceae	*Phlomis*	*herba-venti*	L.	0	1	0	0	0
Lamiaceae	*Phlomis*	*lychnitis*	L.	0	1	0	0	0
Lamiaceae	*Phlomis*	*russeliana*	(Sims) Lag. ex Benth.	0	0	0	0	3
Lamiaceae	*Phlomoides*	*laciniata*	(L.) Kamelin & Makhm.	0	1	0	0	0
Polemoniaceae	*Phlox*	drummondii	Hook.	0	0	0	0	4
Polemoniaceae	*Phlox*	paniculata	L.	0	1	0	2	19
Polemoniaceae	*Phlox*	subulata	L.	0	1	0	0	2
Rubiaceae	*Phuopsis*	*stylosa*	Jacks.	0	1	0	0	1
Scrophulariaceae	*Phygelius*	*capensis*	E. Mey. ex Benth.	0	1	0	0	0

Verbenaceae	*Phyla*	*canescens*	(Kunth) Greene	0	1	0	0	0
Verbenaceae	*Phyla*	*nodiflora*	(L.) Greene	0	1	0	0	0
Verbenaceae	*Phyla*	*scaberrima*	(Juss. ex Pers.) Moldenke	0	0	0	0	1
Solanaceae	*Physalis*	*alkekengi*	L.	0	1	0	0	1
Solanaceae	*Physalis*	*ixocarpa*	Brot. ex Hornem.	0	0	0	0	1
Solanaceae	*Physalis*	*philadelphica*	Lam.	0	1	0	0	0
Rosaceae	*Physocarpus*	*opulifolius*	(L.) Maxim.	0	1	3	0	0
Solanaceae	*Physochlaina*	*orientalis*	G.Don	0	1	0	0	0
Lamiaceae	*Physostegia*	*viginiana*	(L.) Benth.	0	2	0	0	1
Campanulaceae	*Phyteuma*	*scheuchzeri*	All.	0	1	0	0	0
Campanulaceae	*Phyteuma*	*spicatum*	L.	0	0	0	0	3
Phytolaccaceae	*Phytolacca*	*americana*	L.	1	3	0	0	0
Phytolaccaceae	*Phytolacca*	*rivinoides*	Kunth & C. D. Bouché	1	0	0	0	0
Asteraceae	*Picris*	*hieracioides*	L.	3	5	0	0	11
Ericaceae	*Pieris*	*japonica*	(Thunb.) D. Don ex G. Don	0	0	0	0	1
Apiaceae	*Pimpinella*	*anisum*	L.	0	0	0	0	1
Apiaceae	*Pimpinella*	*major*	(L.) Huds.	0	0	0	0	1
Lentibulariaceae	*Pinguicula*	*agnata*	Casper	0	0	0	0	1
Lentibulariaceae	*Pinguicula*	*gigantea*	Luhrs	0	0	0	0	1
Lentibulariaceae	*Pinguicula*	*moctezumae*	Zamudio & R. Z. Ortega	0	0	0	0	1

Pittosporaceae	*Pittosporum*	*tobira*	(Thunb.) W. T. Aiton	0	2		0	0	0
Asteraceae	*Plagius*	*flosculosus*	(L.) S. Alavi & V. H. Heywood	0	1		0	0	0
Plantaginaceae	*Plantago*	*coronopus*	L.	0	0		0	0	7
Plantaginaceae	*Plantago*	*lanceolata*	L.	8	2		10	1	29
Plantaginaceae	*Plantago*	*major*	L.	0	2		0	0	18
Plantaginaceae	*Plantago*	*media*	L.	0	1		0	3	16
Orchidaceae	*Platanthera*	*bifolia*	(L.) Rich.	0	0		0	1	0
Campanulaceae	*Platycodon*	*grandiflorus*	(Jacq.) A. DC.	1	0		0	0	4
Plumbaginaceae	*Plumbago*	*auriculata*	Lam.	0	1		0	0	1
Plumbaginaceae	*Plumbago*	*zeylanica*	L.	0	0		0	0	1
Apocynaceae	*Plumeria*	*pudica*	Jacq.	1	0		0	0	0
Bignoniaceae	*Podranea*	*ricasoliana*	(Tanfani) Sprague	0	1		0	0	1
Lamiaceae	*Pogostemon*	*cablin*	(Blanco) Benth.	0	0		0	0	1
Cleomaceae	*Polanisia*	*dodecandra*	(L.) DC.	0	1		0	0	0
Polemoniaceae	*Polemonium*	*caeruleum*	L.	0	1		0	0	1
Polemoniaceae	*Polemonium*	*chinense*	(Brand) Brand	0	1		0	0	0
Polemoniaceae	*Polemonium*	*foliosissimum*	A. Gray	0	0		0	0	1
Polemoniaceae	*Polemonium*	*pauciflorum*	S. Watson	0	1		0	0	0
Asparagaceae	*Polianthes*	*tuberosa*	L.	0	1		0	0	0
Asparagaceae	*Polygonatum*	*multiflorum*	(L.) All.	0	1		0	2	6
Asparagaceae	*Polygonatum*	*odoratum*	(Mill.) Druce	0	0		1	0	2
Asparagaceae	*Polygonatum*	*verticillatum*	(L.) All.	0	0		0	0	2
Polygonaceae	*Polygonum*	*affine*	D. Don	0	1		0	0	0

Polygonaceae	*Polygonum*	*aviculare* aggr.		3	1	2	2	1
Polygonaceae	*Polygonum*	*microcephalum*	D. Don	0	1	0	0	0
Polygonaceae	*Polygonum*	*minus*	Huds.	0	1	0	0	0
Polygonaceae	*Polygonum*	*odoratum*	Lour.	0	0	0	0	1
Polygonaceae	*Polygonum*	*scoparium*	Req. ex Loisel.	0	1	0	0	0
Polygonaceae	*Polygonum*	*viviparum*	L.	0	1	0	0	0
Boraginaceae	*Pontechium*	*maculatum*	(L.) Böhle & Hilger	0	1	0	0	0
Pontederiaceae	*Pontederia*	*cordata*	L.	0	1	0	0	1
Salicaceae	*Populus*	*alba*	L.	0	0	0	0	1
Salicaceae	*Populus*	*nigra*	L.	0	0	0	0	2
Salicaceae	*Populus*	*× canadensis*	Moench	0	0	0	0	1
Salicaceae	*Populus*	*× canescens*	(Aiton) Sm.	0	0	0	0	1
Portulacaceae	*Portulaca*	*grandiflora*	Hook.	2	0	0	0	0
Portulacaceae	*Portulaca*	*oleracea*	L.	0	0	1	0	9
Portulacaceae	*Portulaca*	sp.		2	0	0	0	0
Portulacaceae	*Portulaca*	*umbraticola*	Kunth	1	0	0	0	0
Rosaceae	*Potentilla*	*anserina*	L.	0	1	3	11	3
Rosaceae	*Potentilla*	*argentea*	L.	0	1	1	4	1
Rosaceae	*Potentilla*	*aurea*	L.	0	0	0	0	1
Rosaceae	*Potentilla*	*chrysantha*	(Zoll. & Moritzi) Trevir.	0	1	0	0	0
Rosaceae	*Potentilla*	*verna*	L.	0	1	2	2	13
Rosaceae	*Potentilla*	*eriocarpa*	Wall. ex Lehm.	0	0	0	0	1
Rosaceae	*Potentilla*	*fruticosa*	L.	2	2	3	2	13
Rosaceae	*Potentilla*	*hirta*	L.	0	1	0	0	0

Rosaceae	*Potentilla*	*megalantha*	Takeda	0	0	0	0	2
Rosaceae	*Potentilla*	*nepalensis*	Hook.	0	0	0	0	1
Rosaceae	*Potentilla*	*norvegica*	L.	0	1	0	1	0
Rosaceae	*Potentilla*	*palustris*	(L.) Scop.	0	0	0	1	0
Rosaceae	*Potentilla*	*recta*	L.	0	1	2	1	3
Rosaceae	*Potentilla*	*reptans*	L.	1	5	6	0	29
Rosaceae	*Potentilla*	*rupestris*	L.	0	1	0	0	0
Rosaceae	*Potentilla*	sp.		0	0	0	0	1
Rosaceae	*Potentilla*	*sterilis*	(L.) Garcke	0	0	1	0	8
Rosaceae	*Potentilla*	*thuringiaca*	Link	0	1	0	0	0
Asteraceae	*Prenanthes*	*purpurea*	L.	0	1	0	0	1
Primulaceae	*Primula*	*acaulis*	(L.) L.	0	2	0	3	20
Primulaceae	*Primula*	*auricula*	L.	0	0	0	0	1
Primulaceae	*Primula*	*denticulata*	Sm	0	0	0	0	1
Primulaceae	*Primula*	*eburnea*	Balf. F. & R. E. Cooper	0	0	0	0	1
Primulaceae	*Primula*	*elatior*	(L.) L.	0	0	0	2	6
Primulaceae	*Primula*	*farinosa*	L.	0	0	0	1	1
Primulaceae	*Primula*	*frondosa*	Janka	0	1	0	0	0
Primulaceae	*Primula*	*japonica*	A. Gray	0	0	0	1	1
Primulaceae	*Primula*	*polyneura*	Franch.	0	0	0	0	1
Primulaceae	*Primula*	*pusilla*	Goldie	0	1	0	0	0
Primulaceae	*Primula*	*rosea*	Royle	0	0	0	0	3
Primulaceae	*Primula*	*spectabilis*	Tratt.	0	1	0	0	0
Primulaceae	*Primula*	*veris*	L.	0	0	0	6	4
Lamiaceae	*Prostanthera*	*cuneata*	Benth.	0	1	0	0	0

Lamiaceae	*Prostanthera*	sp.		0	1	0	0	0
Proteaceae	*Protea*	*cynaroides*	(L.) L.	0	0	0	0	1
Lamiaceae	*Prunella*	*grandiflora*	(L.) Scholler	1	1	1	0	11
Lamiaceae	*Prunella*	*laciniata*	(L.) L.	0	1	0	0	0
Lamiaceae	*Prunella*	*vulgaris*	L.	3	1	0	6	32
Rosaceae	*Prunus*	*avium*	L.	0	0	0	6	14
Rosaceae	*Prunus*	*cerasifera*	Ehrh.	0	0	0	4	1
Rosaceae	*Prunus*	*domestica*	L.	0	1	0	0	3
Rosaceae	*Prunus*	*laurocerasus*	L.	1	3	0	0	11
Rosaceae	*Prunus*	*padus*	L.	0	0	0	2	13
Rosaceae	*Prunus*	*serotina*	Ehrh.	2	0	0	0	5
Rosaceae	*Prunus*	*serrulata*	Lindl.	0	0	1	0	0
Rosaceae	*Prunus*	sp.		0	1	0	0	2
Rosaceae	*Prunus*	*spinosa*	L.	0	0	0	0	4
Asteraceae	*Centaurea*	*bella*	Trautv.	0	1	0	0	0
Papaveraceae	*Pseudofumaria*	*alba subsp. acaulis*	(Wulfen) Lidén	0	1	0	0	0
Boraginaceae	*Pseudomertensia*	*echioides*	Riedl	0	1	0	0	0
Styracaceae	*Pterostyrax*	*hispidus*	Siebold & Zucc.	0	1	0	0	0
Asteraceae	*Ptilostemon*	*casabonae*	(L.) Greuter	0	1	0	0	0
Asteraceae	*Pulicaria*	*dysenterica*	(L.) Bernh.	3	3	0	0	1
Asteraceae	*Pulicaria*	*odora*	(L.) Rchb.	0	1	0	0	0
Boraginaceae	*Pulmonaria*	*mollis* aggr.		0	0	0	0	1
Boraginaceae	*Pulmonaria*	*officinalis* aggr.	L.	0	0	0	0	4
Lythraceae	*Punica*	*granatum*	L.	0	2	0	0	1
Asparagaceae	*Puschkinia*	*scilloides*	Adams	0	0	0	0	1

Lamiaceae	*Pychnanthemum*	*virginianum*	Durand & Jackson	0	0	0	0	1
Rosaceae	*Pyracantha*	*coccinea*	M. Roem.	1	6	4	0	3
Rosaceae	*Pyrus*	*communis*	L.	0	0	0	1	3
Rosaceae	*Pyrus*	*nivalis*	Jacq.	0	0	0	0	2
Rosaceae	*Pyrus*	*pyraster*	Burgsd.	0	0	0	0	3
Rosaceae	*Pyrus*	*salicifolia*	Balb.	0	0	0	0	3
Fagaceae	*Quercus*	*ilex*	L.	0	0	0	0	1
Fagaceae	*Quercus*	*robur*	L.	1	0	0	0	7
Fagaceae	*Quercus*	*rubra*	L.	0	0	0	0	2
Gesneriaceae	*Ramonda*	*myconi*	(L.) Rchb.	0	0	0	0	1
Gesneriaceae	*Ramonda*	*× regis-ferdinandi*	Kellerer	0	0	0	0	1
Ranunculaceae	*Ranunculus*	*aconitifolius*	L.	0	0	0	0	1
Ranunculaceae	*Ranunculus*	*acris*	L.	7	1	2	11	27
Ranunculaceae	*Ranunculus*	*arvensis*	L.	0	1	0	0	13
Ranunculaceae	*Ranunculus*	*auricomus* aggr.		0	1	0	0	1
Ranunculaceae	*Ranunculus*	*bulbosus*	L.	0	3	4	0	13
Ranunculaceae	*Ranunculus*	*cassubicus*	L.	0	0	0	3	0
Ranunculaceae	*Ranunculus*	*fallax*	Sloboda	0	0	0	1	0
Ranunculaceae	*Ficaria*	*verna*	Huds.	0	0	0	7	18
Ranunculaceae	*Ranunculus*	*flammula*	L.	0	1	0	0	0
Ranunculaceae	*Ranunculus*	*glacialis*	L.	0	1	0	0	0
Ranunculaceae	*Ranunculus*	*gramineus*	L.	0	1	0	0	1
Ranunculaceae	*Ranunculus*	*japonicus*	Thunb.	0	0	0	0	1
Ranunculaceae	*Ranunculus*	*lanuginosus*	L.	0	1	0	0	0
Ranunculaceae	*Ranunculus*	*lingua*	L.	0	1	0	0	0

Ranunculaceae	*Ranunculus*	*paludosus*	Poir.	0	1	0	0	0
Ranunculaceae	*Ranunculus*	*repens*	L.	7	12	10	12	31
Ranunculaceae	*Ranunculus*	*reptans*	L.	0	0	0	0	1
Ranunculaceae	*Ranunculus*	*sardous*	Crantz	0	1	0	0	0
Ranunculaceae	*Ranunculus*	*sceleratus*	L.	0	2	0	1	1
Brassicaceae	*Raphanus*	*raphanistrum*	L.	1	0	0	0	0
Brassicaceae	*Raphanus*	*sativus*	L.	0	1	0	0	2
Brassicaceae	*Rapistrum*	*perenne*	(L.) All.	0	0	0	0	1
Brassicaceae	*Rapistrum*	*rugosum*	(L.) All.	0	0	0	0	1
Asteraceae	*Reichardia*	*picroides*	(L.) Roth	0	1	0	0	0
Resedaceae	*Reseda*	*lutea*	L.	0	3	0	0	5
Resedaceae	*Reseda*	*odorata*	L.	0	1	0	0	0
Polygonaceae	*Reynoutria*	*japonica*	Houtt.	1	0	0	0	0
Rosaceae	*Rhaphiolepis*	*indica*	(L.) Lindl.	0	2	0	0	0
Orobanchaceae	*Rhinanthus*	*minor*	L.	0	0	0	0	12
Crassulaceae	*Rhodiola*	*ishidae*	Hara	0	0	0	0	1
Ericaceae	*Rhododendron*	*adenogynum*	Diels	0	0	0	0	1
Ericaceae	*Rhododendron*	*ambiguum*	Hemsl.	0	0	0	0	1
Ericaceae	*Rhododendron*	*annae*	Franch.	0	1	0	0	0
Ericaceae	*Rhododendron*	*auriculatum*	Hemsl.	0	0	0	0	1
Ericaceae	*Rhododendron*	*canadense*	(L.) Torr.	0	1	0	0	0
Ericaceae	*Rhododendron*	Catawbiense Group		2	0	1	0	12
Ericaceae	*Rhododendron*	*cerasinum*	Tagg	0	0	0	0	1
Ericaceae	*Rhododendron*	*cyanocarpum*	W. Sm.	0	0	0	0	1
Ericaceae	*Rhododendron*	*dauricum*	L.	0	0	0	0	1
Ericaceae	*Rhododendron*	*degronianum*	Carrière	0	1	0	0	0

Ericaceae	*Rhododendron*	*fortunei*	Lindl.	0	0	0	0	1
Ericaceae	*Rhododendron*	*hirsutum*	L.	0	1	0	0	1
Ericaceae	*Rhododendron*	*hirtipes*	Tagg	0	0	0	0	2
Ericaceae	*Rhododendron*	*insigne*	Hemsl. & E. H. Wilson	0	0	0	0	1
Ericaceae	*Rhododendron*	*maximum*	L.	0	1	0	0	0
Ericaceae	*Rhododendron*	*mucronulatum subsp. Sichotense*	(Pojark.) A. P. Khokhr.	0	0	0	0	1
Ericaceae	*Rhododendron*	*obtusum*	Hort. ex Wats.	0	1	0	0	1
Ericaceae	*Rhododendron*	*orbiculare*	Decne.	0	0	0	0	1
Ericaceae	*Rhododendron*	*ponticum*	L.	5	2	1	0	2
Ericaceae	*Rhododendron*	*smirnowii*	Trautv. ex Regel	0	0	0	0	1
Ericaceae	*Rhododendron*	sp.		0	4	2	0	10
Ericaceae	*Rhododendron*	*thomsonii*	Hook. f.	0	0	0	0	5
Ericaceae	*Rhododendron*	*xanthocodon*	Hutch.	0	0	0	0	1
Rosaceae	*Rhodotypos*	*scandens*	(Thunb.) Makino	0	1	0	0	0
Anacardiaceae	*Rhus*	*integrifolia*	(Nutt.) Benth. & Hook. f. ex Rothr.	0	0	1	0	0
Anacardiaceae	*Rhus*	*typhina*	L.	0	1	0	0	0
Grossulariaceae	*Ribes*	*alpinum*	L.	0	0	0	1	7
Grossulariaceae	*Ribes*	*nigrum*	L.	0	0	0	1	0
Grossulariaceae	*Ribes*	*petraeum*	Wulfen	0	0	0	0	7
Grossulariaceae	*Ribes*	*rubrum*	L.	0	0	0	0	2
Grossulariaceae	*Ribes*	*sanguineum*	Pursh	0	1	0	0	1
Grossulariaceae	*Ribes*	*speciosum*	Pursh	0	1	0	0	0

Grossulariaceae	*Ribes*	*uva-crispa*	L.	0	0	0	0	1
Euphorbiaceae	*Ricinus*	*communis*	L.	0	0	0	0	3
Fabaceae	*Robinia*	*hispida*	L.	0	0	0	0	1
Fabaceae	*Robinia*	*neomexicana*	A.Gray	0	1	0	0	0
Fabaceae	*Robinia*	*pseudoacacia*	L.	3	3	5	0	5
Fabaceae	*Robinia*	*× ambigua*	Poir.	0	1	0	0	0
Saxifragaceae	*Rodgersia*	*podophylla*	A.Gray	0	0	0	0	2
Saxifragaceae	*Rodgersia*	*sambucifolia*	Hemsl.	0	0	0	0	1
Papaveraceae	*Roemeria*	*hybrida*	(L.) DC.	0	1	0	0	0
Papaveraceae	*Romneya*	*coulteri*	Harv.	0	1	0	0	0
Brassicaceae	*Rorippa*	*amphibia*	(L.) Besser	1	0	0	0	0
Brassicaceae	*Rorippa*	*sylvestris*	(L.) Besser	1	0	2	0	5
Rosaceae	*Rosa*	*agrestis*	Savi	0	1	0	0	0
Rosaceae	*Rosa*	*canina* aggr.	L.	2	2	2	0	17
Rosaceae	*Rosa*	*chinensis*	Jacq.	0	2	0	0	0
Rosaceae	*Rosa*	*gallica*	L.	0	1	0	0	0
Rosaceae	*Rosa*	*multiflora*	Thunb.	0	0	0	0	2
Rosaceae	*Rosa*	*omeiensis*	Rolfe	0	1	0	0	0
Rosaceae	*Rosa*	*rugosa*	Thunb.	0	0	0	0	1
Rosaceae	*Rosa*	CV		11	12	5	6	22
Rosaceae	*Rosa*	*spinosissima*	L.	1	2	0	0	10
Rosaceae	*Rosa*	*× damascena*	Herrm.	0	1	0	0	1
Lamiaceae	*Rosmarinus*	*officinalis*	L.	0	1	0	0	15
Rubiaceae	*Rubia*	*tinctorum*	L.	0	0	0	0	1
Rosaceae	*Rubus*	*caesius*	L.	0	2	3	0	0
Rosaceae	*Rubus*	*cockburnianus*	Hemsl	0	0	0	0	1

Rosaceae	*Rubus*	*fruticosus* aggr.		7	5		1	0	27
Rosaceae	*Rubus*	*idaeus*	L.	1	0		0	1	6
Rosaceae	*Rubus*	*odoratus*	L.	1	0		0	0	0
Rosaceae	*Rubus*	*saxatilis*	L.	0	0		0	0	1
Asteraceae	*Rudbeckia*	*fulgida*	Aiton	0	1		0	0	0
Asteraceae	*Rudbeckia*	*hirta*	L.	1	5		0	1	14
Asteraceae	*Rudbeckia*	*laciniata*	L.	1	0		1	0	2
Asteraceae	*Rudbeckia*	*maxima*	Nutt.	0	1		0	0	0
Acanthaceae	*Ruellia*	*ciliosa*	Pursh	0	1		0	0	0
Polygonaceae	*Rumex*	*acetosella*	L.	2	1		0	0	0
Polygonaceae	*Rumex*	*conglomeratus*	Murray	0	1		0	0	0
Polygonaceae	*Rumex*	*crispus*	L.	0	4		0	0	0
Polygonaceae	*Rumex*	*obtusifolius*	L.	0	1		0	0	0
Polygonaceae	*Rumex*	*salicifolius*	Weinm.	0	1		0	0	0
Polygonaceae	*Rumex*	sp.		1	3		0	0	0
Rutaceae	*Ruta*	*chalepensis*	L.	0	1		0	0	0
Rutaceae	*Ruta*	*graveolens*	L.	0	1		0	1	3
Salicaceae	*Salix*	*alba*	L.	0	0		0	1	7
Salicaceae	*Salix*	*alpina*	Scop.	0	0		0	0	1
Salicaceae	*Salix*	*babylonica*	L.	0	0		0	0	2
Salicaceae	*Salix*	*caesia*	Vill.	0	0		0	0	1
Salicaceae	*Salix*	*caprea*	L.	0	0		0	2	5
Salicaceae	*Salix*	*cinerea*	L.	0	0		0	0	1
Salicaceae	*Salix*	*elaeagnos*	Scop.	0	0		0	0	1
Salicaceae	*Salix*	*fragilis*	L.	0	0		0	1	0
Salicaceae	*Salix*	*fruticulosa*	Andrersson	0	0		0	0	1

Salicaceae	*Salix*	sp.		0	0	0	0	1
Lamiaceae	*Salvia*	*africana-lutea*	L.	0	0	0	0	1
Lamiaceae	*Salvia*	*apiana*	Jeps.	0	0	0	0	3
Lamiaceae	*Salvia*	*bulleyana*	Diels	0	0	0	0	1
Lamiaceae	*Salvia*	*candelabrum*	Boiss.	0	1	0	0	0
Lamiaceae	*Salvia*	*carnea*	Kunth	0	0	0	0	1
Lamiaceae	*Salvia*	*clevelandii*	(A. Gray) Greene	0	0	0	0	1
Lamiaceae	*Salvia*	*coccinea*	Buc'hoz ex Etl.	0	1	0	0	0
Lamiaceae	*Salvia*	*darcyi*	J. Compton	0	0	0	0	1
Lamiaceae	*Salvia*	*discolor*	Kunth	0	0	0	0	1
Lamiaceae	*Salvia*	*elegans*	Vahl	0	0	0	0	2
Lamiaceae	*Salvia*	*farinacea*	Benth.	3	2	0	0	8
Lamiaceae	*Salvia*	*fruticosa*	Mill.	0	1	0	0	0
Lamiaceae	*Salvia*	*glutinosa*	L.	0	1	0	0	2
Lamiaceae	*Salvia*	*greggii*	A.Gray	0	0	0	0	2
Lamiaceae	*Salvia*	*heerii*	Regel	0	0	0	0	2
Lamiaceae	*Salvia*	*hispanica*	L.	0	0	0	0	1
Lamiaceae	*Salvia*	*japonica*	Thunb.	0	0	0	0	1
Lamiaceae	*Salvia*	*microphylla*	Kunth	0	3	0	0	0
Lamiaceae	*Salvia*	*miltiorrhiza*	Bunge	0	1	0	0	0
Lamiaceae	*Salvia*	*nemorosa*	L.	2	1	0	0	4
Lamiaceae	*Salvia*	*nutans*	L.	0	1	0	0	0
Lamiaceae	*Salvia*	*officinalis*	L.	2	2	0	0	11
Lamiaceae	*Salvia*	*officinalis subsp. lavandulifolia*	(Vahl) Gams	0	1	0	0	0

Lamiaceae	*Salvia*	*oligantha*	Dusén	0	0		0	0	1
Lamiaceae	*Salvia*	*oxyphora*	Briq.	0	0		0	0	1
Lamiaceae	*Salvia*	*patens*	Cav.	0	2		0	0	1
Lamiaceae	*Salvia*	*pauciserrata*	Benth.	0	0		0	0	1
Lamiaceae	*Salvia*	*polystachya*	Cav.	1	0		0	0	1
Lamiaceae	*Salvia*	*pratensis*	L.	2	4		0	0	19
Lamiaceae	*Salvia*	*sclarea*	L.	0	1		0	0	0
Lamiaceae	*Salvia*	*somalensis*	Vatke	0	0		0	0	1
Lamiaceae	*Salvia*	sp.		0	0		0	0	3
Lamiaceae	*Salvia*	*splendens*	Sellow ex Schult.	1	6		1	0	0
Lamiaceae	*Salvia*	*subpalmatinervis*	E.Peter	0	0		0	0	1
Lamiaceae	*Salvia*	*taraxacifolia*	Coss. & Balansa	0	1		0	0	0
Lamiaceae	*Salvia*	*verbenaca*	L.	0	0		0	0	1
Lamiaceae	*Salvia*	*verticillata*	L.	0	1		0	0	2
Lamiaceae	*Salvia*	*viridis*	L.	0	0		0	0	3
Lamiaceae	*Salvia*	*× jamensis*	J. Compton	0	0		0	0	2
Adoxaceae	*Sambucus*	*ebulus*	L.	0	1		0	0	1
Adoxaceae	*Sambucus*	*nigra*	L.	2	7		3	0	17
Adoxaceae	*Sambucus*	*racemosa*	L.	2	0		0	2	0
Primulaceae	*Samolus*	*valerandi*	L.	0	1		0	0	1
Rosaceae	*Sanguisorba*	*canadensis*	L.	0	1		0	0	0
Rosaceae	*Sanguisorba*	*minor*	Scop.	0	2		0	0	12
Rosaceae	*Sanguisorba*	*officinalis*	L.	0	0		0	0	5
Asteraceae	*Santolina*	*chamaecyparissus*	L.	0	1		0	0	2
Asteraceae	*Santolina*	*rosmarinifolia*	L	0	0		0	0	1

Asteraceae	*Sanvitalia*	*procumbens*	Lam.	0	1	0	0	7
Caryophyllaceae	*Saponaria*	*caespitosa*	DC.	0	1	0	0	0
Caryophyllaceae	*Saponaria*	*lutea*	L.	0	1	0	0	0
Caryophyllaceae	*Saponaria*	*ocymoides*	L.	0	0	0	0	1
Caryophyllaceae	*Saponaria*	*officinalis*	L.	0	1	3	1	6
Sarraceniaceae	*Sarracenia*	*flava*	L.	0	0	0	0	1
Sarraceniaceae	*Sarracenia*	*leucophylla*	Raf.	0	0	0	0	1
Sarraceniaceae	*Sarracenia*	*oreophila*	Wherry	0	0	0	0	1
Sarraceniaceae	*Sarracenia*	*purpurea*	L.	0	1	0	0	1
Lamiaceae	*Satureja*	*montana*	L.	0	0	0	0	1
Saururaceae	*Saururus*	*cernuus*	L.	0	0	0	0	1
Saxifragaceae	*Saxifraga*	*adscendens*	L.	0	0	0	0	1
Saxifragaceae	*Saxifraga*	*aizoides*	L.	0	0	0	0	1
Saxifragaceae	*Saxifraga*	*callosa*	Sm.	0	1	0	0	0
Saxifragaceae	*Saxifraga*	*cochlearis*	Rchb.	0	1	0	0	0
Saxifragaceae	*Saxifraga*	*cotyledon*	L.	0	1	0	0	0
Saxifragaceae	*Saxifraga*	*crustata*	Vest	0	1	0	0	0
Saxifragaceae	*Saxifraga*	*cuneifolia*	L.	0	0	0	0	1
Saxifragaceae	*Saxifraga*	*cymbalaria*	L.	0	1	0	0	1
Saxifragaceae	*Saxifraga*	*granulata*	L.	1	1	1	0	0
Saxifragaceae	*Saxifraga*	*hostii*	Tausch	0	1	0	0	1
Saxifragaceae	*Saxifraga*	*hypnoides*	L.	0	1	0	0	0
Saxifragaceae	*Saxifraga*	*paniculata*	Mill.	0	1	0	0	1
Saxifragaceae	*Saxifraga*	*retusa* subsp. *augustana*	(Vacc.) P. Fourn.	0	1	0	0	0
Saxifragaceae	*Saxifraga*	*rosacea*	Moench	0	1	0	0	0

Saxifragaceae	*Saxifraga*	*rotundifolia*	L.	0	0	0	0	1
Saxifragaceae	*Saxifraga*	*seguieri*	Spreng.	0	0	0	0	1
Saxifragaceae	*Saxifraga*	sp.		1	0	0	0	7
Saxifragaceae	*Saxifraga*	*stolonifera*	Meerb.	0	1	0	0	0
Saxifragaceae	*Saxifraga*	*tridactylites*	L.	1	1	0	0	6
Saxifragaceae	*Saxifraga*	*trifurcata*	Schrad.	0	1	0	0	0
Saxifragaceae	*Saxifraga*	*umbrosa*	L.	0	1	0	0	1
Saxifragaceae	*Saxifraga*	*× gaudinii*	Brügger	0	1	0	0	0
Caprifoliaceae	*Scabiosa*	*cinerea*	Lapeyr. ex Lam.	0	1	0	0	0
Caprifoliaceae	*Scabiosa*	*columbaria*	L.	0	1	0	0	9
Caprifoliaceae	*Scabiosa*	*graminifolia*	L.	0	1	0	0	0
Caprifoliaceae	*Scabiosa*	*lucida*	Vill.	0	1	0	0	1
Caprifoliaceae	*Scabiosa*	*ochroleuca*	L.	0	1	0	0	1
Asparagaceae	*Scilla*	*siberica*	Haw.	0	0	0	9	1
Asteraceae	*Scolymus*	*hispanicus*	L.	0	1	0	0	0
Fabaceae	*Scorpiurus*	*muricatus*	L.	0	1	0	0	0
Scrophulariaceae	*Scrophularia*	*alpestris*	J. Gay ex Benth.	0	1	0	0	0
Scrophulariaceae	*Scrophularia*	*auriculata*	L.	0	1	0	0	0
Scrophulariaceae	*Scrophularia*	*canina*	L.	0	1	0	0	0
Scrophulariaceae	*Scrophularia*	*juratensis*	Schleich.	0	0	0	0	1
Scrophulariaceae	*Scrophularia*	*nodosa*	L.	0	0	0	0	1
Scrophulariaceae	*Scrophularia*	*scorodonia*	L.	0	1	0	0	0
Scrophulariaceae	*Scrophularia*	*vernalis*	L.	0	1	0	0	0
Lamiaceae	*Scutellaria*	*alpina*	L.	0	1	0	0	0
Lamiaceae	*Scutellaria*	*altissima*	L.	0	2	0	0	3
Lamiaceae	*Scutellaria*	*aurantiaca*	A. Pool	0	0	0	0	1

Lamiaceae	*Scutellaria*	*caucasica*	A. Ham.	0	1	0	0	0
Lamiaceae	*Scutellaria*	*columnae*	All.	0	1	0	0	0
Lamiaceae	*Scutellaria*	*galericulata*	L.	0	1	0	0	2
Cucurbitaceae	*Sechium*	*edule*	(Jacq.) Sw.	0	0	0	0	1
Fabaceae	*Securigera*	*varia*	(L.) Lassen	0	3	3	0	3
Crassulaceae	*Sedum*	*acre*	L.	1	2	2	3	6
Crassulaceae	*Sedum*	*aizoon*	L.	0	1	0	0	1
Crassulaceae	*Sedum*	*album*	L.	0	1	0	3	9
Crassulaceae	*Sedum*	*anacampseros*	L.	0	0	0	0	1
Crassulaceae	*Sedum*	*cepaea*	L.	0	1	0	0	0
Crassulaceae	*Sedum*	*dasyphyllum*	L.	0	1	0	0	0
Crassulaceae	*Sedum*	*hispanicum*	L.	0	1	0	0	0
Crassulaceae	*Sedum*	*kamtschaticum*	Fisch.	1	1	0	0	4
Crassulaceae	*Sedum*	*moranense*	Kunth	0	1	0	0	0
Crassulaceae	*Sedum*	*ochroleucum*	Chaix	0	0	0	0	1
Crassulaceae	*Sedum*	*rupestre*	L.	0	3	0	0	7
Crassulaceae	*Sedum*	*sediforme*	(Jacq.) Pau	0	1	0	0	1
Crassulaceae	*Sedum*	*sexangulare*	L.	0	1	0	0	13
Crassulaceae	*Sedum*	*spurium*	M. Bieb.	0	1	0	0	1
Crassulaceae	*Sedum*	*stoloniferum*	S. G. Gmel.	0	1	0	0	0
Crassulaceae	*Sedum*	*telephium*	L.	0	0	0	0	11
Crassulaceae	*Sempervivum*	*arachnoideum*	L.	0	1	0	0	2
Crassulaceae	*Sempervivum*	*calcareum*	Jord.	0	1	0	0	0
Crassulaceae	*Sempervivum*	*grandiflorum*	Haw.	0	0	0	0	1
Crassulaceae	*Sempervivum*	*marmoreum*	Griseb.	0	0	0	0	1
Crassulaceae	*Sempervivum*	*montanum*	L.	0	1	0	0	0

Crassulaceae	*Sempervivum*	*tectorum*	L.	0	1	0	0	2
Crassulaceae	*Sempervivum*	*× christii*	W. Wolf	0	0	0	0	1
Asteraceae	*Senecio*	*alpinus*	(L.) Scop.	0	1	0	0	1
Asteraceae	*Senecio*	*aquaticus*	Hill	0	0	0	0	1
Asteraceae	*Senecio*	*inaequidens*	DC.	2	7	4	0	6
Asteraceae	*Senecio*	*ovatus*	(P. Gaertn. & al.) Willd.	0	0	0	0	1
Asteraceae	*Senecio*	*vulgaris*	L.	7	10	1	5	25
Fabaceae	*Senna*	*didymobotrya*	Barneby	0	1	0	0	0
Fabaceae	*Senna*	*occidentalis*	(L.) Link	0	1	0	0	0
Asteraceae	*Serratula*	*tinctoria*	L.	0	0	0	0	1
Apiaceae	*Seseli*	*gummiferum*	Pall. ex Sm.	0	1	0	0	0
Apiaceae	*Seseli*	*libanotis*	(L.) W. D. J. Koch	0	1	0	0	0
Apiaceae	*Seseli*	*montanum*	L.	0	1	0	0	0
Rubiaceae	*Sherardia*	*arvensis*	L.	0	4	0	0	5
Malvaceae	*Sida*	*hermaphrodita*	(L.) Rusby	0	1	0	0	0
Lamiaceae	*Sideritis*	*hyssopifolia*	L.	1	1	0	0	0
Caryophyllaceae	*Silene*	*alpestris*	Jacq.	0	1	0	0	0
Caryophyllaceae	*Silene*	*armeria*	L.	0	1	0	0	7
Caryophyllaceae	*Silene*	*caucasica*	Boiss.	0	1	0	0	0
Caryophyllaceae	*Silene*	*chalcedonica*	(L.) E. H. L. Krause	0	0	0	4	3
Caryophyllaceae	*Silene*	*coeli-rosa*	(L.) Godr.	0	1	0	0	0
Caryophyllaceae	*Silene*	*coronaria*	(L.) Clairv.	2	1	1	0	7
Caryophyllaceae	*Silene*	*dichotoma*	Ehrh.	0	0	0	0	3
Caryophyllaceae	*Silene*	*dioica*	(L.) Clairv.	0	1	0	0	9

Caryophyllaceae	*Silene*	*flos-cuculi*	(L.) Clairv.	2	2	0	2	12
Caryophyllaceae	*Silene*	*flos-jovis*	(L.) Clairv.	0	0	0	0	1
Caryophyllaceae	*Silene*	*gallica*	L.	0	1	0	0	0
Caryophyllaceae	*Silene*	*italica*	(L.) Pers.	0	1	0	0	0
Caryophyllaceae	*Silene*	*latifolia*	Poir.	0	6	0	0	0
Caryophyllaceae	*Silene*	*latifolia subsp. alba*	(Mill.) Greuter & Burdet	0	2	4	7	0
Caryophyllaceae	*Silene*	*multiflora*	(Ehrh.) Pers.	0	0	0	0	1
Caryophyllaceae	*Silene*	*nutans*	L.	0	1	2	0	11
Caryophyllaceae	*Silene*	*pratensis*	(Rafn) Godr.	1	0	1	1	7
Caryophyllaceae	*Silene*	*schafta*	J.G.Gmel. ex Hohen.	0	1	0	0	0
Caryophyllaceae	*Silene*	sp.		1	0	0	0	0
Caryophyllaceae	*Silene*	*uniflora*	Roth	0	1	0	0	0
Caryophyllaceae	*Silene*	*vallesia*	L.	0	1	0	0	0
Caryophyllaceae	*Silene*	*viridiflora*	L.	0	1	0	0	0
Caryophyllaceae	*Silene*	*viscaria*	(L.) Borkh.	0	1	0	0	1
Caryophyllaceae	*Silene*	*vulgaris*	(Moench) Garcke	0	1	2	2	17
Caryophyllaceae	*Silene*	*yunnanensis*	Franch.	0	0	0	0	1
Asteraceae	*Silphium*	*trifoliatum*	L.	0	1	0	0	0
Asteraceae	*Silybum*	*marianum*	(L.) Gaertn.	0	1	0	0	2
Brassicaceae	*Sinapis*	*alba*	L.	0	1	1	0	1
Brassicaceae	*Sinapis*	*arvensis*	L.	0	2	1	0	11
Brassicaceae	*Sisymbrium*	*altissimum*	L.	0	0	0	0	1
Brassicaceae	*Sisymbrium*	*irio*	L.	0	4	0	0	0

Brassicaceae	*Sisymbrium*	*officinale*	(L.) Scop.	1	6	0	0	7
Brassicaceae	*Sisymbrium*	*orientale*	L.	0	0	0	0	1
Iridaceae	*Sisyrinchium*	*angustifolium*	Mill.	0	1	0	0	0
Iridaceae	*Sisyrinchium*	*californicum*	(Ker Gawl.) Dryand.	0	1	0	0	0
Iridaceae	*Sisyrinchium*	*campestre*	E. P. Bicknell	0	1	0	0	0
Iridaceae	*Sisyrinchium*	*elmeri*	Greene	0	1	0	0	0
Iridaceae	*Sisyrinchium*	*idahoense*	E. P. Bicknell	0	1	0	0	0
Iridaceae	*Sisyrinchium*	*langloisii*	Greene	0	1	0	0	0
Iridaceae	*Sisyrinchium*	*montanum*	Greene	0	1	0	0	0
Iridaceae	*Sisyrinchium*	*palmifolium*	L.	0	1	0	0	0
Iridaceae	*Sisyrinchium*	*pearcei*	Phil.	0	1	0	0	0
Iridaceae	*Sisyrinchium*	*striatum*	Sm.	0	2	0	0	0
Iridaceae	*Sisyrinchium*	*tinctorium*	Kunth	0	1	0	0	0
Apiaceae	*Sium*	*latifolium*	L.	0	0	0	0	1
Apiaceae	*Sium*	*sisarum*	L.	0	1	0	0	1
Solanaceae	*Solanum*	*abutiloides*	(Gruseb.) Bitter & Lillo	0	0	0	0	1
Solanaceae	*Solanum*	*aethiopicum*	L.	0	0	0	0	1
Solanaceae	*Solanum*	*betaceum*	Cav.	0	0	0	0	1
Solanaceae	*Solanum*	*chenopodioides*	Lam.	0	1	0	0	0
Solanaceae	*Solanum*	*citrullifolium*	A. Braun	0	1	0	0	0
Solanaceae	*Solanum*	*crispum*	Ruiz & Pav.	0	1	0	0	0
Solanaceae	*Solanum*	*dulcamara*	L.	2	7	0	0	8
Solanaceae	*Solanum*	*jasminoides*	J. Paxton	0	1	0	0	0
Solanaceae	*Solanum*	*laxum*	Spreng.	0	1	0	0	0

Solanaceae	*Solanum*	*melongena*	L.	0	0	0	0	3
Solanaceae	*Solanum*	*nigrum*	L.	1	6	0	0	13
Solanaceae	*Solanum*	*pseudocapsicum*	L.	0	1	0	0	0
Solanaceae	*Solanum*	*quitoense*	Lam.	0	0	0	0	1
Solanaceae	*Solanum*	*sisymbriifolium*	Lam.	0	1	0	0	0
Solanaceae	*Solanum*	*tuberosum*	L.	0	0	0	0	5
Boraginaceae	*Solenanthus*	*circinatus*	Ledeb.	0	1	0	0	0
Iridaceae	*Solenomelus*	*segethi*	(Phil.) Kuntze	0	1	0	0	0
Asteraceae	*Solidago*	*canadensis* aggr.	L.	2	2	7	8	7
Asteraceae	*Solidago*	*rigida*	L.	0	1	0	0	0
Asteraceae	*Solidago*	*virgaurea*	L.	1	1	0	2	1
Asteraceae	*Sonchus*	*arvensis*	L.	0	4	0	7	6
Asteraceae	*Sonchus*	*asper*	Hill	1	5	0	0	27
Asteraceae	*Sonchus*	*oleraceus*	L.	3	12	2	2	25
Asteraceae	*Sonchus*	*palustris*	L.	0	1	0	0	0
Asteraceae	*Sonchus*	sp.		0	1	0	0	0
Rosaceae	*Sorbaria*	*grandiflora*	(Sweet) Maxim.	0	1	0	0	0
Rosaceae	*Sorbaria*	*kirilowii*	(Regel & Tiling) Maxim.	0	1	0	0	0
Rosaceae	*Sorbaria*	*sorbifolia*	(L.) A. Braun	2	1	0	0	1
Rosaceae	*Sorbus*	*aucuparia*	L.	0	0	0	0	4
Rosaceae	*Sorbus*	*hohenesteri*	N. Mey.	0	0	0	0	1
Typhaceae	*Sparganium*	*erectum*	L.	0	0	0	0	1
Fabaceae	*Spartium*	*junceum*	L.	0	2	0	0	4
Caryophyllaceae	*Spergula*	*arvensis*	L.	0	1	0	0	0
Caryophyllaceae	*Spergularia*	*rubra*	(L.) J. & C. Presl	0	1	0	0	0

Rubiaceae	*Spermacoce*	*verticillata*	L.	1	0	0	0	0
Rosaceae	*Spiraea*	*alba*	Du Roi	0	2	0	0	0
Rosaceae	*Spiraea*	*bullata*	Maxim.	0	0	0	0	1
Rosaceae	*Spiraea*	*cantoniensis*	Lour.	0	0	0	0	2
Rosaceae	*Spiraea*	*chamaedryfolia*	L.	0	2	0	2	0
Rosaceae	*Spiraea*	*douglasii*	Hook.	1	2	0	0	0
Rosaceae	*Spiraea*	*japonica*	L. f.	0	6	4	0	10
Rosaceae	*Spiraea*	*lobata*	Zabel	0	1	0	0	0
Rosaceae	*Spiraea*	*myrtilloides*	Rehder	0	1	0	0	0
Rosaceae	*Spiraea*	*nipponica*	Maxim.	0	2	0	0	2
Rosaceae	*Spiraea*	*salicifolia*	L.	0	1	0	1	0
Rosaceae	*Spiraea*	*splendens*	Baumann ex K. Koch	0	0	1	0	12
Rosaceae	*Spiraea*	*thunbergii*	Siebold ex Blume	0	1	0	0	0
Rosaceae	*Spiraea*	*triloba*	Murray	0	2	0	0	0
Rosaceae	*Spiraea*	*× cinerea*	Zabel	0	0	0	0	7
Araceae	*Spirodela*	*polyrhiza*	(L.) Schleid.	0	0	0	0	1
Amaryllidaceae	*Sprekelia*	*formosissima*	(L.) Herb.	0	1	0	0	0
Lamiaceae	*Stachys*	*affinis*	Bunge	0	0	0	0	1
Lamiaceae	*Stachys*	*alopecuros*	(L.) Benth.	0	1	0	0	0
Lamiaceae	*Stachys*	*alpina*	L.	0	1	0	0	0
Lamiaceae	*Stachys*	*annua*	(L.) L.	0	1	0	0	0
Lamiaceae	*Stachys*	*arvensis*	(L.) L.	1	0	0	0	0
Lamiaceae	*Stachys*	*byzantina*	K. Koch	1	1	0	0	0
Lamiaceae	*Stachys*	*germanica*	L.	0	1	0	0	0

Lamiaceae	*Stachys*	*macrantha*	(K. Koch) Stearn	0	1		0	0	0
Lamiaceae	*Stachys*	*menthifolia*	Vis.	0	1		0	0	0
Lamiaceae	*Stachys*	*officinalis*	(L.) Trevis.	0	1		0	1	7
Lamiaceae	*Stachys*	*palustris*	L.	0	0		0	0	1
Lamiaceae	*Stachys*	*recta*	L.	0	1		0	0	0
Lamiaceae	*Stachys*	*sylvatica*	L.	0	1		0	0	9
Staphyleaceae	*Staphylea*	sp.		0	0		0	0	9
Staphyleaceae	*Staphylea*	*pinnata*	L.	0	0		0	0	5
Staphyleaceae	*Staphylea*	*trifolia*	L.	0	0		0	0	1
Caryophyllaceae	*Stellaria*	*graminea*	L.	0	0		0	4	0
Caryophyllaceae	*Stellaria*	*holostea*	L.	2	0		0	0	2
Caryophyllaceae	*Stellaria*	*media*	(L.) Vill.	5	5		1	5	13
Caryophyllaceae	*Stellaria*	*neglecta*	Weihe	0	2		0	0	0
Rosaceae	*Stephanandra*	*incisa*	(Thunb.) Zabel	0	0		0	0	2
Amaryllidaceae	*Sternbergia*	*lutea*	(L.) Ker Gawl. ex Spreng.	0	1		0	0	0
Asteraceae	*Stevia*	*rebaudiana*	(Bertoni) Bertoni	0	0		0	0	1
Theaceae	*Stewartia*	*pseudocamellia*	Maxim.	0	1		0	0	0
Asteraceae	*Stokesia*	*laevis*	(Hill) Greene	0	1		0	0	0
Strelitziaceae	*Strelitzia*	*reginae*	Banks	1	0		0	0	1
Gesneriaceae	*Streptocarpus*	*saxorum*	Engl.	0	1		0	0	0
Fabaceae	*Styphnolobium*	*japonicum*	(L.) Schott	0	3		0	0	3
Styracaceae	*Styrax*	*japonicus*	Siebold & Zucc.	0	0		0	0	1
Caprifoliaceae	*Succisa*	*pratensis*	Moench	1	1		0	0	1
Caprifoliaceae	*Symphoricarpos*	*albus*	(L.) S. F. Blake	5	1		3	0	10

Asteraceae	*Aster*	*novi-belgii*	(L.) G.L.Nesom	0	1	0	1	1
Boraginaceae	*Symphytum*	*asperum*	Lepech.	0	2	0	0	0
Boraginaceae	*Symphytum*	*bulbosum*	K. F. Schimp.	0	0	0	0	1
Boraginaceae	*Symphytum*	*officinale*	L.	3	3	0	2	4
Boraginaceae	*Symphytum*	*tuberosum*	L.	1	0	0	0	5
Araceae	*Symplocarpus*	*foetidus*	(L.) Salisb. ex W.P.C.Barton	0	0	0	0	1
Oleaceae	*Syringa*	*emodi*	Wall. ex Royle	0	1	0	0	0
Oleaceae	*Syringa*	*tomentella*	Bureau & Franch.	0	1	0	0	0
Oleaceae	*Syringa*	*swegionzowii*	Y.Chen & D.Y.Hong	0	1	0	0	0
Oleaceae	*Syringa*	*vulgaris*	L.	3	3	4	1	14
Myrtaceae	*Syzygium*	*aromaticum*	(L.) Merr. & L. M. Perry	0	0	0	0	1
Asteraceae	*Tagetes*	*erecta*	L.	2	2	5	3	18
Asteraceae	*Tagetes*	*lucida*	Cav.	0	1	2	0	4
Asteraceae	*Tagetes*	*tenuifolia*	Cav	4	0	0	1	6
Tamaricaceae	*Tamarix*	*gallica*	L.	0	0	0	0	2
Tamaricaceae	*Tamarix*	*parviflora*	DC.	0	1	0	0	0
Tamaricaceae	*Tamarix*	*tetrandra*	Pall. ex M. Bieb.	0	0	0	0	1
Asteraceae	*Tanacetum*	*balsamita*	L.	0	0	0	0	1
Asteraceae	*Tanacetum*	*cinerariifolium*	(Trevir.) Sch. Bip.	0	1	0	0	0
Asteraceae	*Tanacetum*	*corymbosum*	(L.) Sch. Bip.	0	0	0	0	1
Asteraceae	*Tanacetum*	*macrophyllum*	(Waldst. & Kit.) Sch. Bip.	0	1	0	0	1

Asteraceae	*Tanacetum*	*parthenium*	(L.) Sch. Bip.	0	2	0	0	2
Asteraceae	*Tanacetum*	*vulgare*	L.	2	1	1	4	3
Asteraceae	*Taraxacum*	*officinale* aggr.		5	11	10	12	32
Asteraceae	*Telekia*	*speciosa*	(Schreb.) Baumg.	0	1	0	0	1
Saxifragaceae	*Tellima*	*grandiflora*	(Pursh) Douglas ex Lindl.	0	1	0	0	1
Lamiaceae	*Teucrium*	*arduinii*	L.	0	1	0	0	0
Lamiaceae	*Teucrium*	*asiaticum*	L.	0	1	0	0	0
Lamiaceae	*Teucrium*	*chamaedrys*	L.	0	2	0	0	1
Lamiaceae	*Teucrium*	*flavum*	L.	0	1	0	0	0
Lamiaceae	*Teucrium*	*fruticans*	L.	0	2	0	0	0
Lamiaceae	*Teucrium*	*hircanicum*	L.	0	1	0	0	0
Lamiaceae	*Teucrium*	*lucidum*	L.	0	1	0	0	0
Lamiaceae	*Teucrium*	*macrum*	Boiss. & Hausskn.	0	0	0	0	1
Lamiaceae	*Teucrium*	*montanum*	L.	0	1	0	0	0
Ranunculaceae	*Thalictrum*	*aquilegiifolium*	L.	0	0	0	0	1
Ranunculaceae	*Thalictrum*	*flavum*	L.	0	1	0	0	3
Ranunculaceae	*Thalictrum*	*lucidum*	L.	0	0	0	0	1
Ranunculaceae	*Thalictrum*	*speciosissimum*	L.	0	1	0	0	0
Fabaceae	*Thermopsis*	*montana*	Torr. & A. Gray	0	1	0	0	0
Cucurbitaceae	*Thladiantha*	*dubia*	Bunge	0	1	0	0	0
Brassicaceae	*Thlaspi*	*alliaceum*	L.	0	0	0	0	1
Brassicaceae	*Thlaspi*	*arvense*	L.	1	1	2	1	4
Brassicaceae	*Thlaspi*	*montanum*	L.	0	0	0	0	1
Acanthaceae	*Thunbergia*	*alata*	Bojer ex Sims	0	1	0	0	4

Acanthaceae	*Thunbergia*	*battiscombei*	Turrill	0	1	0	0	0
Lamiaceae	*Thymus*	*serpyllum* aggr.		0	1	0	0	18
Lamiaceae	*Thymus*	*vulgaris*	L.	0	1	0	0	8
Saxifragaceae	*Tiarella*	*cordifolia*	L.	1	1	0	0	0
Malvaceae	*Tilia*	*cordata*	Mill.	1	0	1	0	5
Malvaceae	*Tilia*	*platyphyllos*	Scop.	3	0	1	2	2
Malvaceae	*Tilia*	*× europaea*	L.	2	0	0	0	4
Asteraceae	*Tithonia*	*rotundifolia*	(Mill.) S.F.Blake	0	0	0	0	1
Tofieldiaceae	*Tofieldia*	*calyculata*	(L.) Wahlenb.	0	0	0	0	1
Apiaceae	*Torilis*	*arvensis*	(Huds.) Link	0	3	0	0	0
Apiaceae	*Torilis*	*japonica*	(Houtt.) DC.	0	0	0	0	2
Apocynaceae	*Trachelospermum*	*asiaticum*	(Siebold & Zucc.) Nakai	0	1	0	0	0
Apocynaceae	*Trachelospermum*	*jasminoides*	(Lindl.) Lem.	1	7	0	0	1
Arecaceae	*Trachycarpus*	*fortunei*	(Hook.) H. Wendl.	0	1	0	0	0
Boraginaceae	*Trachystemon*	*orientalis*	(L.) D.Don	0	0	0	0	1
Commelinaceae	*Tradescantia*	*ohiensis*	Raf.	0	1	0	0	0
Commelinaceae	*Tradescantia*	sp.		0	0	0	0	1
Commelinaceae	*Tradescantia*	*virginiana*	L.	2	2	1	0	5
Asteraceae	*Tragopogon*	*porrifolius*	L.	0	1	0	0	0
Asteraceae	*Tragopogon*	*pratensis*	L.	0	1	0	3	14
Liliaceae	*Tricyrtis*	*formosana*	Baker	0	1	0	0	0
Fabaceae	*Trifolium*	*alpestre*	L.	0	1	0	0	1
Fabaceae	*Trifolium*	*arvense*	L.	0	2	0	0	0
Fabaceae	*Trifolium*	*campestre*	Schreb.	7	0	7	0	32

Fabaceae	*Trifolium*	*dubium*	Sibth.	6	6	5	0	24
Fabaceae	*Trifolium*	*fragiferum*	L.	0	1	0	0	0
Fabaceae	*Trifolium*	*hybridum*	L.	0	1	0	3	7
Fabaceae	*Trifolium*	*medium*	L.	0	0	0	1	0
Fabaceae	*Trifolium*	*montanum*	L.	0	1	0	0	0
Fabaceae	*Trifolium*	*ochroleucon*	Huds.	0	1	0	0	0
Fabaceae	*Trifolium*	*pannonicum*	Jacq.	0	1	0	0	0
Fabaceae	*Trifolium*	*pratense*	L.	3	7	9	12	31
Fabaceae	*Trifolium*	*purpureum*	Loisel.	0	1	0	0	0
Fabaceae	*Trifolium*	*repens*	L.	10	10	11	12	32
Fabaceae	*Trifolium*	*rubens*	L.	0	1	0	0	1
Fabaceae	*Trifolium*	*subterraneum*	L.	0	1	0	0	0
Fabaceae	*Trigonella*	*caerulea*	(L.) Ser.	0	0	0	0	1
Apiaceae	*Trinia*	*glauca*	(L.) Dumort.	0	1	0	0	1
Asteraceae	*Tripleurospermum*	*caucasicum*	(Willd.) Hayek	0	1	0	0	0
Asteraceae	*Tripleurospermum*	*inodorum*	(L.) Sch. Bip.	2	4	0	2	1
Asteraceae	*Tripleurospermum*	*maritimum*	(L.) W.D.J.Koch	0	1	0	0	0
Fabaceae	*Tripodion*	*tetraphyllum*	(L.) Fourr.	0	1	0	0	0
Ranunculaceae	*Trollius*	*chinensis*	Bunge	0	1	0	0	0
Ranunculaceae	*Trollius*	*europaeus*	L.	0	0	0	0	1
Tropaeolaceae	*Tropaeolum*	*ciliatum*	Ruíz & Pav.	0	1	0	0	0
Tropaeolaceae	*Tropaeolum*	*majus*	L.	0	1	0	0	15
Tropaeolaceae	*Tropaeolum*	*peregrinum*	L.	0	1	0	0	0
Liliaceae	*Tulipa*	*gesneriana*	L.	0	0	0	0	1
Liliaceae	*Tulipa*	CV		1	1	0	8	18
Liliaceae	*Tulipa*	*sylvestris*	L.	0	0	0	1	0

Passifloraceae	*Turnera*	*diffusa*	Willd. ex Schult.	0	0	0	0	1
Asteraceae	*Tussilago*	*farfara*	L.	0	0	0	1	0
Typhaceae	*Typha*	*angustifolia*	L.	0	0	0	0	1
Typhaceae	*Typha*	*latifolia*	L.	1	0	0	0	0
Not available	*Not*	*identified*		0	0	0	0	1
Asteraceae	*Urospermum*	*dalechampii*	(L.) Scop. ex F.W.Schmidt	0	1	0	0	0
Urticaceae	*Urtica*	*dioica*	L.	5	8	0	1	0
Lentibulariaceae	*Utricularia*	*australis*	R. Br.	0	0	0	0	1
Caryophyllaceae	*Vaccaria*	*hispanica*	(Mill.) Rauschert	0	1	0	0	5
Ericaceae	*Vaccinium*	*microcarpum*	(Rupr.) Schmalh.	0	0	0	0	1
Ericaceae	*Vaccinium*	*myrtillus*	L.	0	0	0	0	2
Ericaceae	*Vaccinium*	*ovatum*	Pursh	0	1	0	0	0
Ericaceae	*Vaccinium*	*oxycoccos*	L.	0	0	0	0	1
Caprifoliaceae	*Valeriana*	*dioica*	L.	0	0	0	0	1
Caprifoliaceae	*Valeriana*	*officinalis* aggr.	L.	1	2	0	0	3
Caprifoliaceae	*Valeriana*	*pyrenaica*	L.	0	0	0	0	1
Caprifoliaceae	*Valeriana*	*supina*	Ard.	0	0	0	0	1
Caprifoliaceae	*Valeriana*	*tripteris*	L.	0	0	0	0	1
Caprifoliaceae	*Valerianella*	*locusta* aggr.	(L.) Laterr.	0	0	0	0	12
Berberidaceae	*Vancouveria*	*hexandra*	(Hook.) C. Morren & Decne.	0	1	0	0	0
Caryophyllaceae	*Dianthus*	*rigida*	L.	0	1	0	0	0
Melanthiaceae	*Veratrum*	*album*	L.	0	0	0	0	1
Scrophulariaceae	*Verbascum*	*blattaria*	L.	0	1	0	0	0

Scrophulariaceae	*Verbascum*	*chaixii*	Vill.	0	1		0	0	0
Scrophulariaceae	*Verbascum*	*densiflorum*	Bertol.	0	1		0	0	0
Scrophulariaceae	*Verbascum*	*lychnitis*	L.	0	0		0	0	4
Scrophulariaceae	*Verbascum*	*nigrum*	L.	0	2		0	0	5
Scrophulariaceae	*Verbascum*	*phoeniceum*	L.	0	2		0	0	0
Scrophulariaceae	*Verbascum*	*thapsus*	L.	0	1		0	0	2
Verbenaceae	*Verbena*	*bonariensis*	L.	2	3		0	0	8
Verbenaceae	*Verbena*	*officinalis*	L.	0	3		0	0	19
Verbenaceae	*Verbena*	*rigida*	Spreng.	0	4		0	0	0
Verbenaceae	*Verbena*	*× hybrida*	(Groenl. & Rümpler) G. L. Nesom & Pruski	0	0		0	0	1
Asteraceae	*Verbesina*	*alternifolia*	(L.) Britton ex Kearney	0	1		0	0	0
Asteraceae	*Verbesina*	*encelioides*	(Cav.) Benth. & Hook.f. ex A. Gray	0	1		0	0	0
Asteraceae	*Vernonia*	*noveboracensis*	(L.) Michx.	0	1		0	0	0
Plantaginaceae	*Veronica*	*agrestis*	L.	0	7		0	0	0
Plantaginaceae	*Veronica*	*allionii*	Vill.	0	0		0	0	1
Plantaginaceae	*Veronica*	*anagallis-aquatica* aggr.		0	2		0	0	2
Plantaginaceae	*Veronica*	*armena*	Boiss. & A. Huet	0	0		0	0	1
Plantaginaceae	*Veronica*	*arvensis*	L.	1	6		0	2	12
Plantaginaceae	*Veronica*	*austriaca*	L.	0	1		0	0	0
Plantaginaceae	*Veronica*	*beccabunga*	L.	0	2		0	1	2
Plantaginaceae	*Veronica*	*biebersteinii*	Hassk.	0	1		0	0	0

Plantaginaceae	*Veronica*	*chamaedrys*	L.	1	1		5	11	27
Plantaginaceae	*Veronica*	*cupressoides*	Hook. f.	0	0		0	0	1
Plantaginaceae	*Veronica*	*filiformis*	Sm.	0	1		0	3	25
Plantaginaceae	*Veronica*	*fruticulosa*	L.	0	0		0	0	1
Plantaginaceae	*Veronica*	*gentianoides*	Vahl	0	1		0	0	1
Plantaginaceae	*Veronica*	*hederifolia*	L.	1	0		0	0	8
Plantaginaceae	*Veronica*	*longifolia*	L.	2	1		0	1	0
Plantaginaceae	*Veronica*	*ochracea*	(Ashwin) Garn.-Jones	0	1		0	0	1
Plantaginaceae	*Veronica*	*odora*	Hook. f.	0	1		0	0	1
Plantaginaceae	*Veronica*	*officinalis*	L.	0	1		0	0	0
Plantaginaceae	*Veronica*	*opaca*	Fr.	0	1		0	0	0
Plantaginaceae	*Veronica*	*paniculata*	L.	0	1		0	0	0
Plantaginaceae	*Veronica*	*perfoliata*	R. Br.	0	2		0	0	0
Plantaginaceae	*Veronica*	*persica*	Poir.	0	11		0	2	28
Plantaginaceae	*Veronica*	*polita*	Fr.	0	0		0	0	3
Plantaginaceae	*Veronica*	*salicifolia*	G. Forst.	0	1		0	0	0
Plantaginaceae	*Veronica*	*scutellata*	L.	0	0		0	0	1
Plantaginaceae	*Veronica*	*serpyllifolia*	L.	1	2		2	7	24
Plantaginaceae	*Veronica*	sp.		0	0		0	2	0
Plantaginaceae	*Veronica*	*spicata*	L.	1	2		0	0	5
Plantaginaceae	*Veronica*	*spicata* subsp. *orchidea*	(Crantz) Hayek	0	0		0	0	1
Plantaginaceae	*Veronica*	*subalpina*	Cockayne	0	1		0	0	0
Plantaginaceae	*Veronica*	*teucrium*	L.	0	1		0	2	0
Plantaginaceae	*Veronica*	*traversii*	Hook.f.	0	1		0	0	0

Plantaginaceae	*Veronicastrum*	*virginicum*	(L.) Farw.	1	0	0	0	1
Caprifoliaceae	*Vesalea*	*floribunda*	M.Martens & Galeotti	0	1	0	0	0
Solanaceae	*Vestia*	*foetida*	Hoffmanns.	0	1	0	0	0
Adoxaceae	*Viburnum*	*lantana*	L.	1	3	0	0	11
Adoxaceae	*Viburnum*	*odoratissimum*	Ker Gawl.	0	1	0	0	0
Adoxaceae	*Viburnum*	*opulus*	L.	2	5	2	0	15
Adoxaceae	*Viburnum*	*plicatum*	Thunb.	0	3	0	0	0
Adoxaceae	*Viburnum*	*rhytidophyllum*	Hemsl.	0	0	0	0	3
Adoxaceae	*Viburnum*	sp.	CV	0	1	0	0	5
Fabaceae	*Vicia*	*altissima*	Desf.	0	1	0	0	0
Fabaceae	*Vicia*	*cracca*	L.	1	3	1	9	3
Fabaceae	*Vicia*	*faba*	L.	0	0	0	0	4
Fabaceae	*Vicia*	*sativa*	L.	0	6	1	0	32
Fabaceae	*Vicia*	*sepium*	L.	0	0	1	4	26
Fabaceae	*Vicia*	*tetrasperma*	(L.) Schreb.	0	1	0	0	0
Fabaceae	*Vicia*	*villosa*	Roth	0	1	0	0	0
Fabaceae	*Vigna*	*caracalla*	(L.) Verdc.	0	0	0	0	1
Apocynaceae	*Vinca*	*difformis*	Pourr.	0	1	0	0	0
Apocynaceae	*Vinca*	*major*	L.	1	6	0	0	9
Apocynaceae	*Vinca*	*minor*	L.	0	6	0	5	14
Apocynaceae	*Vincetoxicum*	*hirundinaria*	Medik.	0	2	0	0	1
Apocynaceae	*Vincetoxicum*	*nigrum*	(L.) Moench	0	1	0	0	0
Violaceae	*Viola*	*arvensis*	Murray	0	1	0	3	0
Violaceae	*Viola*	*canina*	L.	0	0	0	0	1
Violaceae	*Viola*	*cornuta*	L.	5	2	1	4	17

Violaceae	*Viola*	*hederacea*	Labill.	0	1	0	0	0
Violaceae	*Viola*	*odorata*	L.	0	0	0	4	0
Violaceae	*Viola*	*persicifolia*	Schreb.	0	0	0	0	1
Violaceae	*Viola*	*reichenbachiana*	Boreau	0	0	0	0	8
Violaceae	*Viola*	*riviniana*	Rchb.	0	0	0	0	14
Violaceae	*Viola*	sp.		5	4	0	4	3
Violaceae	*Viola*	*tricolor*	L.	4	7	0	1	16
Violaceae	*Viola*	*× wittrockiana*	Gams	0	0	5	4	13
Lamiaceae	*Vitex*	*agnus-castus*	L.	0	2	0	0	3
Rosaceae	*Waldsteinia*	*geoides*	Willd.	0	0	0	0	5
Rosaceae	*Waldsteinia*	*ternata*	(Stephan) Fritsch	0	0	0	0	1
Caprifoliaceae	*Weigela*	CV		0	7	1	0	6
Fabaceae	*Wisteria*	*floribunda*	(Willd.) DC.	0	1	0	0	0
Fabaceae	*Wisteria*	*sinensis*	(Sims) Sweet	3	3	0	0	10
Solanaceae	*Withania*	*somnifera*	(L.) Dunal	0	0	0	0	1
Plantaginaceae	*Wulfenia*	*baldaccii*	Degen	0	0	0	0	1
Plantaginaceae	*Wulfeniopsis*	*amherstiana*	(Benth.) D. Y. Hong	0	0	0	0	1
Asteraceae	*Xanthium*	*strumarium*	L.	0	0	0	0	1
Asteraceae	*Xeranthemum*	*cylindraceum*	Sm.	0	1	0	0	0
Asteraceae	*Xerochrysum*	*bracteatum*	(Vent.) Tzevelev	0	0	0	0	1
Velloziaceae	*Xerophyta*	*elegans*	(Balf.) Baker	0	1	0	0	0
Asparagaceae	*Yucca*	*filamentosa*	L.	0	1	1	0	10
Asparagaceae	*Yucca*	*gloriosa*	L.	0	1	0	0	0
Amaryllidaceae	*Zephyranthes*	*candida*	(Lindl.) Herb.	0	1	0	0	0
Asteraceae	*Zinnia*	*elegans*	L.	1	3	0	0	10

Asteraceae	*Zinnia*	*haageana*	Regel	0	1		0	0	0
Asteraceae	*Zinnia*	*marylandica*	D. M. Spooner, Stimart & T. H. Boyle	0	1		0	0	0
Asteraceae	*Zinnia*	*peruviana*	(L.) L.	0	1		0	0	0
Zygophyllaceae	*Zygophyllum*	*fabago*	L.	0	1		0	0	0
